# Design and synthesis of novel pyrazolo[4,3-*d*]pyrimidines as potential therapeutic agents for acute lung injury

**DOI:** 10.1080/14756366.2019.1618291

**Published:** 2019-05-23

**Authors:** Bao Shi Wang, Xin Huang, Liu Zeng Chen, Ming Ming Liu, Jing Bo Shi

**Affiliations:** School of Pharmacy, Anhui Province Key Laboratory of Major Autoimmune Diseases, Anhui Institute of Innovative Drugs, Anhui Medical University, Hefei, People's Republic of China

**Keywords:** Pyrazolo[4,3-*d*]pyrimidine, synthesis, anti-inflammatory activity, acute lung injury

## Abstract

Four series of total 35 new pyrazolo[4,3-*d*]pyrimidine compounds were designed, synthesized and evaluated for their inhibitory activity against LPS-induced NO production in RAW264.7 macrophages. Among them, compound **4e** was found to be the most potent inhibitor, which decreased the production of cytokines *in vitro*, such as NO, IL-6 and TNF-α, with IC_50_ values of 2.64, 4.38 and 5.63 μM, respectively. Further studies showed that compound **4e** inhibited cytokines secretion of macrophages through suppressing TLR4/p38 signaling pathway. Additionally, compound **4e** showed *in vivo* anti-inflammatory activity in LPS-induced model of acute lung injury. These data suggested that compound **4e** may be a promising lead structure for the treatment of ALI.

## 1. Introduction

Acute lung injury (ALI), characterized by increased permeability of endothelium and epithelium as well as loss of vascular integrity, is an acute inflammatory disease with high morbidity and mortality^1^^,^^2^. ALI is directly or indirectly caused by pneumonia, inhalation injury, drowning and so forth^2–4^, which clinical manifestations include pulmonary edema, dyspnea, hypoxemia^5–7^. Many therapies for ALI have been conducted, but effective therapeutic agents were not discovered up to now^8^. Recently, several studies have shown that various inflammatory cytokines, such as interleukin-6 (IL-6) and tumor necrosis factor-α (TNF-α), play a pivotal role in the development and progrossion of ALI^9–11^. Increasing evidences showed that suppressing the over-secretion of inflammatory cytokines have been emeraged as a promising strategy for the treatment of ALI^12^^,^^13^.

**Figure 1.**
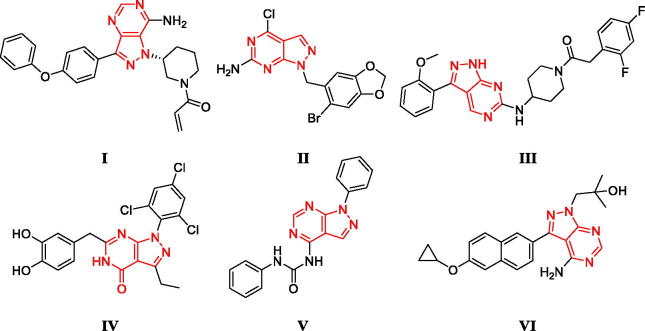
The structures of several pyrazolopyrimidines.

Pyrazolopyrimidine moiety is an important drug-like scaffold^14^, which have shown a wide range of clinical applications including bruton's tyrosine kinase inhibitor ibrutinib (**I**)^15^, tumor necrosis factor receptor-associated protein 1 (TRAP1) inhibitor (**II**)^16^, cyclin-dependent kinase (CDK) inhibitors (**III**, **IV**)^17^^,^^18^, anti-inflammation (**V**)^19^ and bumped kinase inhibitors (**VI**, Figure 1)^20^.

**Figure 2.**
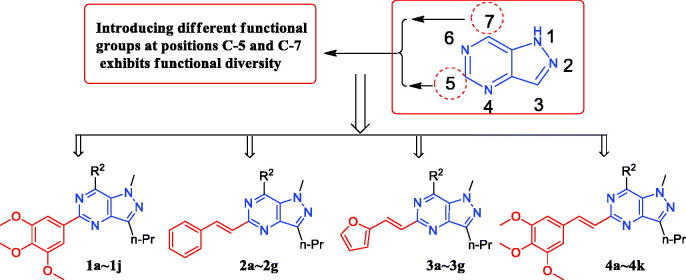
Summary of target derivatives.

In our previous study, a series of pyrazole-pyrimidine derivatives were synthesized for antitumor evalution^21^. Further studies, some analogs exhibited anti-inflammatory activity in RAW 264.7 macrophage cells. In order to find potent anti-inflammatory agents, the scaffold was further modified and evaluated for their inhibitory effect against LPS-induced NO production in RAW264.7 macrophages (Figure 2).

## 2. Experimental section

### 2.1. Chemistry

Commercial reagents were used without further purification. Thin layer chromatography (TLC) was carried out on pre-coated silica GF254 plates with visualization by UV light at 254 nm in the appropriate solvents system for all reactions. Unless noted otherwise, the purification of all compounds was processed by silica gel column chromatography. Melting points were determined on a XT4MP apparatus (Taike Corp., Beijing, China) without correction. ^1^H and ^13^C NMR spectra were recorded on a Bruker AM-300 (^1^H, 300 MHz; ^13^C, 75 MHz) or Agilent DD2 600 MHz (^1^H, 600 MHz; ^13^C, 151 MHz). The ^1^H and ^13^C spectra were recorded in CDCl_3_ or DMSO-*d*_6_ using tetramethylsilane (TMS) as the internal standard. High-resolution electron impact mass spectra (HR-MS) were recorded on a Micro Mass GCT CA 055 instrument under 70 eV electron impact.

### 2.2. General procedures for the synthesis of title compounds 1a–1j, 2a–2g, 3a–3g and 4a–4k

A mixture of intermediate **C1** (1.88 g, 5 mmol) and 4-aminophenol (0.545 g, 5 mmol) were dissolved in isopropanol (IPA) (20 ml). The reaction mixture was refluxed for 6–10 h monitored by TLC. The solvent was removed under reduced pressure, and the residue was purified by column chromatography (gradient elution of PE/EtOAc 85/15 then 80/20 v/v) to obtain compound **1a**.

**Scheme 1.**
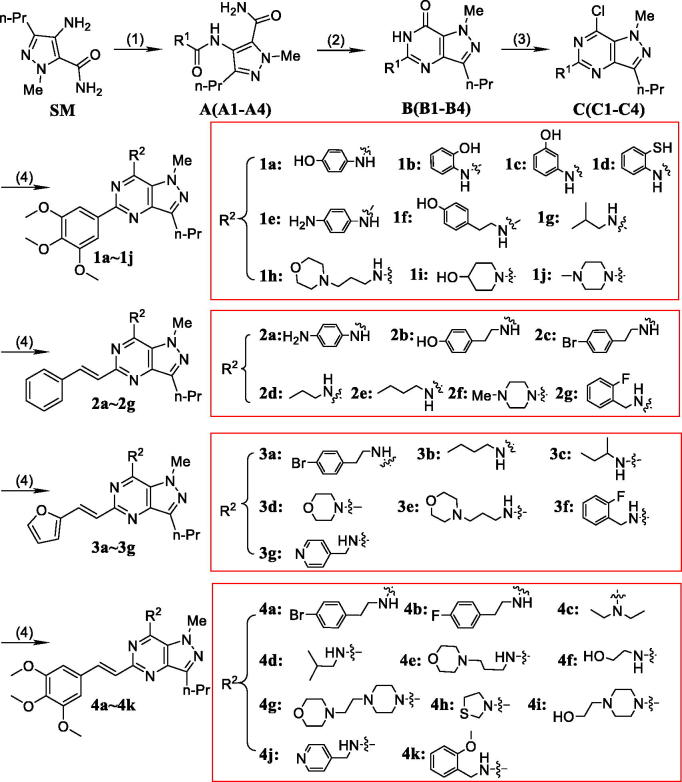
Synthesis of pyrazolo[4,3-*d*]pyrimidine derivatives **1a∼1j**, **2a∼2g**, **3a∼3g** and **4a∼4k**^a^. ^a^Reaction conditions and reagents: (1) substituted carboxylic acid, EDCI, HOBt, TEA, rt, stirred for 18 h; (2) NaOEt, EtOH, reflux, 8**–**10 h; (3) POCl_3_, reflux, 8 h; (4) amine derivatives, IPA, reflux.

Compounds **1 b–1j**, **2a–2g**, **3a–3g** and **4a–4k** were obtained using the same method (Scheme 1).

*4–(1-Methyl-3-propyl-5–(3,4,5-trimethoxyphenyl)-1H-pyrazolo[4,3-d]pyrimidin-7-ylamino)phenol***(1a)**. Title compound **1a** was isolated as a white powder in 79.2% yield (1.78 g, 3.96 mmol), mp 241 – 242 °C. ^1^HNMR (600 MHz, DMSO-*d_6_*): *δ* 9.34 (d, *J* = 3.3 Hz, 1H, NH), 8.70 (brs, 1H, OH), 7.65 (d, *J* = 2.1 Hz, 2H, ArH), 7.58 (d, *J* = 6.7 Hz, 2H, ArH), 6.84 (d, *J* = 6.6 Hz, 2H, ArH), 4.31 (d, *J* = 2.4 Hz, 3H, OCH_3_), 3.84 (d, *J* = 2.1 Hz, 6H, 2 × OCH_3_), 3.72 (d, *J* = 2.2 Hz, 3H, NCH_3_), 2.90 (s, 2H, CH_2_), 1.86 – 1.84 (m, 2H, CH_2_), 0.99 (t, *J* = 7.2 Hz, 3H, CH_3_). ^13^C NMR (151 MHz, DMSO-*d_6_*): *δ* 155.0,154.1, 152.6, 147.6, 144.0, 143.1, 138.7, 133.9, 130.1, 125.1, 120.8, 114.6, 104.6, 60.1, 55.6, 39.2, 27.2, 21.4, 13.9. HR-MS (ESI): calcd for C_24_H_28_N_5_O_4_ [M + H]^+^, 450.2136; found 450.2136.

*2–(1-Methyl-3-propyl-5–(3,4,5-trimethoxyphenyl)-1H-pyrazolo[4,3-d]pyrimidin-7-ylamino)phenol***(1 b).** Title compound **1 b** was isolated as a white powder in 66.7% yield (1.50 g, 3.34 mmol), mp 207 – 208 °C.^1^H NMR (600 MHz, CDCl_3_ + DMSO-*d_6_*): *δ* 7.95 (s, 1H, OH), 7.68 (s, 2H, ArH), 7.12 (t, *J* = 7.5 Hz, 1H, ArH), 7.07 (d, *J* = 8.0 Hz, 2H, ArH), 6.90 (t, *J* = 7.5 Hz, 1H, ArH), 4.43 (s, 3H, OCH_3_), 3.90 (s, 6H, 2 × OCH_3_), 3.83 (s, 3H, NCH_3_), 3.13 (t, *J* = 7.2 Hz, 2H, CH_2_), 1.89 – 1.80 (m, 2H, CH_2_), 1.05 (t, *J* = 7.3 Hz, 3H, CH_3_). HR-MS (ESI): calcd for C_24_H_28_N_5_O_4_ [M + H]^+^, 450.2136; found 450.2134.

*3–(1-Methyl-3-propyl-5–(3,4,5-trimethoxyphenyl)-1H-pyrazolo[4,3-d]pyrimidin-7-ylamino)phenol***(1c)**. Title compound **1c** was isolated as a white powder in 72.0% yield (1.62 g, 3.6 mmol), mp 221 – 222 °C. ^1^H NMR (600 MHz, DMSO-*d_6_*): *δ* 9.76 (s, 1H, OH), 7.74 (s, 2H, ArH), 7.31 (s, 1H, ArH), 7.25 (t, *J* = 8.0 Hz, 1H, ArH), 7.18 (d, *J* = 8.0 Hz, 1H, ArH), 6.71 (d, *J* = 8.0 Hz, 1H, ArH), 4.37 (s, 3H, OCH_3_), 3.87(s, 6H, 2 × OCH_3_), 3.74 (s, 3H, NCH_3_), 3.08 (t, *J* = 7.5 Hz, 2H, CH_2_), 1.84 – 1.75 (m, 2H, CH_2_), 0.99 (t, *J* = 7.3 Hz, 3H, CH_3_). ^13^C NMR (151 MHz, DMSO-*d_6_*): *δ* 157.7, 154.0, 152.8, 148.3, 141.7, 140.3, 138.7, 129.1, 121.3, 114.8, 112.5, 111.3, 107.0, 106.2, 99.3, 60.2, 56.0, 27.4, 25.5, 21.9, 13.8. HR-MS (ESI): calcd for C_24_H_28_N_5_O_4_ [M + H]^+^, 450.2136; found 450.2137.

*2–(1-Methyl-3-propyl-5–(3,4,5-trimethoxyphenyl)-1H-pyrazolo[4,3-d]pyrimidin-7-ylamino)benzenethiol***(1d)**. Title compound **1d** was isolated as a yellow solid in 47.4% yield (1.10 g, 2.37 mmol), mp 269 – 271 °C. ^1^H NMR (600 MHz, DMSO-*d_6_*): *δ* 11.85 (s, 1H), 10.22 (s, 1H, SH), 9.17 (s, 1H, NH), 8.23 (d, *J* = 8.1 Hz, 1H, ArH), 8.15 (d, *J* = 8.2 Hz, 1H, ArH), 7.62 (t, *J* = 7.7 Hz, 1H, ArH), 7.53 (m, 3H, ArH), 4.34 (s, 3H, OCH_3_), 3.96 (s, 6H, 2 × OCH_3_), 3.81 (s, 3H, NCH_3_), 2.57 (m, 2H, CH_2_), 1.71 – 1.67(m, 2H, CH_2_), 0.97 (t, *J* = 7.3 Hz, 3H, CH_3_). ^13^C NMR (151 MHz, DMSO-*d_6_*): *δ* 164.5, 154.1, 153.0, 152.1, 147.8, 142.3, 133.8, 132.0, 127.2, 126.3, 123.3, 122.6, 121.8, 114.7, 106.7, 60.4, 56.5, 40.6, 27.2, 21.4, 14.0. HR-MS (ESI): calcd for C_24_H_28_N_5_O_3_S [M + H]^+^, 466.1907; found 466.1910.

*N1-(1-Methyl-3-propyl-5–(3,4,5-trimethoxyphenyl)-1H-pyrazolo[4,3-d]pyrimidin-7-yl)benzene-1,4-diamine***(1e)**. Title compound **1e** was isolated as a yellow solid in 78.4% yield (1.76 g, 3.92 mmol), mp240 – 241 °C. ^1^H NMR (600 MHz, DMSO-*d_6_*): *δ* 10.42 (brs, 2H, NH_2_), 9.51 (brs, 1H, NH), 7.92 (d, *J* = 7.9 Hz, 2H, ArH), 7.66 (s, 2H, ArH), 7.47 (d, *J* = 7.6 Hz, 2H, ArH), 4.36 (s, 3H, OCH_3_), 3.85 (s, 6H, 2 × OCH_3_), 3.73 (s, 3H, NCH_3_), 2.98 (s, 2H, CH_2_), 1.86 – 1.82 (m, 2H, CH_2_), 0.99 (t, *J* = 7.3 Hz, 3H, CH_3_). HR-MS (ESI): calcd for C_24_H_29_N_6_O_3_ [M + H]^+^, 449.2296; found 449.2302.

*4–(2-(1-Methyl-3-propyl-5–(3,4,5-trimethoxyphenyl)-1H-pyrazolo[4,3-d]pyrimidin-7-ylamino)ethyl)phenol***(1f)**. Title compound **1f** was isolated as a white solid in 64.7% yield (1.54 g, 3.24 mmol), mp 197 – 199 °C. ^1^H NMR (300 MHz, DMSO-*d_6_*): *δ* 9.24 (s, 1H, OH), 7.77 (s, 2H), 7.44 (t, *J* = 5.6 Hz, 1H, NH), 7.13 (d, *J* = 8.3 Hz, 2H), 6.72 (d, *J* = 8.3 Hz, 2H), 4.18 (s, 3H), 3.88 (s, 6H), 3.85 – 3.76 (m, 2H), 3.74 (s, 3H), 2.99 – 2.90 (m, 2H), 2.87 (t, *J* = 7.4 Hz, 2H), 1.89 – 1.77 (m, 2H), 0.98 (t, *J* = 7.4 Hz, 3H). ^13^C NMR (75 MHz, DMSO-*d_6_*): *δ* 155.7, 155.4, 152.6, 149.2, 143.7, 142.1, 138.8, 134.4, 129.6, 129.4, 120.8, 115.1, 104.7, 60.1, 55.7, 42.4, 39.0, 33.9, 27.2, 21.4, 13.9. HR-MS (ESI): calcd for C_26_H_32_N_5_O_4_ [M + H]^+^, 478.2449; found 478.2452.

*N-Isobutyl-1-methyl-3-propyl-5–(3,4,5-trimethoxyphenyl)-1H-pyrazolo[4,3-d]pyrimidin-7-amine***(1 g)**. Title compound **1 g** was isolated as a white solid in 88.3% yield (1.83 g, 4.41 mmol), mp 138 – 139 °C. ^1^H NMR (300 MHz, CDCl_3_): *δ* 7.79 (s, 2H, ArH), 5.24 (t, *J* = 5.5 Hz, 1H, NH), 4.25 (s, 3H, OCH_3_), 3.99 (s, 6H, 2 × OCH_3_), 3.90 (s, 3H, NCH_3_), 3.58 (t, *J* = 6.2 Hz, 2H, NCH_2_), 2.98 (t, *J* = 7.6 Hz, 2H, CH_2_), 2.19 (m, 1H, CH), 1.94 – 1.84 (m, 2H, CH_2_), 1.07 – 1.02 (m, 9H, CH_3_). HR-MS (ESI): calcd for C_22_H_32_N_5_O_3_ [M + H]^+^, 414.2500; found 414.2505.

*1-Methyl-N-(3-morpholinopropyl)-3-propyl-5–(3,4,5-trimethoxyphenyl)-1H-pyrazolo[4,3-d]pyrimidin-7-amine***(1 h)**. Title compound **1 h** was isolated as a white solid in 68.0% yield (1.65 g, 3.40 mmol), mp 167 – 168 °C. ^1^H NMR (600 MHz, CDCl_3_) *δ*: 7.78 (s, 2H, ArH), 6.20 (s, 1H, NH), 4.27 (s, 3H, OCH_3_), 3.99 (s, 6H, 2 × OCH_3_), 3.90 (s, 3H, NCH_3_), 3.84 (dd, *J* = 11.6, 5.9 Hz, 2H, CH_2_), 3.77 – 3.68 (m, 4H, 2 × CH_2_), 2.98 (t, *J* = 7.6 Hz, 2H, CH_2_), 2.59 (dd, *J* = 12.6, 6.4 Hz, 2H, CH_2_), 2.52 (s, 4H, 2 × CH_2_), 2.02 – 1.96 (m, 2H, CH_2_), 1.93 – 1.88 (m, 2H, CH_2_), 1.04 (t, *J* = 7.4 Hz, 3H, CH_3_). ^13^C NMR (151 MHz, CDCl_3_): *δ* 156.9, 153.1, 149.7, 146.0, 143.3, 139.5, 134.9, 121.3, 105.3, 66.7, 61.0, 58.4, 56.2, 54.2, 41.1, 39.4, 27.9, 24.6, 22.2, 14.2. HR-MS (ESI): calcd for C_25_H_37_N_6_O_4_ [M + H]^+^, 485.2871; found 485.2873.

*1–(1-Methyl-3-propyl-5–(3,4,5-trimethoxyphenyl)-1H-pyrazolo[4,3-d]pyrimidin-7-yl)piperidin-4-ol***(1i)**. Title compound **1i** was isolated as a white powder in 55.3% yield (1.22 g, 2.77 mmol), mp 143 – 144 °C. ^1^H NMR (600 MHz, DMSO-*d_6_*): *δ* 7.73 (s, 2H, ArH), 4.84 (s, 1H, OH), 4.07 (s, 3H, OCH_3_), 3.88 (m, 8H, 2 × OCH_3_ + CH_2_), 3.81 (s, 1H, OCH), 3.74 (s, 3H, NCH_3_), 3.30 (s, 2H, CH_2_), 2.91 (d, *J* = 3.1 Hz, 2H, CH_2_), 1.95 (s, 2H, CH_2_), 1.85 (s, 2H, NCH_2_), 1.63 (d, *J* = 8.2 Hz, 2H, NCH_2_), 1.02 – 0.96 (m, 3H, CH_3_). ^13^C NMR (151 MHz, DMSO-*d_6_*): *δ* 154.9, 153.4, 152.8, 145.3, 144.2, 139.1, 133.7, 123.6, 104.8, 65.6, 60.1, 55.8, 46.7, 38.7, 33.7, 27.3, 21.3, 14.0. HR-MS (ESI): calcd for C_23_H_32_N_5_O_4_ [M + H]^+^, 442.2449; found 442.2447.

*1-Methyl-7–(4-methylpiperazin-1-yl)-3-propyl-5–(3,4,5-trimethoxyphenyl)-1H-pyrazolo[4,3-d]pyrimidine***(1j)**. Title compound **1j** was isolated as a white solid in 82.9% yield (1.83 g, 4.15 mmol), mp 116 – 117 °C. ^1^H NMR (600 MHz, CDCl_3_): *δ* 7.79 (s, 2H, ArH), 4.11 (s, 3H, OCH_3_), 3.99 (s, 6H, 2 × OCH_3_), 3.91 (s, 3H, NCH_3_), 3.65 (s, 4H, 2 × NCH_2_), 3.03 (t, *J* = 7.6 Hz, 2H, CH_2_), 2.69 (s, 4H, 2 × NCH_2_), 2.41 (s, 3H, NCH_3_), 1.94 – 1.91 (m, 2H, CH_2_), 1.06 (t, *J* = 7.4 Hz, 3H, CH_3_). ^13^C NMR (151 MHz, CDCl_3_): *δ* 156.4, 153.9, 153.2, 147.6, 145.6, 139.8, 134.3, 124.5, 105.4, 61.0, 56.3, 54.6, 49.4, 46.3, 38.6, 28.0, 22.2, 14.2. HR-MS (ESI): calcd for C_23_H_33_N_6_O_3_ [M + H]^+^, 441.2609; found 441.2613.

*(E)-N1-(1-Methyl-3-propyl-5-styryl-1H-pyrazolo[4,3-d]pyrimidin-7-yl)benzene-1,4-diamine***(2a)**. Title compound **2a** was isolated as a white solid in 54.6% yield (1.05 g, 2.73 mmol), mp 163 – 164 °C. ^1^H NMR (300 MHz, DMSO-*d_6_*): *δ* 9.40 (s, 2H), 7.90 – 7.78 (m, 2H, ArH), 7.73 – 7.58 (m, 3H, ArH), 7.46 – 7.26 (m, 6H, ArH), 4.34 (s, 3H, NCH_3_), 2.94 – 2.87 (m, 2H, CH_2_), 1.86 – 1.69 (m, 2H, CH_2_), 1.02 – 0.92 (m, 3H, CH_3_). HR-MS (ESI): calcd for C_23_H_25_N_6_ [M + H]^+^, 385.2135; found 385.2131.

*(E)-4–(2-(1-Methyl-3-propyl-5-styryl-1H-pyrazolo[4,3-d]pyrimidin-7-ylamino)ethyl)phenol***(2 b)**. Title compound **2 b** was isolated as a white solid in 73.2% yield (1.51 g, 3.66 mmol), mp 163 – 164 °C. ^1^H NMR (300 MHz, DMSO-*d_6_*): *δ* 9.24 (s, 1H), 7.81 (d, *J* = 15.9 Hz, 1H), 7.67 (d, *J* = 7.3 Hz, 2H), 7.43 (t, *J* = 7.4 Hz, 2H), 7.33 (t, *J* = 7.0 Hz, 2H), 7.16 (dd, *J* = 12.1, 3.7 Hz, 3H), 6.75 (d, *J* = 8.3 Hz, 2H), 4.16 (s, 3H), 3.76 (dd, *J* = 14.7, 5.8 Hz, 2H), 2.99 – 2.86 (m, 2H), 2.80 (t, *J* = 7.5 Hz, 2H), 1.83 – 1.70 (m, 2H), 0.95 (t, *J* = 7.4 Hz, 3H). ^13^C NMR (75 MHz, CDCl_3_): *δ* 156.6, 155.7, 149.0, 143.8, 141.9, 136.3, 134.2, 129.7, 129.6, 129.3, 128.9, 128.3, 127.1, 120.8, 115.2, 42.6, 39.0, 33.87, 27.3, 21.7, 13.9. HR-MS (ESI): calcd for C_25_H_28_N_5_O [M + H]^+^, 414.2288; found 414.2291.

*(E)-N-(4-Bromophenethyl)-1-methyl-3-propyl-5-styryl-1H-pyrazolo[4,3-d]pyrimidin-7-amine***(2c)**. Title compound **2c** was isolated as a yellow solid in 81.1% yield (1.93 g, 4.06 mmol), mp 209 – 210 °C. ^1^H NMR (600 MHz, CDCl_3_): *δ* 7.88 (d, *J* = 15.8 Hz, 1H), 7.62 (d, *J* = 7.5 Hz, 2H), 7.48 (d, *J* = 8.2 Hz, 2H), 7.38 (t, *J* = 7.6 Hz, 2H), 7.30 (t, *J* = 7.3 Hz, 1H), 7.23 (d, *J* = 15.8 Hz, 1H), 7.18 (d, *J* = 8.1 Hz, 2H), 5.05 (s, 1H), 4.07 (s, 3H), 3.98 (dd, *J* = 12.7, 6.7 Hz, 2H), 3.06 (t, *J* = 6.9 Hz, 2H), 2.94 (t, *J* = 7.7 Hz, 2H), 1.90 – 1.82 (m, 2H), 1.03 (t, *J* = 7.3 Hz, 3H). ^13^C NMR (151 MHz, CDCl_3_): *δ* 157.7, 149.0, 145.7, 143.1, 137.9, 136.8, 135.4, 131.9, 130.6, 129.0, 128.7, 128.2, 127.3, 121.0, 120.6, 41.8, 38.9, 34.7, 27.7, 22.3, 14.1. HR-MS (ESI): calcd for C_25_H_27_N_5_Br [M + H]^+^, 476.1444; found 476.1448.

*(E)-1-Methyl-N,3-dipropyl-5-styryl-1H-pyrazolo[4,3-d]pyrimidin-7-amine***(2d)**. Title compound **2d** was isolated as a white solid in 84.3% yield (1.41 g, 4.22 mmol), mp 139 – 140 °C. ^1^H NMR (600 MHz, CDCl_3_ + DMSO-*d_6_*): *δ* 9.03 (s, 1H, NH), 8.04 (d, *J* = 15.6 Hz, 1H, ArH), 7.72 (d, *J* = 15.6 Hz, 1H, ArH), 7.67 (d, *J* = 3.8 Hz, 2H, ArH), 7.43 (s, 3H, ArH), 4.42 (s, 3H, NCH_3_), 3.86 (dd, *J* = 13.4, 6.6 Hz, 2H, NCH_2_), 3.07 (t, *J* = 7.5 Hz, 2H, CH_2_), 1.89 (dt, *J* = 14.6, 7.3 Hz, 2H, CH_2_), 1.86 – 1.77 (m, 2H, CH_2_), 1.08 (t, *J* = 7.4 Hz, 3H, CH_3_), 1.04 (t, *J* = 7.3 Hz, 3H, CH_3_). ^13^C NMR (151 MHz, CDCl_3_ + DMSO-*d_6_*): *δ* 152.8, 149.8, 142.4, 139.5, 133.8, 129.8, 128.3, 128.3, 127.7, 120.3, 118.2, 43.1, 39.6, 27.0, 21.7, 21.4, 13.1, 11.0. HR-MS (ESI): calcd for C_20_H_26_N_5_ [M + H]^+^, 336.2183; found 336.2184.

*(E)-N-Butyl-1-methyl-3-propyl-5-styryl-1H-pyrazolo[4,3-d]pyrimidin-7-amine***(2e)**. Title compound **2e** was isolated as a white solid in 91.9% yield (1.61 g, 4.60 mmol), mp 156 – 157 °C. ^1^H NMR (300 MHz, DMSO-*d_6_*): *δ* 8.71 (brs, 1H, NH), 8.06 (d, *J* = 15.7 Hz, 1H, =CH), 7.70 (d, *J* = 6.6 Hz, 2H, ArH), 7.59 – 7.42 (m, 4H, ArH), 4.28 (s, 3H, NCH_3_), 3.81 (dd, *J* = 13.2, 6.4 Hz, 2H, CH_2_), 2.92 (t, *J* = 7.5 Hz, 2H, CH_2_), 1.76 – 1.67 (m, 4H, 2 × CH_2_), 1.50 – 1.42 (m, 2H, CH_2_), 1.01 – 0.93 (m, 6H, 2 × CH_3_). HR-MS (ESI): calcd for C_21_H_28_N_5_ [M + H]^+^, 350.2339; found 350.2338.

*(E)-1-Methyl-7–(4-methylpiperazin-1-yl)-3-propyl-5-styryl-1H-pyrazolo[4,3-d]pyrimidine***(2f).** Title compound **2f** was isolated as a white solid in 56.8% yield (1.07 g, 2.84 mmol), mp 156 – 157 °C. ^1^H NMR (300 MHz, DMSO-*d_6_*): *δ* 11.86 (brs, 1H), 8.02 (d, *J* = 15.8 Hz, 1H), 7.74 (d, *J* = 7.1 Hz, 2H), 7.47 (dt, *J* = 17.9, 9.1 Hz, 4H), 4.42 (d, *J* = 13.1 Hz, 2H), 4.14 (s, 3H), 3.79 (t, 2H), 3.55 (d, *J* = 12.0 Hz, 2H), 3.30 (d, *J* = 9.7 Hz, 2H), 2.96 (t, *J* = 7.4 Hz, 2H), 2.83 (s, 3H), 1.80 – 1.73 (m, 2H), 0.98 (t, *J* = 7.3 Hz, 1H). HR-MS (ESI): calcd for C_22_H_29_N_6_ [M + H]^+^, 377.2448; found 377.2447.

*(E)-N-(2-Fluorobenzyl)-1-methyl-3-propyl-5-styryl-1H-pyrazolo[4,3-d]pyrimidin-7-amine***(2 g)**. Title compound **2 g** was isolated as a yellow solid in 77.9% yield (1.56 g, 3.90 mmol), mp 175 – 176 °C. ^1^H NMR (600 MHz, CDCl_3_): *δ* 7.88 (d, *J* = 15.8 Hz, 1H), 7.62 (d, *J* = 7.7 Hz, 2H), 7.55 (t, *J* = 7.5 Hz, 1H), 7.38 (t, *J* = 7.6 Hz, 2H), 7.29 (dd, *J* = 12.1, 6.3 Hz, 2H), 7.22 (d, *J* = 15.8 Hz, 1H), 7.16 – 7.08 (m, 2H), 5.52 (s, 1H, NH), 5.01 (d, *J* = 5.7 Hz, 2H, CH_2_), 4.22 (s, 3H), 2.94 (t, *J* = 7.7 Hz, 2H), 1.87 – 1.83 (m, 2H), 1.02 (t, *J* = 7.3 Hz, 3H). ^13^C NMR (151 MHz, CDCl_3_): *δ* 161.4 (d, *J* = 245.5 Hz), 157.6, 149.0, 145.7, 143.2, 136.9, 135.5, 130.6 (d, *J* = 4.4 Hz), 129.5 (d, *J* = 8.3 Hz), 128.9, 128.6, 128.2, 127.3, 125.5 (d, *J* = 14.2 Hz), 124.4 (d, *J* = 3.5 Hz), 121.1, 115.5 (d, *J* = 21.4 Hz), 39.0, 39.0, 27.7, 22.2, 14.0. HR-MS (ESI): calcd for C_24_H_25_N_5_F [M + H]^+^, 402.2089; found 402.2084.

*(E)-N-(4-Bromophenethyl)-5–(2-(furan-2-yl)vinyl)-1-methyl-3-propyl-1H-pyrazolo[4,3-d]pyrimidin-7-amine***(3a)**. Title compound **3a** was isolated as a yellow solid in 66.6% yield (1.55 g, 3.33 mmol), mp 128 – 129 °C. ^1^H NMR (600 MHz, CDCl_3_): *δ* 7.65 (d, *J* = 15.7 Hz, 1H), 7.47 (d, *J* = 8.2 Hz, 2H), 7.45 (s, 1H), 7.15 (d, *J* = 8.2 Hz, 2H), 7.12 (d, *J* = 15.7 Hz, 1H), 6.50 (d, *J* = 3.2 Hz, 1H), 6.45 (dd, *J* = 3.0, 1.7 Hz, 1H), 5.03 (s, 1H), 4.05 (s, 3H), 3.94 (q, *J* = 6.7 Hz, 2H), 3.03 (t, *J* = 6.9 Hz, 2H), 2.95 – 2.90 (m, 2H), 1.86 – 1.82(m, 2H), 1.01 (t, *J* = 7.3 Hz, 3H). ^13^C NMR (151 MHz, CDCl_3_); *δ* 157.5, 153.2, 149.0, 145.7, 143.1, 142.9, 138.0, 131.8, 130.5, 127.3, 122.9, 121.0, 120.6, 111.7, 110.1, 41.8, 38.8, 34.7, 27.7, 22.2, 14.0. HR-MS (ESI): calcd for C_23_H_25_BrN_5_O [M + H]^+^, 466.1237; found 466.1238.

*(E)-N-Butyl-5–(2-(furan-2-yl)vinyl)-1-methyl-3-propyl-1H-pyrazolo[4,3-d]pyrimidin-7-amine***(3 b)**. Title compound **3 b** was isolated as a yellow solid in 87.4% yield (1.48 g, 4.37 mmol), mp 101 – 102 °C. ^1^H NMR (600 MHz, CDCl_3_): *δ* 7.61 (d, *J* = 15.7 Hz, 1H), 7.42 (s, 1H), 7.09 (d, *J* = 15.7 Hz, 1H), 6.47 (d, *J* = 3.2 Hz, 1H), 6.42 (dd, *J* = 3.1, 1.7 Hz, 1H), 5.07 (s, 1H), 4.18 (s, 3H), 3.68 (q, *J* = 7.0 Hz, 2H), 2.94 – 2.87 (m, 2H), 1.85 – 1.81 (m, 2H), 1.74 – 1.67 (m, 2H), 1.49 – 1.45(m, 2H), 1.00 (t, *J* = 7.3 Hz, 6H). ^13^C NMR (151 MHz, CDCl_3_): *δ* 157.7, 153.4, 149.5, 145.6, 143.0, 142.9, 127.5, 122.9, 121.2, 111.8, 110.2, 40.7, 39.1, 31.6, 27.8, 22.3, 20.4, 14.2, 14.0. HR-MS (ESI): calcd for C_19_H_26_N_5_O [M + H]^+^, 340.2132; found 340.2137.

*(E)-N-sec-Butyl-5–(2-(furan-2-yl)vinyl)-1-methyl-3-propyl-1H-pyrazolo[4,3-d]pyrimidin-7-amine***(3c)**. Title compound **3c** was isolated as a white solid in 89.4% yield (1.51 g, 4.47 mmol), mp 115 – 116 °C. ^1^H NMR (600 MHz, CDCl_3_): *δ* 7.60 (d, *J* = 15.7 Hz, 1H), 7.43 (s, 1H), 7.10 (d, *J* = 15.7 Hz, 1H), 6.49 (d, *J* = 3.1 Hz, 1H), 6.43 (dd, *J* = 3.2, 1.7 Hz, 1H), 4.80 (d, *J* = 7.5 Hz, 1H), 4.52 – 4.45 (m, 1H), 4.21 (s, 3H), 2.95 – 2.90 (m, 2H), 1.88 – 1.82 (m, 2H), 1.77 – 1.63 (m, 2H), 1.34 (d, *J* = 6.5 Hz, 3H), 1.05 – 1.02 (m, 3H), 1.01 (t, *J* = 5.8 Hz, 3H). ^13^C NMR (151 MHz, CDCl_3_): *δ* 157.8, 153.4, 149.1, 145.7, 143.1, 142.9, 127.7, 122.9, 121.1, 111.8, 110.1, 47.8, 39.1, 29.7, 27.8, 22.4, 20.4, 14.2, 10.6. HR-MS (ESI): calcd for C_19_H_26_N_5_O [M + H]^+^, 340.2132; found 340.2126.

*(E)-4–(5–(2-(Furan-2-yl)vinyl)-1-methyl-3-propyl-1H-pyrazolo[4,3-d]pyrimidin-7-yl)morpholine***(3d)**. Title compound **3d** was isolated as a white solid in 73.9% yield (1.31 g, 3.70 mmol), mp 108 – 109 °C. ^1^H NMR (600 MHz, CDCl_3_): *δ* 7.63 (d, *J* = 15.7 Hz, 1H), 7.44 (s, 1H), 7.15 (d, *J* = 15.7 Hz, 1H), 6.50 (d, *J* = 3.2 Hz, 1H), 6.44 (dd, *J* = 3.2, 1.7 Hz, 1H), 4.08 (s, 3H), 3.94 – 3.90 (m, 4H), 3.57 – 3.54 (m, 4H), 2.96 (t, 2H), 1.89 – 1.85 (m, 2H), 1.02 (t, *J* = 7.4 Hz, 3H). ^13^C NMR (151 MHz, CDCl_3_): *δ* 157.0, 153.5, 153.0, 147.4, 145.4, 142.9, 126.8, 124.2, 123.1, 111.8, 110.4, 66.4, 49.9, 38.4, 27.8, 22.1, 14.0. HR-MS (ESI): calcd for C_19_H_24_N_5_O_2_ [M + H]^+^, 354.1925; found 354.1925.

*(E)-5–(2-(Furan-2-yl)vinyl)-1-methyl-N-(3-morpholinopropyl)-3-propyl-1H-pyrazolo[4,3-d]pyrimidin-7-amine***(3e)**. Title compound **3e** was isolated as a white solid in 56.4% yield (1.16 g, 2.82 mmol), mp 98 – 99 °C. ^1^H NMR (600 MHz, CDCl_3_): *δ* 7.62 (d, *J* = 15.7 Hz, 1H), 7.43 (s, 1H), 7.10 (d, *J* = 15.7 Hz, 1H), 6.48 (d, *J* = 3.1 Hz, 1H), 6.43 (dd, *J* = 3.1, 1.7 Hz, 1H), 6.07 (s, 1H), 4.24 (s, 3H), 3.80 (q, *J* = 6.1 Hz, 2H), 3.72 (t, *J* = 4.4 Hz, 4H), 2.94 – 2.91 (m, 2H), 2.57 (t, *J* = 6.2 Hz, 2H), 2.52 (s, 4H), 1.97 – 1.89 (m, 2H), 1.87 – 1.83 (m, 2H), 1.01 (t, *J* = 7.4 Hz, 3H). ^13^C NMR (151 MHz, CDCl_3_): *δ* 157.7, 153.4, 149.5, 145.8, 143.0, 142.9, 127.7, 122.8, 121.3, 111.9, 110.1, 66.8, 58.4, 54.2, 40.9, 39.3, 27.9 24.7 22.4 14.2. HR-MS (ESI): calcd for C_22_H_31_N_6_O_2_ [M + H]^+^, 411.2503; found 411.2510.

*(E)-N-(2-Fluorobenzyl)-5–(2-(furan-2-yl)vinyl)-1-methyl-3-propyl-1H-pyrazolo[4,3-d]pyrimidin-7-amine***(3f)**. Title compound **3f** was isolated as a white solid in 79.4% yield (1.55 g, 3.97 mmol), mp132 – 133 °C. ^1^H NMR (300 MHz, DMSO-*d_6_*): *δ* 7.87 (t, *J* = 5.6 Hz, 1H, ArH), 7.73 (s, 1H, NH), 7.52 (t, *J* = 7.6 Hz, 1H, ArH), 7.43 (d, *J* = 15.8 Hz, 1H, =CH), 7.33 – 7.18 (m, 2H, ArH), 7.13 (t, *J* = 7.2 Hz, 1H, ArH), 6.79 (d, *J* = 15.8 Hz, 1H, =CH), 6.69 (d, *J* = 3.3 Hz, 1H, ArH), 6.60 – 6.54 (m, 1H, ArH), 4.84 (d, *J* = 5.5 Hz, 2H, CH_2_), 4.24 (s, 3H, NCH_3_), 2.78 (t, *J* = 7.5 Hz, 2H, CH_2_), 1.78 – 1.71 (m, 2H, CH_2_), 0.93 (t, *J* = 7.4 Hz, 3H, CH_3_). HR-MS (ESI): calcd for C_22_H_23_FN_5_O [M + H]^+^, 392.1881; found 392.1885.

*(E)-5-(2-(Furan-2-yl)vinyl)-1-methyl-3-propyl-N-(pyridin-4-ylmethyl)-1H-pyrazolo[4,3-d]pyrimidin-7-amine***(3 g)**. Title compound **3 g** was isolated as a yellow solid in 82.2% yield (1.54 g, 4.11 mmol), mp 117 – 118 °C. ^1^H NMR (600 MHz, CDCl_3_): *δ* 8.60 – 8.55 (m, 2H), 7.47 (d, *J* = 15.7 Hz, 1H), 7.41 (d, *J* = 1.7 Hz, 1H), 7.34 – 7.32 (m, 2H), 7.07 (d, *J* = 15.7 Hz, 1H), 6.44 (d, *J* = 3.3 Hz, 1H), 6.42 (dd, *J* = 3.3, 1.8 Hz, 1H), 5.56 (t, *J* = 5.8 Hz, 1H), 4.94 (d, *J* = 5.7 Hz, 2H), 4.24 (s, 3H), 2.93 (t, *J* = 7.7 Hz, 2H), 1.87 – 1.83 (m, 2H), 1.01 (t, *J* = 7.3 Hz, 3H). ^13^C NMR (151 MHz, CDCl_3_): *δ* 157.6, 153.1, 150.2, 149.0, 148.1, 146.0, 143.7, 143.1, 127.0, 123.2, 122.6, 121.0, 111.9, 110.5, 43.8, 39.2, 27.9, 22.4, 14.2. HR-MS (ESI): calcd for C_21_H_23_N_6_O [M + H]^+^, 375.1928; found 375.1928.

*(E)-N-(4-Bromophenethyl)-1-methyl-3-propyl-5–(3,4,5-trimethoxystyryl)-1H-pyrazolo[4,3-d]pyrimidin-7-amine***(4a)**. Title compound **4a** was isolated as a yellow solid in 87.2% yield (2.47 g, 4.36 mmol), mp 215 – 217 °C. ^1^H NMR (600 MHz, CDCl_3_): *δ* 7.79 (d, *J* = 15.7 Hz, 1H), 7.48 (d, *J* = 8.4 Hz, 2H), 7.21 – 7.17 (m, 2H), 7.15 (d, *J* = 15.7 Hz, 1H), 6.85 (s, 2H), 5.08 (t, *J* = 5.7 Hz, 1H), 4.08 (s, 3H), 4.03 – 3.96 (m, 2H), 3.92 (s, 6H), 3.89 (s, 3H), 3.07 (t, *J* = 6.9 Hz, 2H), 2.96 – 2.87 (m, 2H), 1.88 – 1.84 (m, 2H), 1.02 (t, *J* = 7.4 Hz, 3H). ^13^C NMR (151 MHz, CDCl_3_): *δ* 157.8, 153.5, 149.2, 145.7, 143.2, 138.5, 138.1, 135.4, 132.6, 132.0, 130.7, 128.6, 121.2, 120.8, 104.4, 61.1, 56.2, 41.9, 39.0, 34.8, 27.8, 22.4, 14.2. HR-MS (ESI): calcd for C_28_H_32_BrN_5_O_3_ [M + H]^+^, 566.1761; found 566.1760.

*(E)-N-(4-Fluorophenethyl)-1-methyl-3-propyl-5–(3,4,5-trimethoxystyryl)-1H-pyrazolo[4,3-d]pyrimidin-7-amine***(4b)**. Title compound **4b** was isolated as a yellow solid in 67.7% yield (1.71 g, 3.39 mmol), mp 186 – 187 °C. ^1^H NMR (300 MHz, CDCl_3_): *δ* 7.80 (d, *J* = 15.7 Hz, 1H), 7.31 – 7.23 (m, 2H), 7.15 (d, *J* = 15.7 Hz, 1H), 7.05 (t, *J* = 8.6 Hz, 2H), 6.86 (s, 2H), 5.07 (t, *J* = 5.6 Hz, 1H), 4.06 (s, 3H), 3.99 (t, *J* = 6.4 Hz, 2H), 3.92 (s, 6H), 3.89 (s, 3H), 3.08 (t, *J* = 6.8 Hz, 2H), 2.94 (t, *J* = 7.7 Hz, 2H), 1.89 – 1.82 (m, 2H),1.02 (t, *J* = 7.4 Hz, 3H). ^13^C NMR (151 MHz, CDCl_3_): *δ* 161.9 (d, *J* = 245.4 Hz), 157.8, 153.5, 149.3, 145.8, 143.2, 138.6, 135.4, 134.7 (d, *J* = 3.4 Hz), 132.7, 130.4 (d, *J* = 7.9 Hz), 128.7, 121.2, 115.8 (d, *J* = 21.3 Hz), 104.4, 61.1, 56.3, 42.1, 39.0, 34.6, 27.8, 22.4, 14.2. HR-MS (ESI): calcd for C_28_H_32_FN_5_O_3_ [M + H]^+^, 506.2562; found 506.2560.

*(E)-N,N-Diethyl-1-methyl-3-propyl-5–(3,4,5-trimethoxystyryl)-1H-pyrazolo[4,3-d]pyrimidin-7-amine***(4c)**. Title compound **4c** was isolated as a yellow solid in 81.3% yield (1.79 g, 4.07 mmol), mp 140 – 141 °C. ^1^H NMR (600 MHz, CDCl_3_): *δ* 7.75 (d, *J* = 15.7 Hz, 1H), 7.16 (d, *J* = 15.7 Hz, 1H), 6.85 (s, 2H), 4.08 (s, 3H), 3.92 (s, 6H), 3.88 (s, 3H), 3.62 (q, *J* = 7.1 Hz, 4H), 2.99 – 2.96 (m, 2H), 1.91 – 1.85 (m, 2H), 1.26 (t, *J* = 7.1 Hz, 6H), 1.04 (t, *J* = 7.4 Hz, 3H). ^13^C NMR (151 MHz, CDCl_3_): *δ* 157.0, 153.6, 153.4, 147.2, 145.1, 138.5, 135.3, 132.7, 128.5, 125.1, 104.5, 61.1, 56.3, 43.9, 38.9, 28.0, 22.3, 14.2, 12.6. HR-MS (ESI): calcd for C_24_H_33_N_5_O_3_ [M + H]^+^, 440.2656; found 440.2657.

*(E)-N-Isobutyl-1-methyl-3-propyl-5–(3,4,5-trimethoxystyryl)-1H-pyrazolo[4,3-d]pyrimidin-7-amine***(4d)**. Title compound **4d** was isolated as a yellow solid in 92.3% yield (2.03 g, 4.62 mmol), mp 171 – 172 °C. ^1^H NMR (600 MHz, CDCl_3_): *δ* 7.75 (d, *J* = 15.7 Hz, 1H, =CH), 7.12 (d, *J* = 15.7 Hz, 1H, =CH), 6.85 (s, 2H, ArH), 5.15 (s, 1H, NH), 4.24 (s, 3H, OCH_3_), 3.92 (s, 6H, 2 × OCH_3_), 3.88 (s, 3H, NCH_3_), 3.59 (t, *J* = 6.1 Hz, 2H, NCH_2_), 2.94 (t, *J* = 7.7 Hz, 2H, CH_2_), 2.12 – 2.08 (m, 1H, CH), 1.91 – 1.82 (m, 2H, CH_2_), 1.08 (d, *J* = 6.7 Hz, 6H, 2 × CH_3_), 1.03 (t, *J* = 7.3 Hz, 3H, CH_3_). ^13^C NMR (151 MHz, DMSO-*d_6_*): *δ* 157.8, 153.4, 149.8, 145.6, 143.0, 138.4, 135.2, 132.7, 128.7, 121.2, 104.4, 61.1, 56.2, 48.3, 39.1, 28.4, 27.8, 22.4, 20.6, 14.1. HR-MS (ESI): calcd for C_24_H_34_N_5_O_3_ [M + H]^+^, 440.2656; found 440.2651.

*(E)-1-Methyl-N-(3-morpholinopropyl)-3-propyl-5–(3,4,5-trimethoxystyryl)-1H-pyrazolo[4,3-d]pyrimidin-7-amine***(4e)**. Title compound **4e** was isolated as a white solid in 62.8% yield (1.74 g, 3.42 mmol), mp 144 – 145 °C. ^1^H NMR (600 MHz, CDCl_3_): *δ* 7.75 (d, *J* = 15.7 Hz, 1H), 7.13 (d, *J* = 15.6 Hz, 1H), 6.85 (s, 2H), 6.18 (t, *J* = 5.0 Hz, 1H), 4.28 (s, 3H), 3.92 (s, 6H), 3.88 (s, 3H), 3.87 – 3.84 (m, 2H), 3.73 (t, *J* = 4.7 Hz, 4H), 2.97 – 2.92 (m, 2H), 2.61 (t, *J* = 6.2 Hz, 2H), 2.54 (s, 4H), 1.97 (t, *J* = 6.3 Hz, 2H), 1.88 – 1.84(m, 2H), 1.03 (t, *J* = 7.3 Hz, 3H). ^13^C NMR (151 MHz, CDCl_3_): *δ* 157.8, 153.4, 149.6, 145.8, 142.9, 138.4, 135.2, 132.7, 128.8, 121.4, 104.4, 66.8, 61.1, 58.6, 56.2, 54.3, 41.2, 39.4, 27.9, 24.6, 22.4, 14.2. HR-MS (ESI): calcd for C_27_H_38_N_6_O_4_ [M + H]^+^, 511.3027; found 511.3028.

*(E)-2–(1-Methyl-3-propyl-5–(3,4,5-trimethoxystyryl)-1H-pyrazolo[4,3-d]pyrimidin-7-ylamino)ethanol***(4f)**. Title compound **4f** was isolated as a white solid in 77.3% yield (1.65 g, 3.87 mmol), mp 127 – 128 °C. ^1^H NMR (600 MHz, DMSO-*d_6_*): *δ* 7.66 (d, *J* = 15.8 Hz, 1H), 7.10 (d, *J* = 15.8 Hz, 1H), 7.06 (s, 1H), 6.97 (s, 2H), 4.83 (t, *J* = 5.1 Hz, 1H), 4.18 (s, 3H), 3.86 (s, 6H), 3.72 (t, *J* = 7.3 Hz, 4H), 3.69 (s, 3H), 2.78 (t, *J* = 7.5 Hz, 2H), 1.77 – 1.73 (m, 2H), 0.94 (t, *J* = 7.4 Hz, 3H). ^13^C NMR (151 MHz, CDCl_3_ + DMSO-*d*): *δ* 156.6, 153.1, 149.3, 143.6, 141.9, 137.7, 134.3, 132.1, 128.7, 120.8, 104.4, 60.0, 59.3, 55.9, 42.9, 39.0, 27.3, 21.8, 13.9. HR-MS (ESI): calcd for C_22_H_30_N_5_O_4_ [M + H]^+^, 42.2292; found 428.2293.

*(E)-4–(2-(4–(1-Methyl-3-propyl-5–(3,4,5-trimethoxystyryl)-1H-pyrazolo[4,3-d]pyrimidin-7-yl)piperazin-1-yl)ethyl)morpholine***(4 g)**. Title compound **4 g** was isolated as a white solid in 54.7% yield (1.55 g, 2.74 mmol), mp 167 – 169 °C. ^1^H NMR (300 MHz, CDCl_3_): *δ* 7.77 (d, *J* = 15.7 Hz, 1H), 7.16 (d, *J* = 15.7 Hz, 1H), 6.85 (s, 2H), 4.09 (s, 3H), 3.92 (s, 6H), 3.88 (s, 3H), 3.73 (t, *J* = 4.7 Hz, 4H), 3.60 (s, 4H), 2.98 (t, *J* = 7.7 Hz, 2H), 2.74 (s, 4H), 2.64 – 2.56 (m, 4H), 2.52 (t, *J* = 4.5 Hz, 4H), 1.91 – 1.83 (m, 2H), 1.03 (t, *J* = 7.3 Hz, 3H). ^13^C NMR (151 MHz, CDCl_3_): *δ* 157.2, 153.8, 153.4, 147.3, 145.0, 138.5, 135.7, 132.5, 128.1, 124.5, 104.4, 67.0, 61.1, 56.4, 56.2, 55.8, 54.3, 53.2, 49.5, 38.7, 28.0, 22.3, 14.2. HR-MS (ESI): calcd for C_30_H_43_N_7_O_4_ [M + H]^+^, 566.3449; found 566.3449.

*(E)-3–(1-Methyl-3-propyl-5–(3,4,5-trimethoxystyryl)-1H-pyrazolo[4,3-d]pyrimidin-7-yl)thiazolidine***(4 h)**. Title compound **4 h** was isolated as a yellow solid in 68.2% yield (1.55 g, 3.41 mmol), mp 145 – 146 °C. ^1^H NMR (600 MHz, CDCl_3_): *δ* 7.74 (d, *J* = 15.7 Hz, 1H), 7.16 (d, *J* = 15.7 Hz, 1H), 6.85 (s, 2H), 4.86 (s, 2H), 4.19 (s, 3H), 4.12 (t, *J* = 6.3 Hz, 2H), 3.93 (s, 6H), 3.89 (s, 3H), 3.21 (t, *J* = 6.3 Hz, 2H), 3.02 – 2.97 (m, 2H), 1.90 – 1.87 (m, 2H), 1.04 (t, *J* = 7.4 Hz, 3H). ^13^C NMR (151 MHz, CDCl_3_): *δ* 156.8, 153.4, 151.8, 146.9, 145.6, 138.7, 135.7, 132.3, 127.8, 124.0, 104.5, 60.9, 60.9, 56.2, 56.2, 55.1, 52.8, 38.9, 38.9, 30.6, 27.8, 22.1, 14.0. HR-MS (ESI): calcd for C_23_H_30_N_5_O_3_S [M + H]^+^, 456.2064; found 456.2065.

*(E)-2–(4-(1-Methyl-3-propyl-5–(3,4,5-trimethoxystyryl)-1H-pyrazolo[4,3-d]pyrimidin-7-yl)piperazin-1-yl)ethanol***(4i)**. Title compound **4i** was isolated as a yellow solid in 75.4% yield (1.87 g, 3.77 mmol), mp 141 – 142 °C. ^1^H NMR (600 MHz, CDCl_3_): *δ* 7.78 (d, *J* = 15.7 Hz, 1H), 7.17 (d, *J* = 15.7 Hz, 1H), 6.86 (s, 2H), 4.11 (s, 3H), 3.93 (s, 6H), 3.89 (s, 3H), 3.70 (t, *J* = 4.8 Hz, 2H), 3.62 (s, 4H), 2.99 (t, *J* = 7.7 Hz, 2H), 2.79 (s, 4H), 2.68 (t, *J* = 4.8 Hz, 2H), 1.90 – 1.86 (m, 2H), 1.04 (t, *J* = 7.4 Hz, 3H). ^13^C NMR (151 MHz, CDCl_3_): *δ* 157.2, 153.8, 153.4, 147.3, 145.1, 138.5, 135.7, 132.5, 128.0, 124.4, 104.4, 61.1, 59.5, 57.9, 56.2, 52.5, 49.6, 38.6, 27.9, 22.3, 14.2. HR-MS (ESI): calcd for C_26_H_37_N_6_O_4_ [M + H]^+^, 492.2871; found 492.2871.

*(E)-1-Methyl-3-propyl-N-(pyridin-4-ylmethyl)-5–(3,4,5-trimethoxystyryl)-1H-pyrazolo[4,3-d]pyrimidin-7-amine***(4j).** Title compound **4j** was isolated as a yellow solid in 86.3% yield (2.05 g, 4.32 mmol), mp 171 – 173 °C. ^1^H NMR (600 MHz, CDCl_3_): *δ* 8.62 – 8.56 (m, 2H), 7.56 (d, *J* = 15.7 Hz, 1H), 7.39 – 7.33 (m, 2H), 7.09 (d, *J* = 15.7 Hz, 1H), 6.77 (s, 2H), 5.57 (t, *J* = 5.7 Hz, 1H), 4.98 (d, *J* = 5.6 Hz, 2H), 4.26 (s, 3H), 3.89 (s, 6H), 3.86 (s, 3H), 2.95 (t, *J* = 7.7 Hz, 2H), 1.88 – 1.84 (m, 2H), 1.02 (t, *J* = 7.4 Hz, 3H). ^13^C NMR (151 MHz, CDCl_3_): *δ* 157.7, 153.4, 150.2, 149.0, 148.1, 145.9, 143.5, 138.5, 135.7, 132.5, 128.2, 122.6, 121.0, 104.3, 61.1, 56.2, 43.9, 39.3, 27.8, 22.4,14.2. HR-MS (ESI): calcd for C_26_H_30_N_6_O_3_ [M + H]^+^, 475.2452; found 475.2452.

*(E)-N-(2-Methoxybenzyl)-1-methyl-3-propyl-5–(3,4,5-trimethoxystyryl)-1H-pyrazolo[4,3-d]pyrimidin-7-amine***(4k).** Title compound **4k** was isolated as a yellow solid in 79.8% yield (2.01 g, 3.99 mmol), mp 211 – 213 °C. ^1^H NMR (600 MHz, CDCl_3_): *δ* 7.83 (d, *J* = 15.7 Hz, 1H), 7.50 – 7.44 (m, 1H), 7.31 (td, *J* = 8.0, 1.5 Hz, 1H), 7.14 (d, *J* = 15.7 Hz, 1H), 6.97 (t, *J* = 7.9 Hz, 2H), 6.88 (s, 2H), 5.87 (t, *J* = 5.6 Hz, 1H), 4.96 (d, *J* = 5.6 Hz, 2H), 4.20 (s, 3H), 3.95 (s, 3H), 3.93 (s, 6H), 3.89 (s, 3H), 2.97 – 2.90 (m, 2H), 1.86 – 1.83 (m, 2H), 1.01 (t, *J* = 7.4 Hz, 3H). HR-MS (ESI): calcd for C_28_H_34_N_5_O_4_ [M + H]^+^, 504.2065; found 504.2067.

## 3. Biological evaluation

### 3.1. Cell culture

Mouse peritoneal RAW264.7 macrophages were obtained from BeNa Culture Collection Company. Cells were cultured in DMEM (Hyclone, USA) supplemented with 10% FBS (Biological Industries, Israel), 100 U/ml penicillin and 100 μg/ml streptomycin (Beyotime, China) at 37 °C in a humidified atmosphere containing 5% CO_2_.

### 3.2. Determination of NO, TNF-α and IL-6

RAW264.7 cells were seeded into 48-well plate with 6 × 10^4^ cells per well and incubated for 24 h. Cells were pretreated with the title compounds (10 μM) for 1 h, followed by exposure to LPS (0.5 μg/ml) for 24 h. The supernatants were collected and examined for NO production using Griess reagent (Beyotime, China). The levels of TNF-α and IL-6 in the supernatant were determined using the ELISA kit, according to the manufacturer's instructions (eBioScience, San Diego, CA).

### 3.3. Cell viability assay

RAW264.7 cells (6 × 10^3^ cells/per) were seeded into 96-well plate and incubated for 24 h. Cells were pretreated with the title compounds (20 μM) for 1 h and incubated with LPS (0.5 μg/ml) for 24 h. MTT solution (5 mg/ml in PBS) was added to each well and incubated for 4 h  at 37 °C. The MTT containing media was removed and then 150 μL of DMSO was added. The absorbance was detected at 490 nm by a microplate reader (MQX200, Bio-Tek, USA).

### 3.4. Western blotting

RAW264.7 cells were seeded into a 6-well plate at a density of 3 × 10^5^ cells per well and then cultured for 24 h. Cells were pretreated with compound **4e** (2, 1, 0.5 μM) for 1 h, followed by exposure to LPS (0.5 μg/ml) for 0.5 h.

Cells were lysed with RIPA lysis buffer (Beyotime, China) containing PMSF and phosphatase inhibitors, and then incubated on ice for 30 min. The protein lysates were separated by 12% SDS-PAGE and subsequently transferred onto PVDF membranes (GE Healthcare, UK). The blocked membranes were incubated with the indicated primary antibodies at 4 °C overnight (All of the antibodies were purchased from Cell Signaling Technology, USA). After washing three times with TBST (Beyotime, China), the membranes were incubated with HRP-conjugated secondary antibody (Beyotime, China) for 1 h at room temperature.

### 3.5. *In vivo* experiment

Male C57BL/6 mice weighing 18 – 22 g were purchased from Animal Department of Anhui Medical University. Mice were randomly divided into five groups (*n* = 8): physiological saline as negative control group, LPS (20 mg/kg) stimulated group, compound **4e** high dose (20 mg/kg) group, compound **4e** low dose (10 mg/kg) group and celecoxib (15 mg/kg) as positive control group. Compound **4e** or celecoxib was administrated intraperitoneally (i.p.) 0.5 h before LPS injection via tail vein. Mice were anesthetized and sacrificed 48 h after LPS injection. Lung tissues were collected and fixed in 4% paraformaldehyde, followed by embedded in paraffin. After dehydration, sections were stained with Hematoxylin and Eosin (H&E) staining.

## 4. Results and discussion

### 4.1. Chemistry

4-Amino-1-methyl-3-propyl-4,5-dihydro-1H-pyrazole-5-carboxamide (**SM**) was prepared according to previous protocol^21^. **SM** was treated with substituted carboxylic acid in the presence of EDCI and HOBt to yield **A1**–**A4**. Compounds **B1**–**B4** were synthesized from compounds **A1**–**A4***via* cyclization in the presence of sodium ethoxide. Key intermediates **C1**–**C4** were carried out under N_2_ atmosphere with POCl_3_ at 95 °C for 8 h (Scheme 1). (General procedures see Supporting Information).

### 4.2. Inhibitory activity against LPS-induced NO release

**Figure 3.**
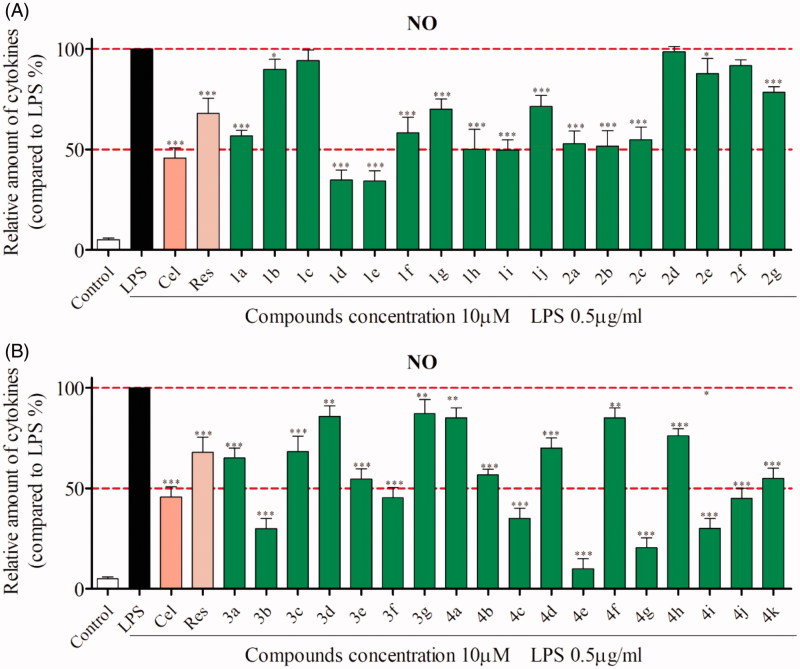
Inhibition of LPS-induced NO releasing by compounds **1a∼4k** in RAW264.7 cells^a^. ^a^RAW264.7 cells were pretreated with compounds (10 μM) for 1 h, and incubated with LPS (0.5 μg/ml) for 24 h. The levels of NO releasing were measured using Griess Reagent assay. (**A**) Effect of compounds **1a∼2g** on NO secretion. (**B**) Effect of compounds **3a∼4k** on NO secretion. Celecoxib (Cel) and resveratrol (Res) were chosen as positive controls. ***p < .001, **p < .01, *p < .05 versus LPS group.

Nitric oxide (NO) is an important pro-inflammatory mediator, relating to several inflammatory diseases, such as rheumatoid arthritis, chronic hepatitis and ALI^22^^,^^23^. Therefore, inhibition of its overproduction may provide a useful therapy for inflammatory diseases^24^. Treatment of RAW 264.7 cells with LPS stimulated NF-κB signaling pathway, resulting in the production of cytokines including NO, IL-6 and TNF-α. Briefly, RAW 264.7 cells were pre-incubated with tested compounds, followed by incubated with LPS. The supernatants were collected and then nitrite levels were determined. The results indicated that almost all tested compounds were able to inhibit NO production at 10 µM (Figure 3). Being the most potent, compound **4e** reduced NO release more intensely than both celecoxib and resveratrol. Accordingly, the introduction of 3-morpholinopropan-1-amine group at C-7 of pyrazolo[4,3-*d*]pyrimidine scaffold can improve the inhibitory activity.

### 4.3. Assessment of toxicity

**Figure 4.**
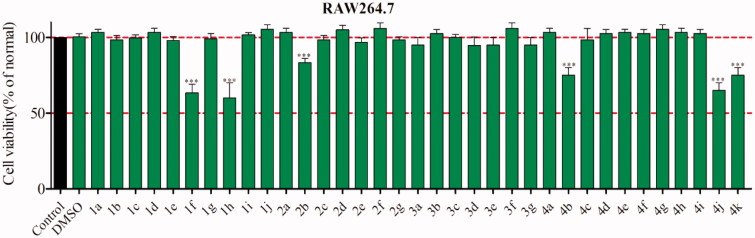
The cytotoxicity of compounds **1a∼4k** in RAW264.7 cells^a^. ^a^The cell viability was evaluated by the MTT assay. ***p < .001 compare with control group.

To discard that the suppressive effects on NO release was related to cell viability, MTT assay was adopted. As observed in Figure 4, cell viability was not affected by most of compounds at 20 µM, excepting of compounds **1f**, **1h, 2b, 4b**, **4j** and **4k** with weak cytotoxicity. The results indicated that their anti-inflammatory activity is not mediated by cytotoxic effect. Therefore, these compounds were worth of further evaluation.

### 4.4. Inhibition of LPS-induced release of cytokines

**Figure 5.**
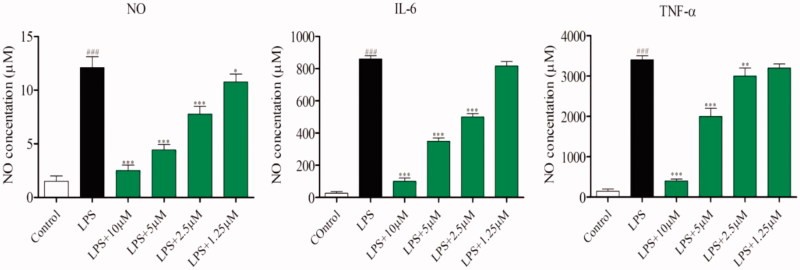
Inhibition of the cytokines production^a^. ^a^RAW264.7 cells were pretreated with compound **4e** in a series of concentrations (10, 5, 2.5, 1.25 μM) for 1 h, incubated with LPS (0.5 μg/ml) for 24 h. NO releasing was measured using Griess Reagent assay. The levels of TNF-α and IL-6 in the culture medium were measured by ELISA. *** *p*< .001, **p < .01, *p < .05 versus LPS group.

Increasing evidences have showed that the two important cytokines IL-6 and TNF-α play important roles in the pathogenesis of ALI through a series of cytokine signaling pathways^25^. Therefore, the most potent compound **4e** was chosen to further evaluate for inhibition of LPS-induced NO, TNF-α and IL-6 releasing. As shown in Figure 5, compound **4e** significantly decreased NO, IL-6 and TNF-α secretion in a concentration-dependent manner, with IC_50_ values of 2.64, 4.38 and 5.63 μM, respectively. On the basis of above findings, the anti-inflammatory mechanisms of compound **4e** were further explored.

### 4.5. Inhibition of LPS-induced TLR4 expression

**Figure 6.**
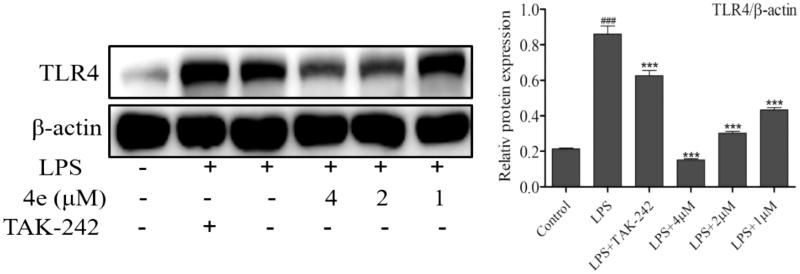
Inhibit expression of LPS-induced TLR4^a^. ^a^RAW264.7 cells were pretreated with compound **4e** (1 – 4 µM) for 1 h, then stimulated with LPS (0.5 μg/ml) for 24 h. The expression of TLR4 was analyzed by Western blot. The results were showed as means ± SD (*n* = 3) of at least three independent experiments. TAK-242 was the TLR4 inhibitor. *^###^p* < .001 compared with LPS un-stimulated cells; *p < .05, **p < .01, ***p < .001 compare with LPS-stimulated cells.

As a key factor in LPS-induced inflammation, TLR4 activates series of cellular signaling pathways, including NF-ĸB and mitogen-activated protein kinases (MAPKs), leading to the secretion of pro-inflammatory cytokines, such as NO, IL-6, TNF-α and IL-1β^26–28^. Therefore, we investigated whether the expression of TLR4 was down-regulated by compound **4e**. As shown in Figure 6, LPS-induced TLR4 overexpression was attenuated by pretreatment of compound **4e** in a concentration-dependent manner.

### 4.6. Inhibition of LPS-induced p38 signaling pathway

**Figure 7.**
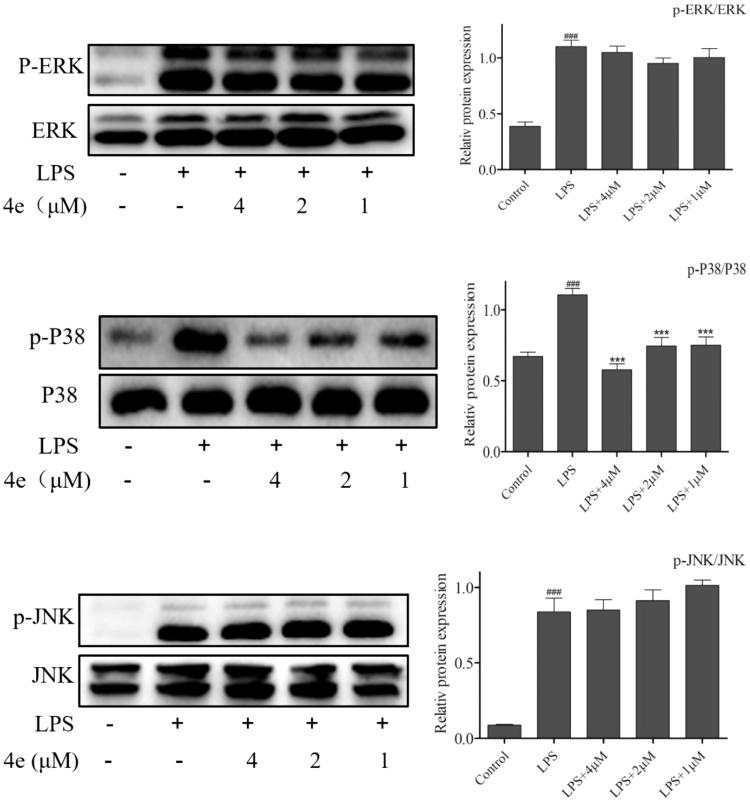
Suppressed LPS-induced p38 activation^a^. ^a^RAW264.7 cells were pretreated with compound **4e** (1–4 µM) for 1 h, then stimulated with LPS (0.5 μg/ml) for 30min. The phosphorylation and total expression of ERK, JNK and p38 were analyzed by Western blot. The results were showed as means ± SD (*n* = 3) of at least three independent experiments. *^###^p* < .001 compared with LPS un-stimulated cells; *p < .05, **p < .01, ***p < .001 compare with LPS-stimulated cells.

MAPKs, including ERK, p38 and JNK, were quite significant in the regulation of inflammation^29^. Therefore, we detected the effects of compound **4e** on LPS-mediated MAPKs signaling activation. As shown in Figure 7, the phosphorylation of p38 but not JNK or ERK was blocked by compound **4e** treatment in a concentration-dependent manner. And the total levels of ERK, JNK and p38 were not affected.

### 4.7. *In vivo* experiment

**Figure 8.**
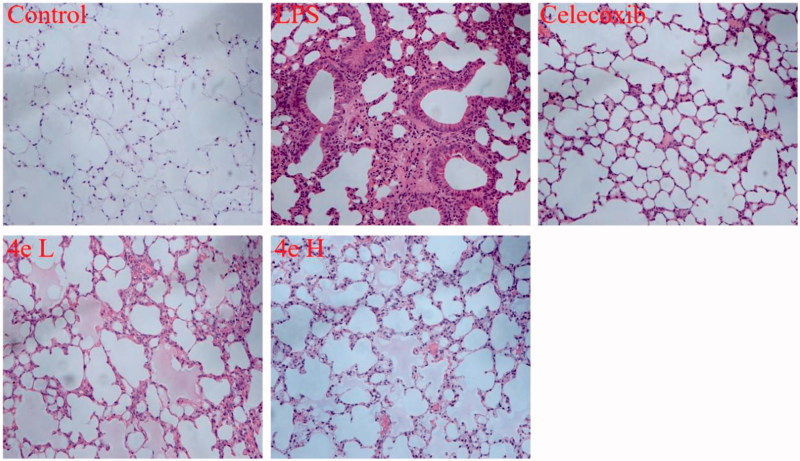
Compound **4e** protected LPS-induced acute lung injury^a^. ^a^C57/BL6 mice were treated with compound **4e** (10 mg/kg, 20 mg/kg), and after 30 min, were challenged with 20 mg/kg LPS by tail vein injection. After Hematoxylin and Eosin staining, histological examination was performed by light microscopy (magnification ×200). Celecoxib (15 mg/kg) was a positive drug. *^###^p* < .001 compared with control group; *p < .05, **p < .01, ***p < .001 compare with LPS group.

Next, compound **4e** was evaluated in LPS-induced ALI mice model. Mice were pretreated with compound **4e** by intraperitoneal injection 0.5 h before LPS challenge. LPS stimulation leads to significant pro-inflammatory alterations, including lung edema, inflammatory cell infiltration and destruction of alveolar structure. Pretreatment of mice with compound **4e** effectively reduced airspace inflammation and amended the tissue structure of pulmonary lobules (Figure 8). These results indicated the protective effects of compound **4e** against LPS-induced ALI in mice model.

## 5. Conclusions

In the present studies, 35 novel pyrazolo[4,3-*d*]pyrimidine derivatives were designed, synthesized and evaluated for their anti-inflammatory activities in RAW264.7 cells. The preliminary SAR studies show that the introduction of 3-morpholinopropan-1-amine group into pyrazolo[4,3-*d*]pyrimidine could increase anti-inflammatory activity. Specifically, the most potent compound **4e** was selected to further study the mechanism. The results showed that compound **4e** concentration-dependently inhibited LPS-induced NO, IL-6 and TNF-α secretion through suppressing TLR4/p38 signaling pathway. *In vivo* studies in LPS-challenged mice showed that compound **4e** effectively normalized pulmonary histopathological changes. Taken together, compound **4e** could be potential therapeutics for ALI.

## Supplementary Material

Supplemental Material
