# Supplementary material for: Design and synthesis of novel pyrazolo[4,3-d]pyrimidines as potential therapeutic agents for acute lung injury
Source: J Enzyme Inhib Med Chem. 2019 May 23;34(1):1121–30. doi: 10.1080/14756366.2019.1618291 (PMC6534230; doi:10.1080/14756366.2019.1618291)

Supporting Information

Design and Synthesis of Novel Pyrazolo[4,3-*d*]pyrimidines as Potential Therapeutic Agents for Acute Lung Injury

Table of Contents

PART 1. General procedure for the synthesis of intermediates **C1~C4**……….......S1

PART 2. ^{1}H NMR and ^{13}C NMR data of **1a~1j, 2a~2g, 3a~3g and 4a~4k**..……...S2

PART 3. HR-MS data of **1a~1j, 2a~2g, 3a~3g and 4a~4k**.………….……….……..S3

PART 1. General procedure for the synthesis of intermediates C1~C4

3,4,5-trimethoxybenzoic acid (1.06 g, 5 mmol), EDCI (1.438 g, 7.5 mmol), HOBt (1.013 g, 7.5 mmol), 4-amino-1-methyl-3-propyl-5-pyrazolecarboxamide (**SM**) (0.922 g, 5 mmol) and TEA (0.94 mL, 0.75 mmol) were dissolved in dry DCM (20 mL), and the mixture was stirred at room temperature for 18~20 h. DCM was removed under reduced pressure after the reaction was completed. Residue and sufficient water are mixed at room temperature，the intermediate **A1** was obtained as white solid by suction filtration. And intermediates **A2~A4** were obtained using the same procedure as intermediate **A1**.

To a solution of the sodium ethoxide (0.340 g, 5 mmol) was dissolved in ethanol (20 mL) and added intermediate **A1** (1.88 g, 5 mmol), and the reaction mixture were stirred at refluxed for 8~12 h. The resulting solution was concentrated under reduced pressure and the residue was acidified with hydrochloric acid (15%, v/v) to pH 7. The intermediate **B1** was obtained as white solid by suction filtration. And intermediate **B2~B4** were obtained using the same procedure as intermediate **B1**.

To a solution of intermediate **B1** (1.79 g, 5 mmol) were dissolved in phosphorus oxychloride ($POCl_3$, 15 mL), and the reaction mixture were stirred at 95 ^oC, for 10~12 h under N_2 atmosphere. The excess POCl3 was removed under reduced pressure after the reaction was completed. Slowly pour the oily residue into ice water and mix quickly and extracted with DCM (50 mL×3). The organic layer was washed with saturated $NaHCO_3$ solution and saturated brine solution, dried over Na_2SO_4, and concentrated under reduced pressure. The intermediate **C1** was obtained as yellow solid. And intermediates **C2~C4** were obtained using the same procedure as intermediate **C1**.

PART 2. ^{1}H NMR and ^{13}C NMR data of 1a~1j, 2a~2g, 3a~3g and 4a~4k.

^{1}H NMR and ^{13}C NMR for 1a

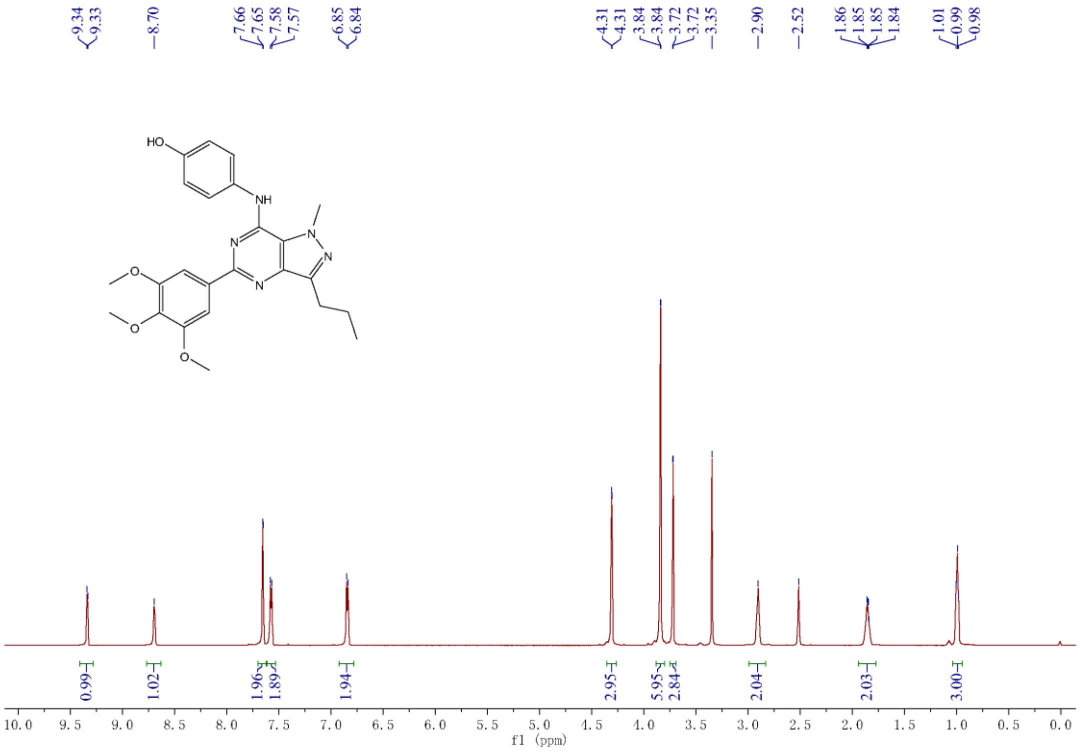

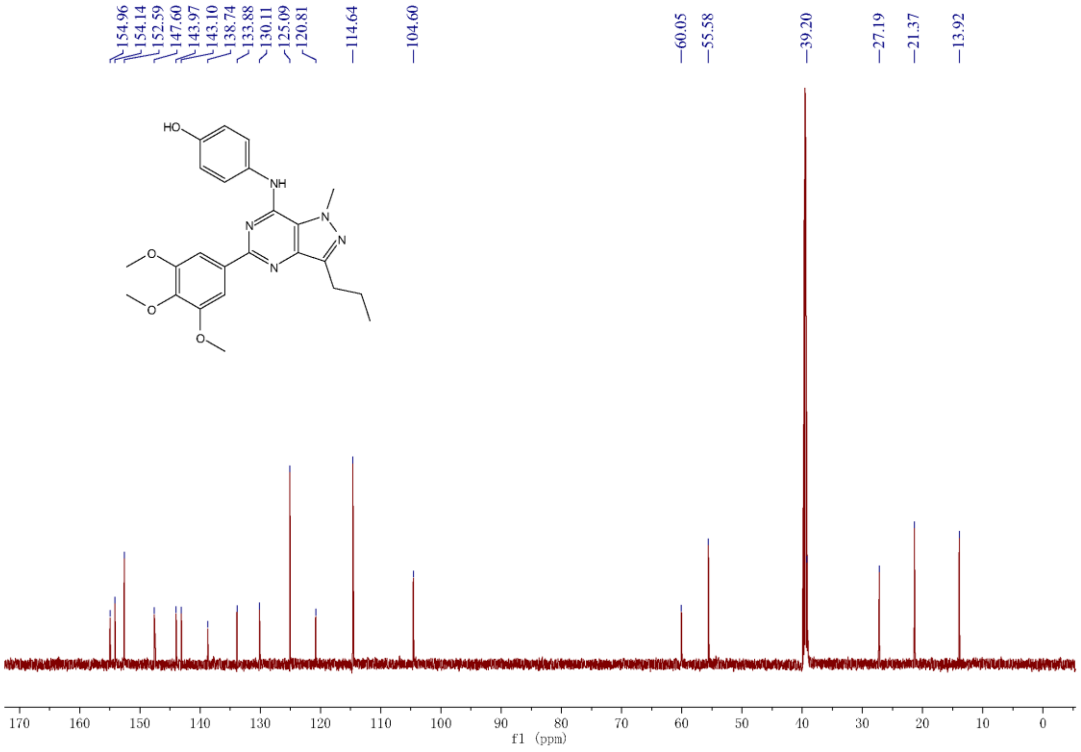

^{1}H NMR for *1b*

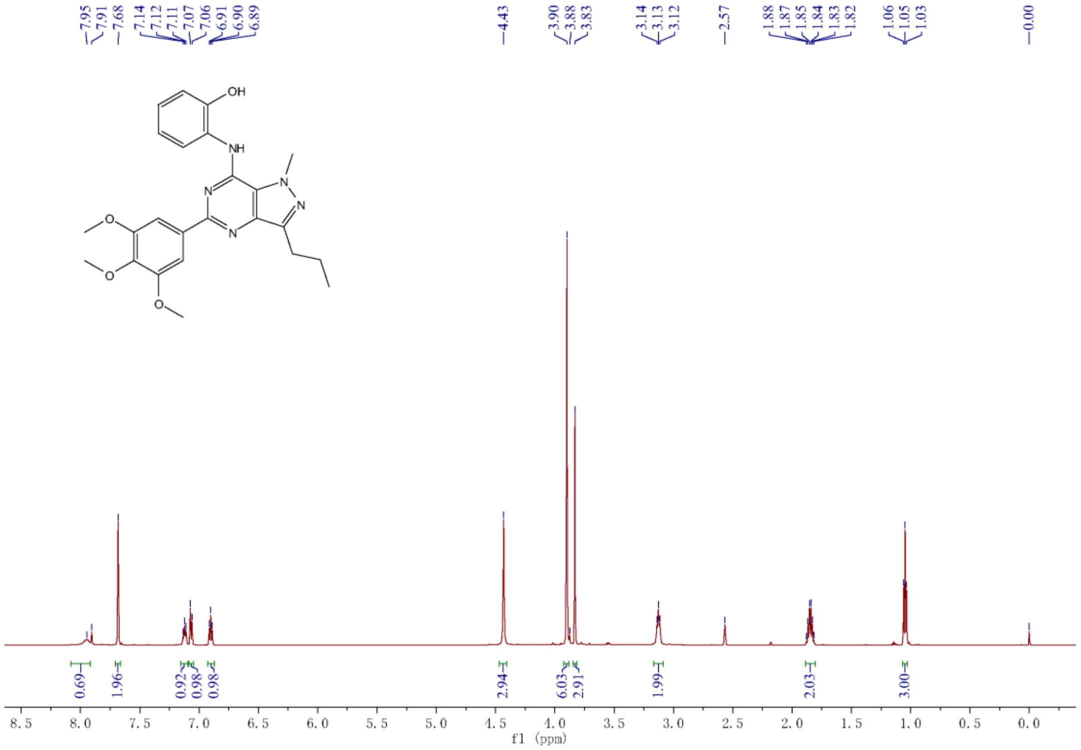

^{1}H NMR and ^{13}C NMR for *1c*

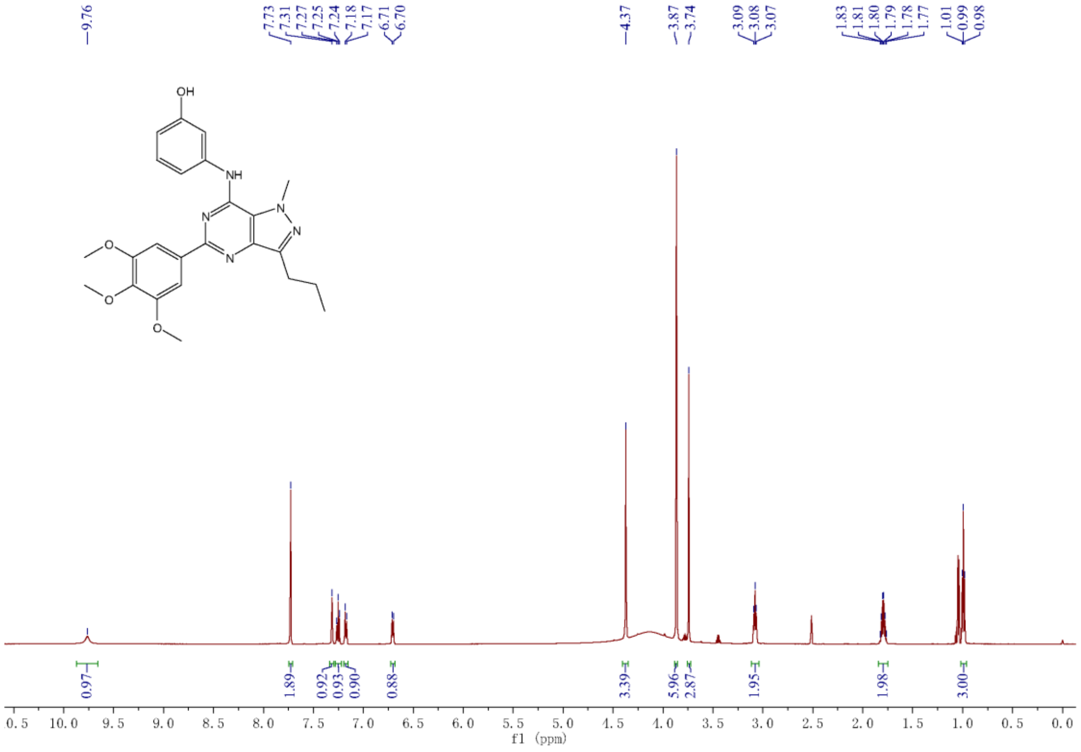

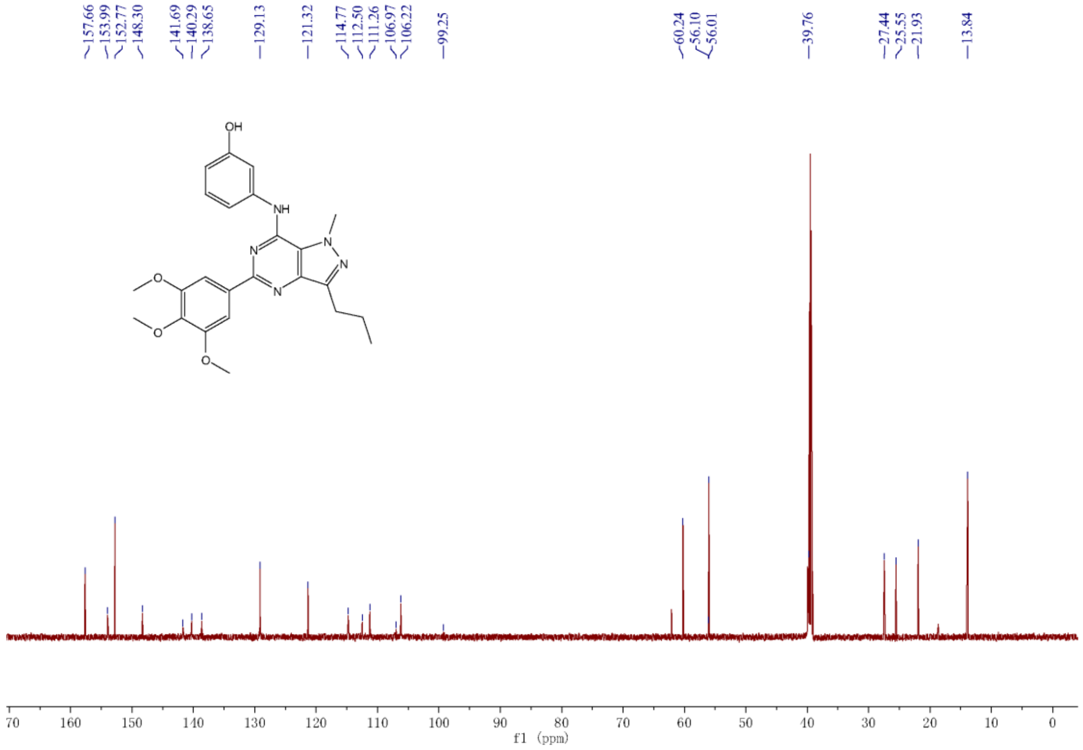

^{1}H NMR and ^{13}C NMR for *1d*

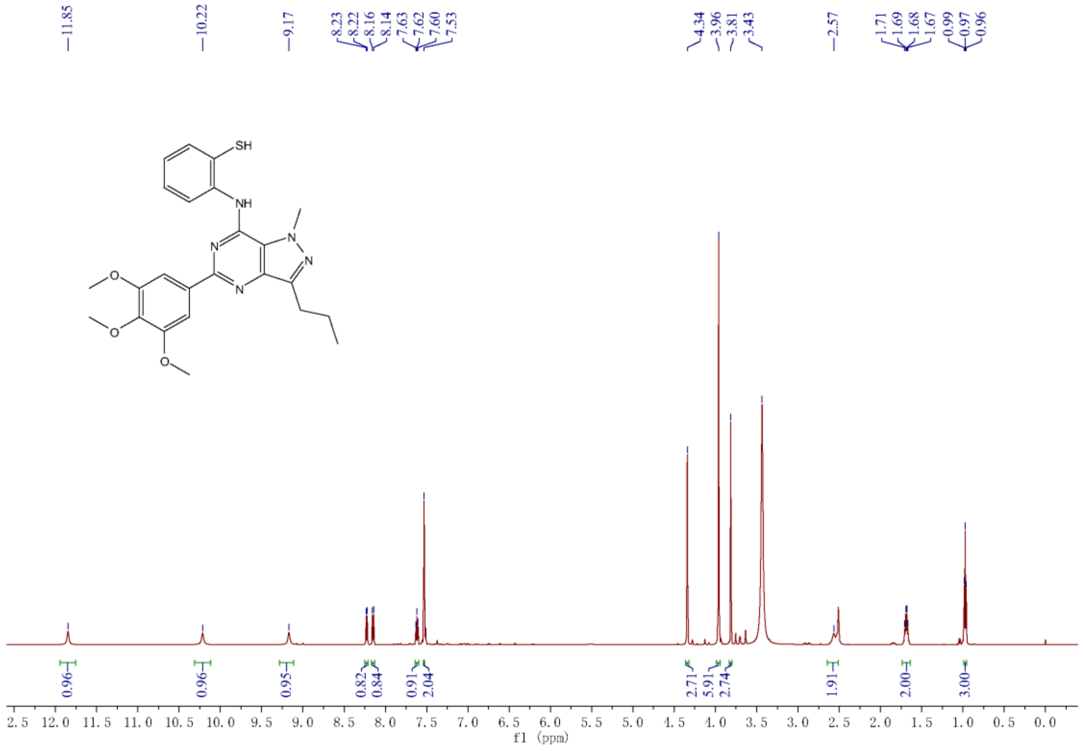

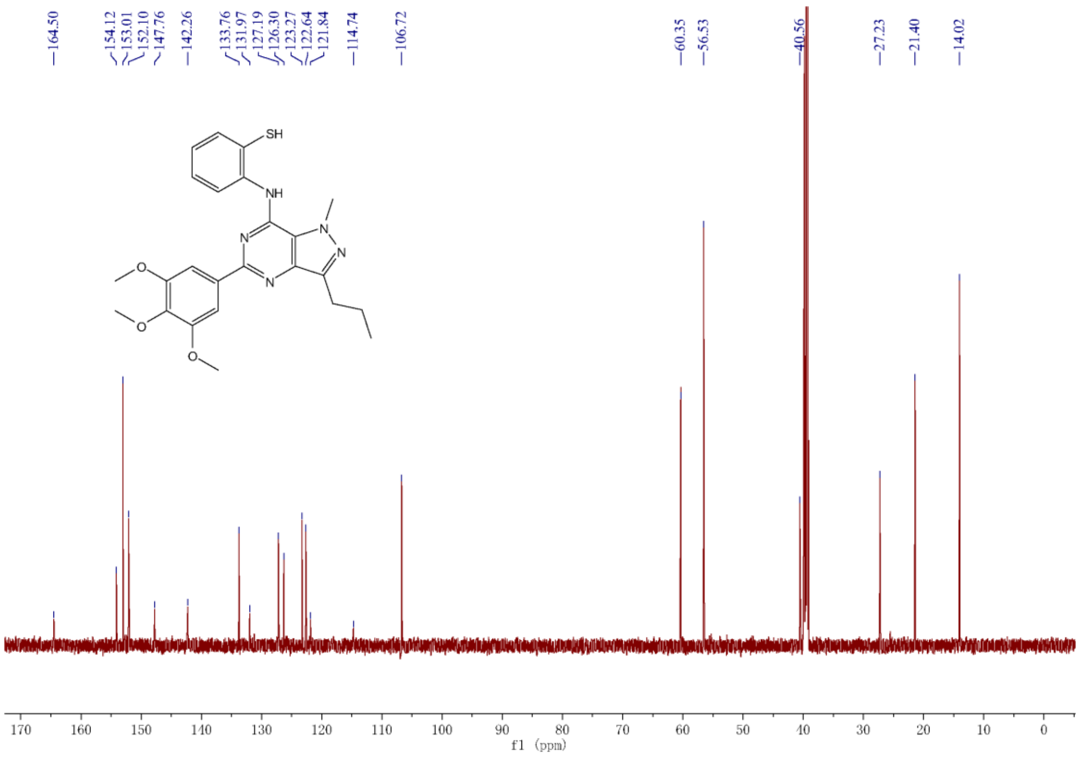
164.50
154.12
153.01
152.10
147.76
142.26
133.76
131.97
127.19
126.30
123.27
122.64
121.84
114.74
106.72
60.35
56.53
40.56
27.23
21.40
14.02
SH
NH
N
N
N
N
O
O
O
170 160 150 140 130 120 110 100 90 80 70 60 50 40 30 20 10 0
f1 (ppm)

^{1}H NMR for *1e*

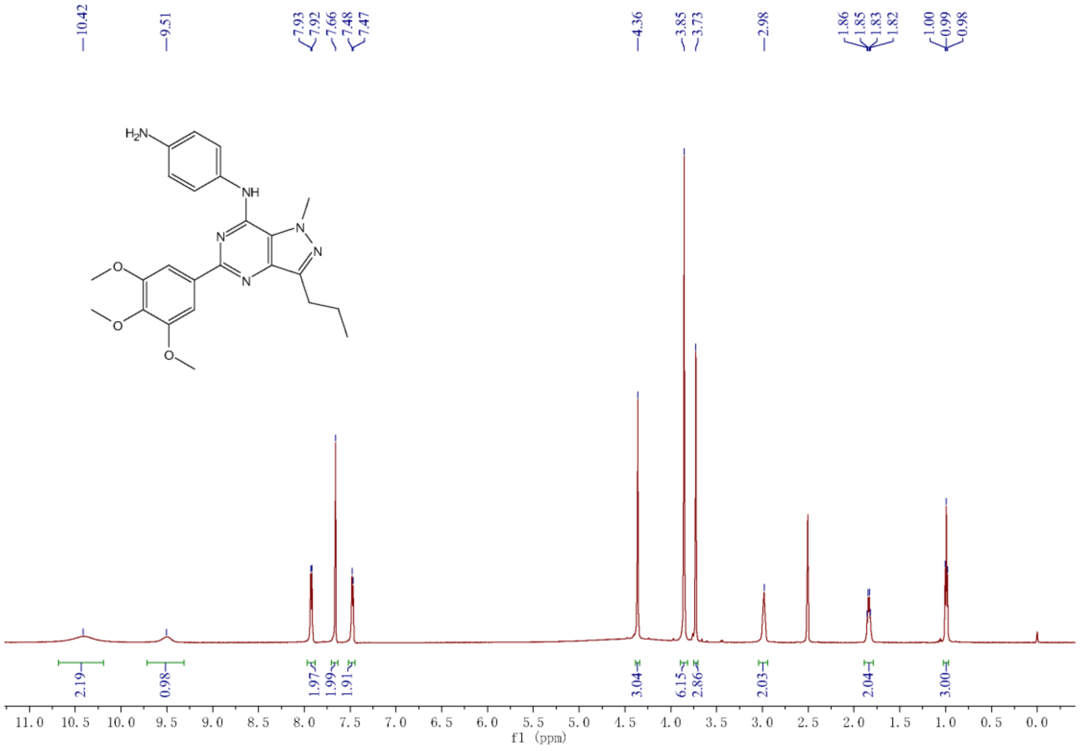
10.42
9.51
7.93
7.92
7.66
7.48
7.47
4.36
3.85
3.73
2.98
1.86
1.85
1.83
1.82
1.00
0.99
0.98
H2N
NH
N
N
N
N
O
O
O
2.19
0.98
1.97
1.99
1.91
3.04
6.15
2.86
2.03
2.04
3.00
11.0 10.5 10.0 9.5 9.0 8.5 8.0 7.5 7.0 6.5 6.0 5.5 5.0 4.5 4.0 3.5 3.0 2.5 2.0 1.5 1.0 0.5 0.0
f1 (ppm)

^{1}H NMR and ^{13}C NMR for *1f*

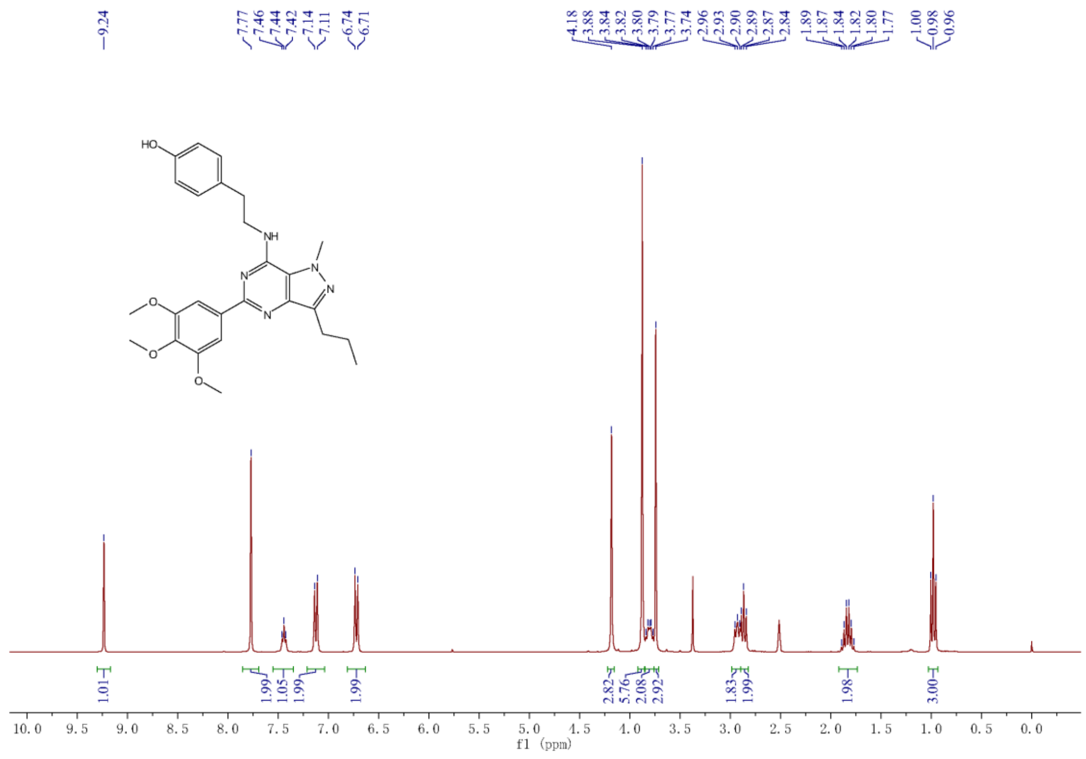

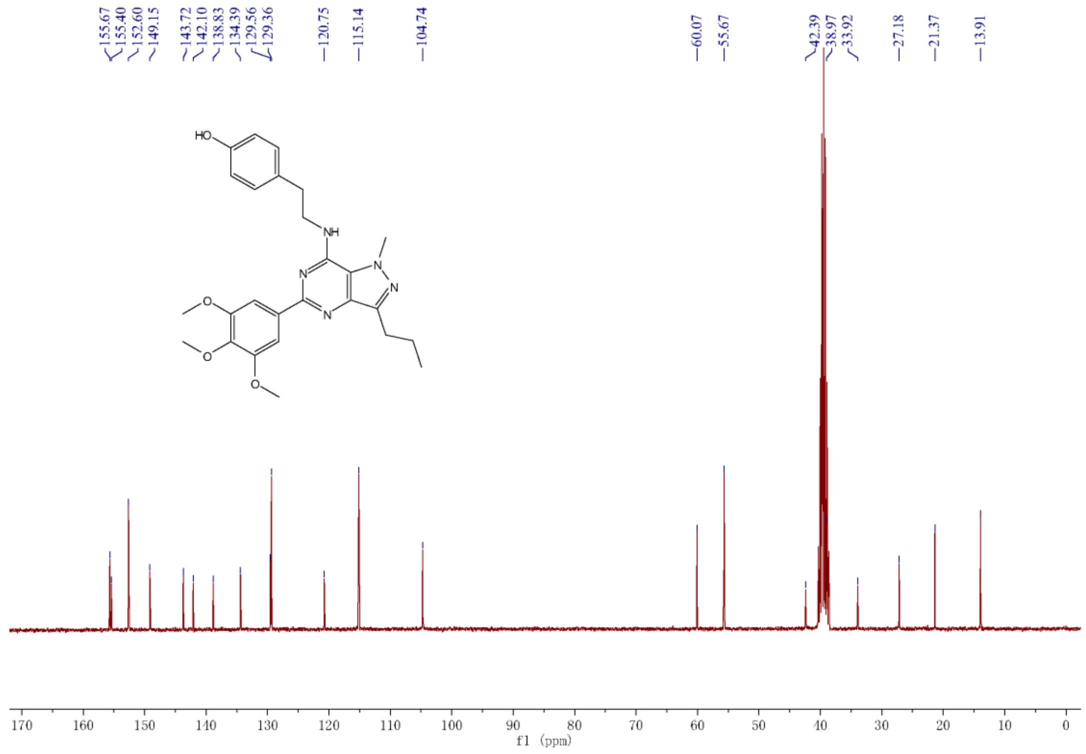

^{1}H NMR for *1g*

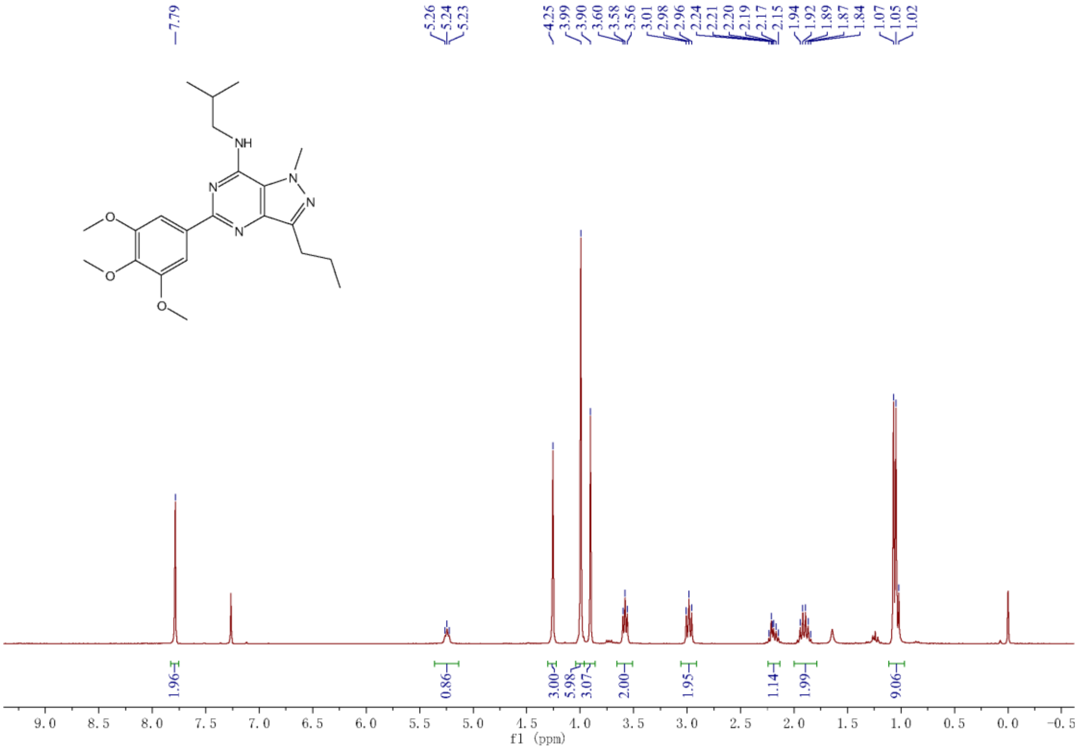

^{1}H NMR and ^{13}C NMR for *1h*

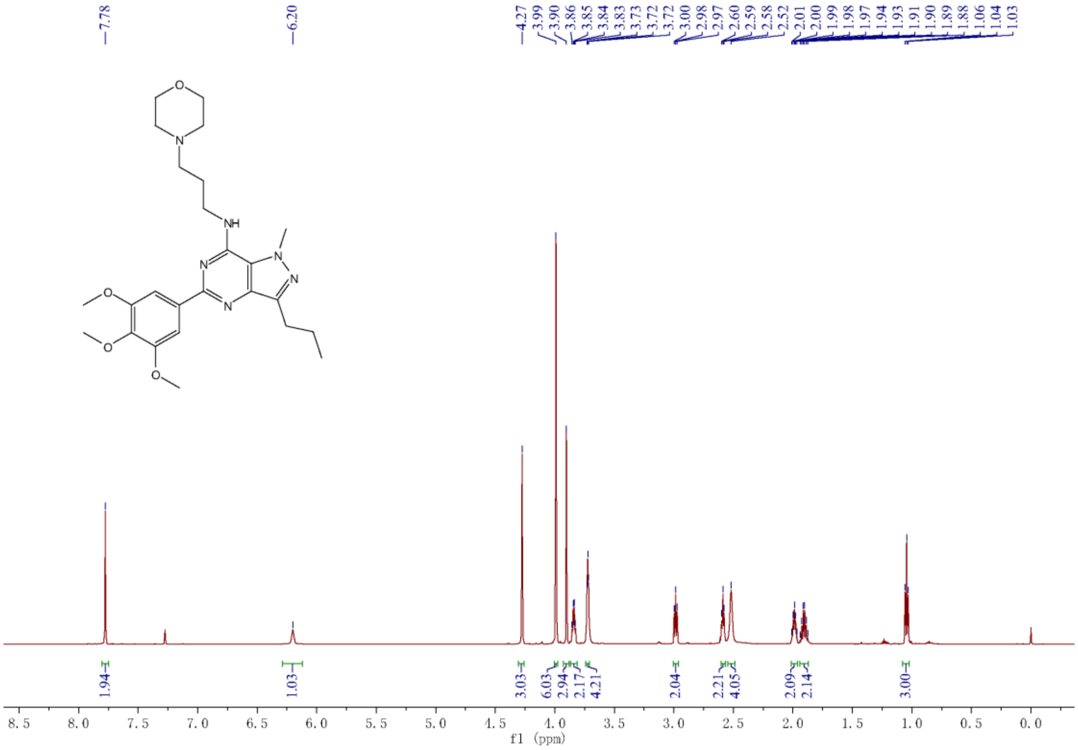

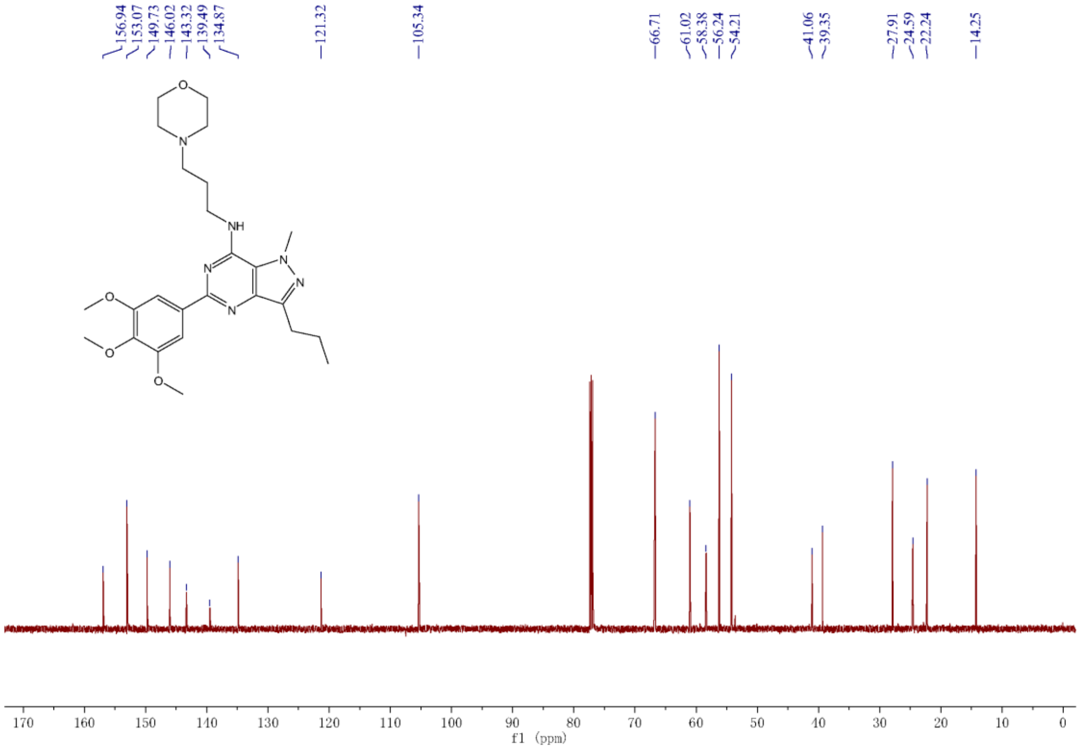

^{1}H NMR and ^{13}C NMR for *1i*

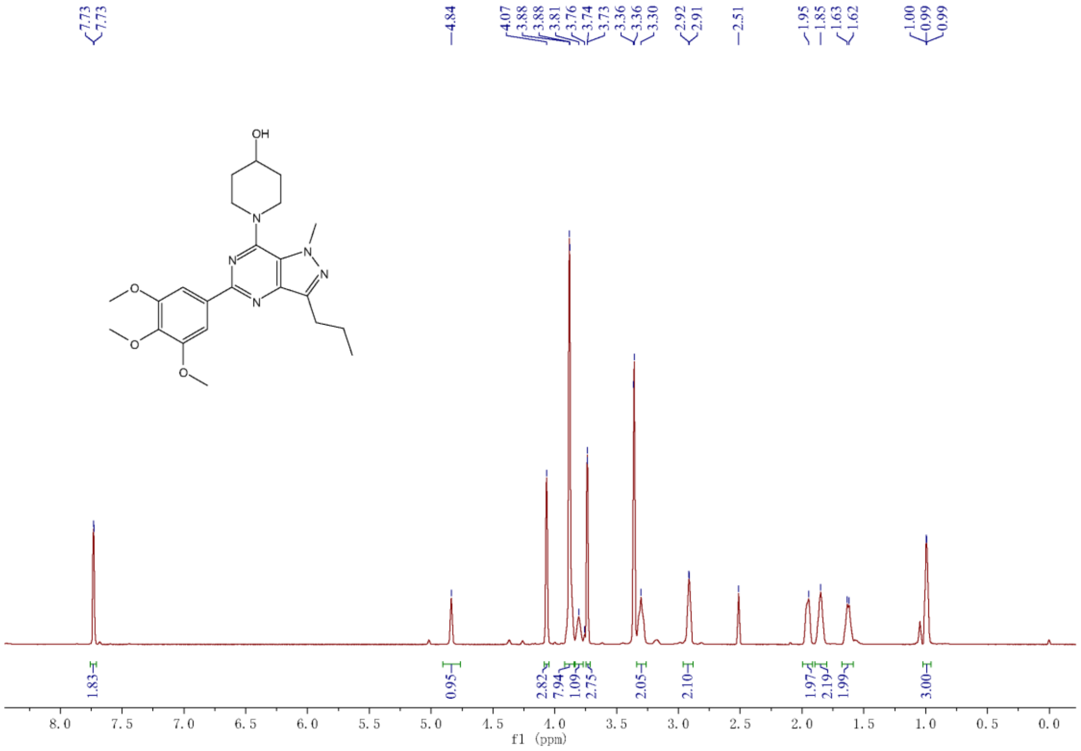

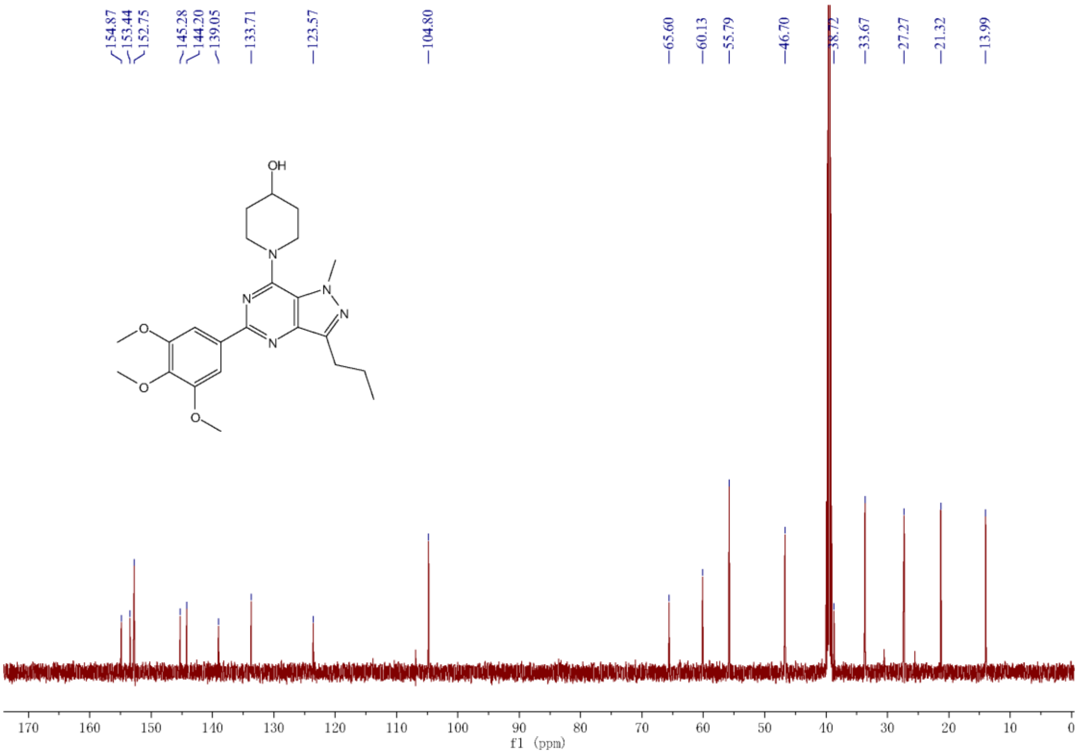

^{1}H NMR and ^{13}C NMR for *1j*

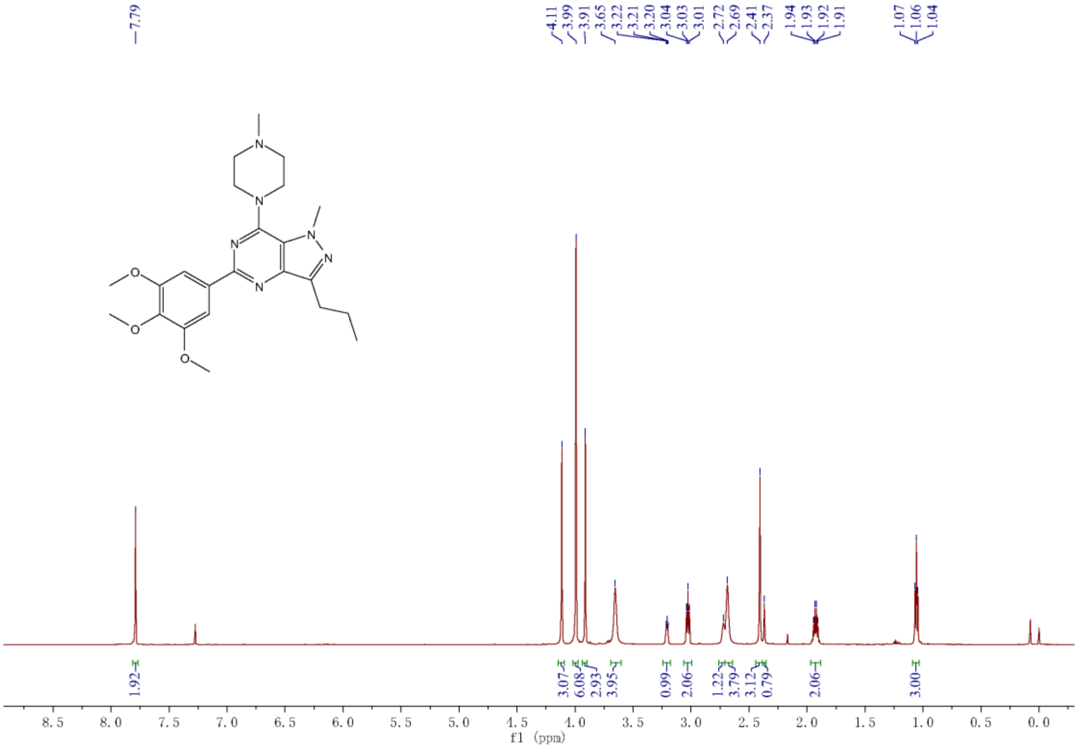

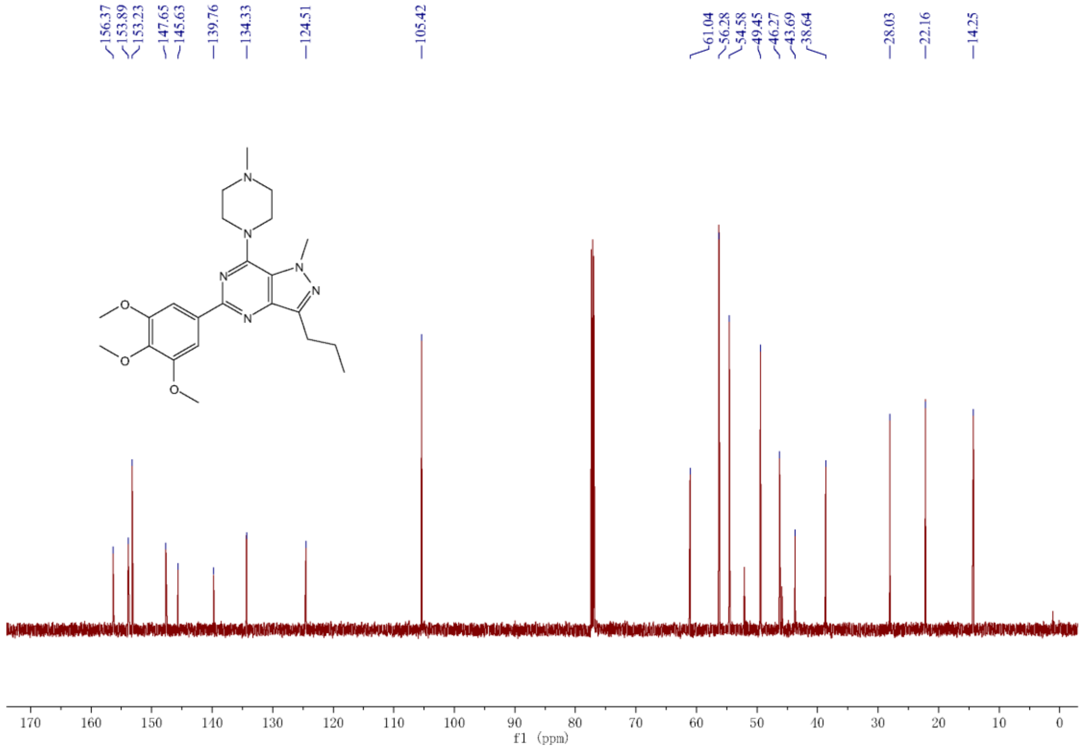

^{1}H NMR and ^{13}C NMR for *2a*

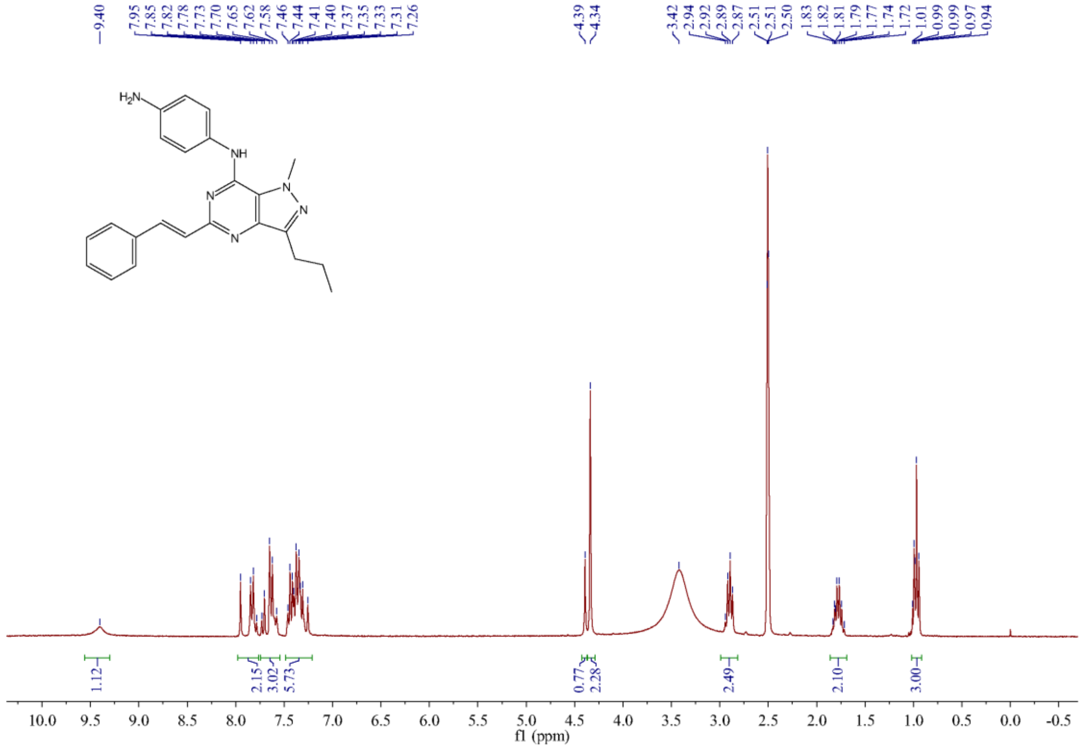

^{1}H NMR and ^{13}C NMR for *2b*

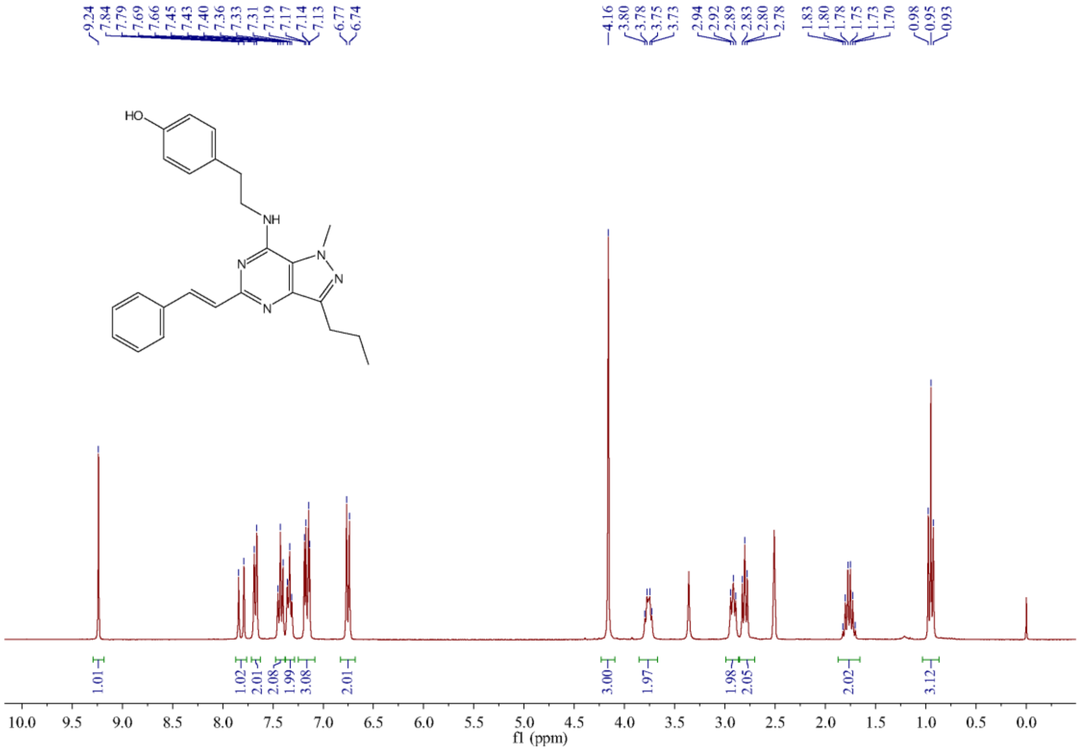

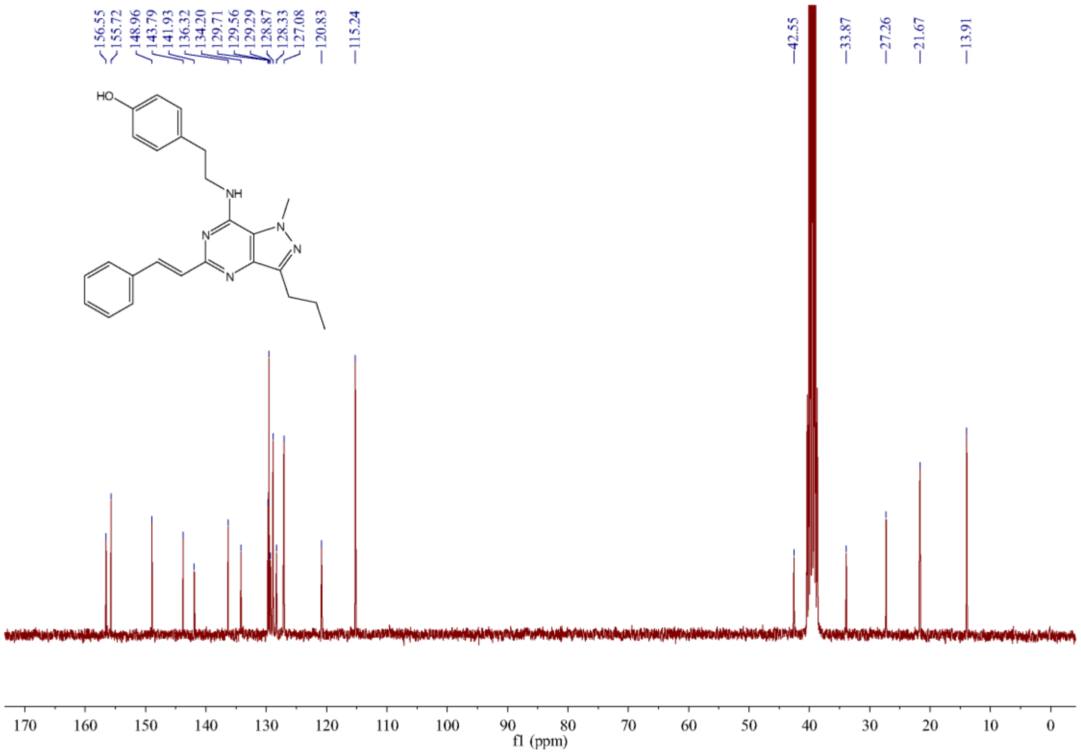

^{1}H NMR and ^{13}C NMR for *2c*

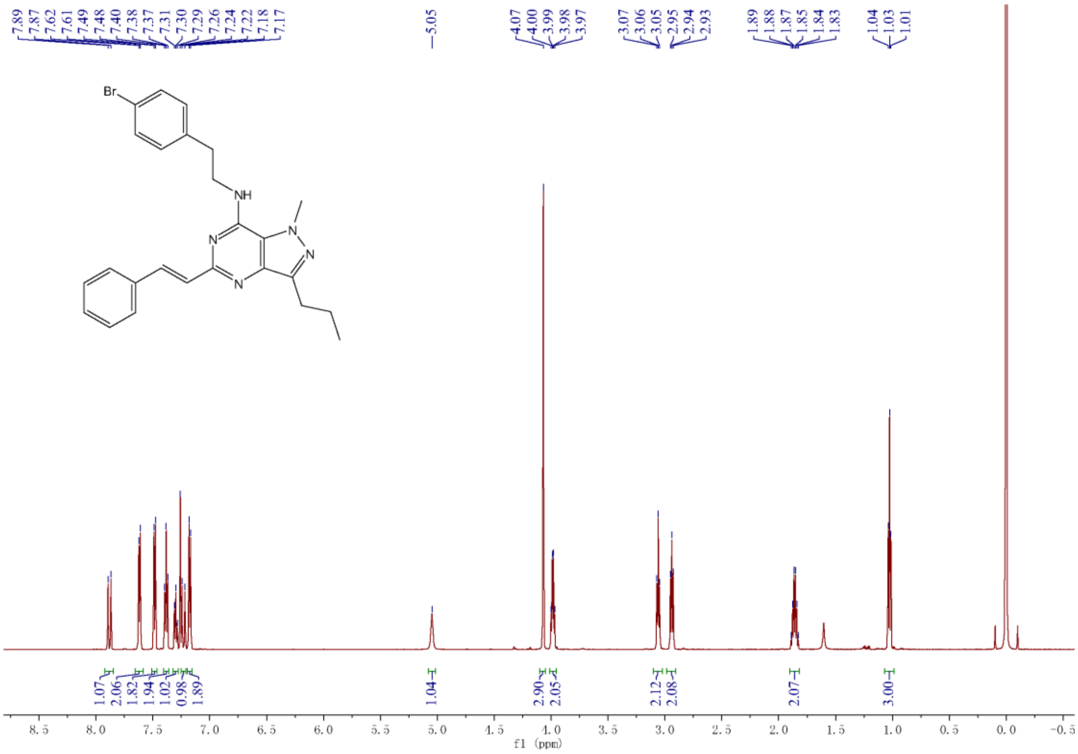

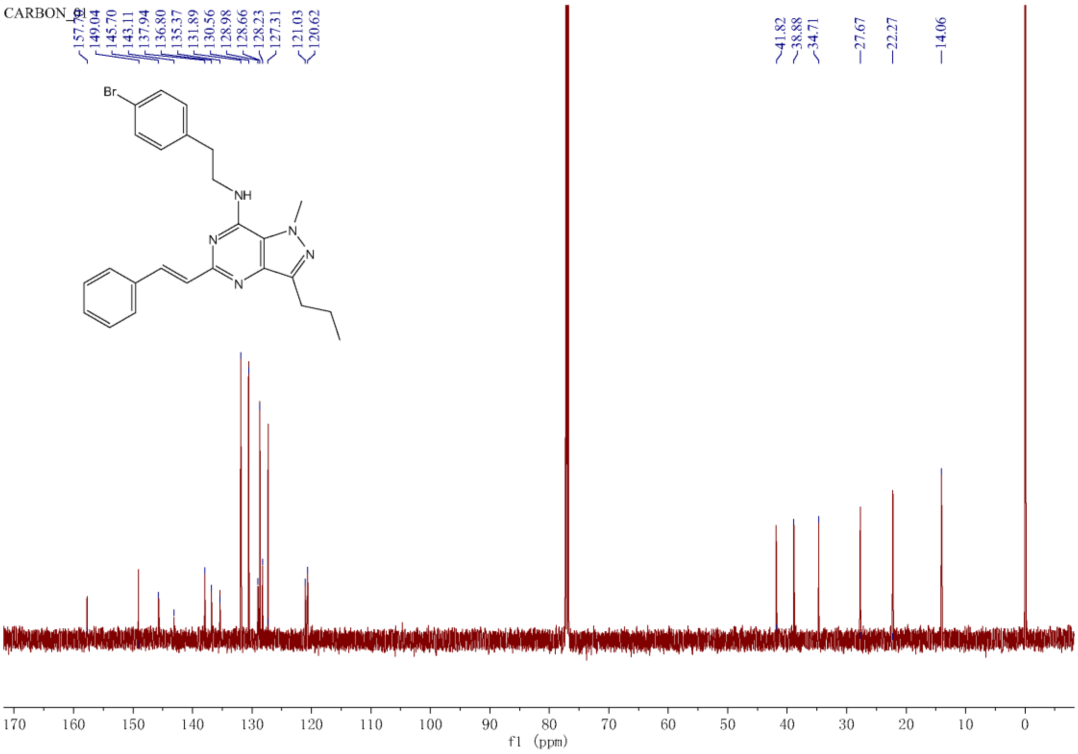

^{1}H NMR and ^{13}C NMR for *2d*

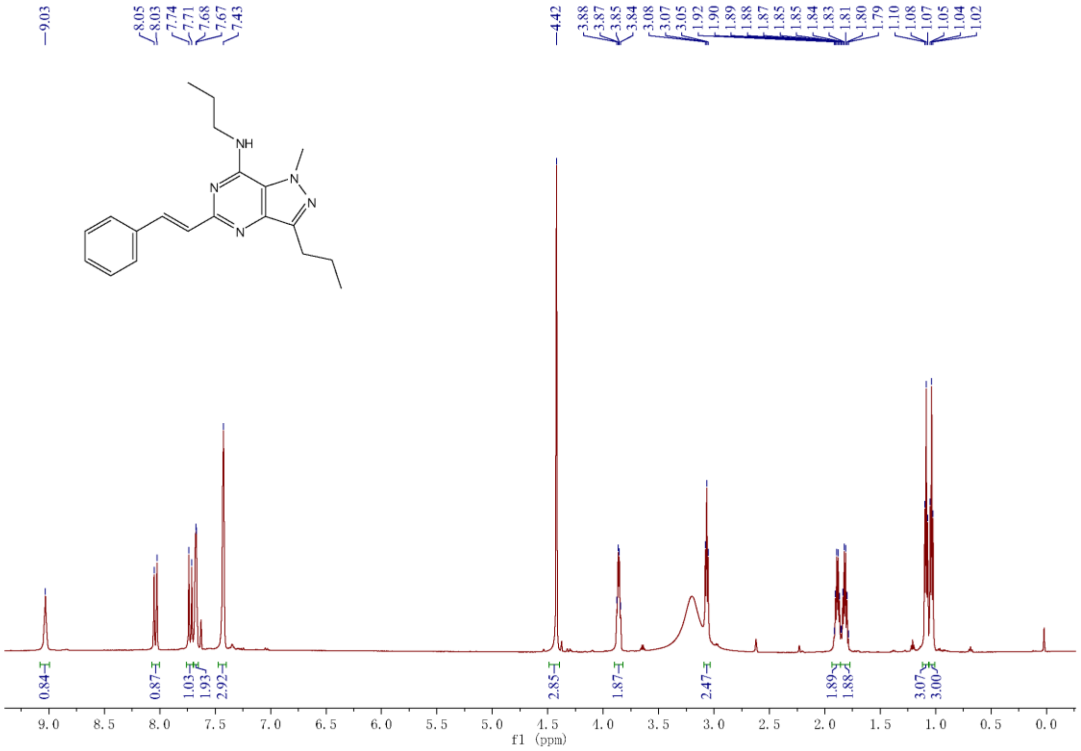

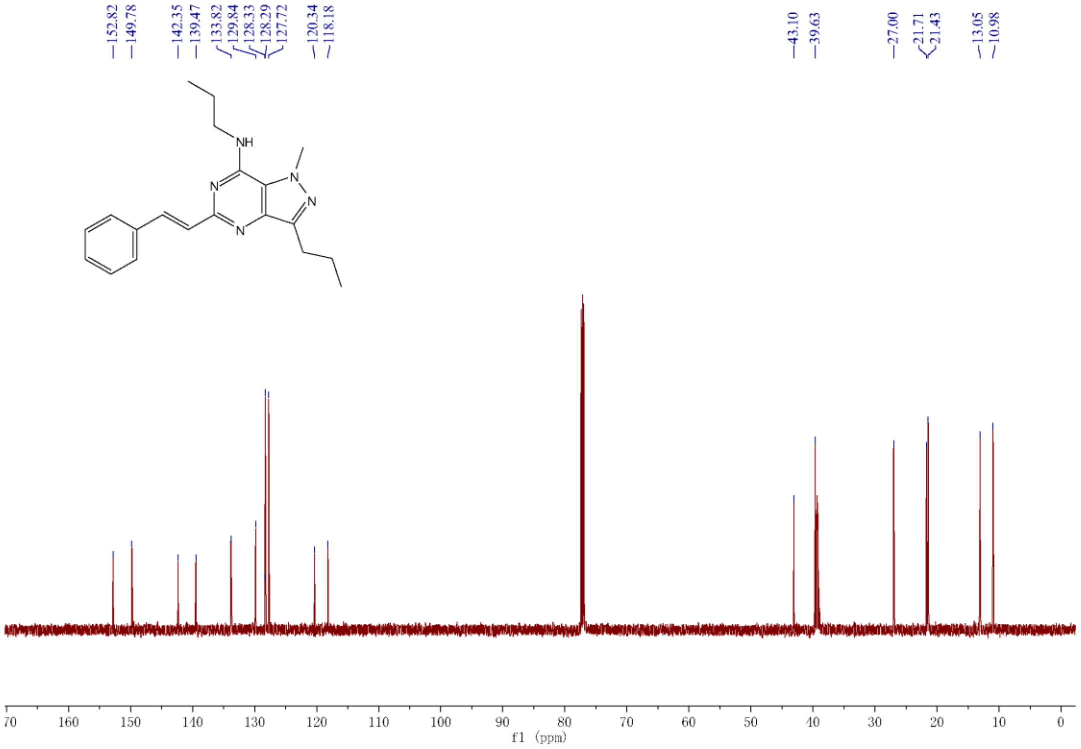

^{1}H NMR for *2e*

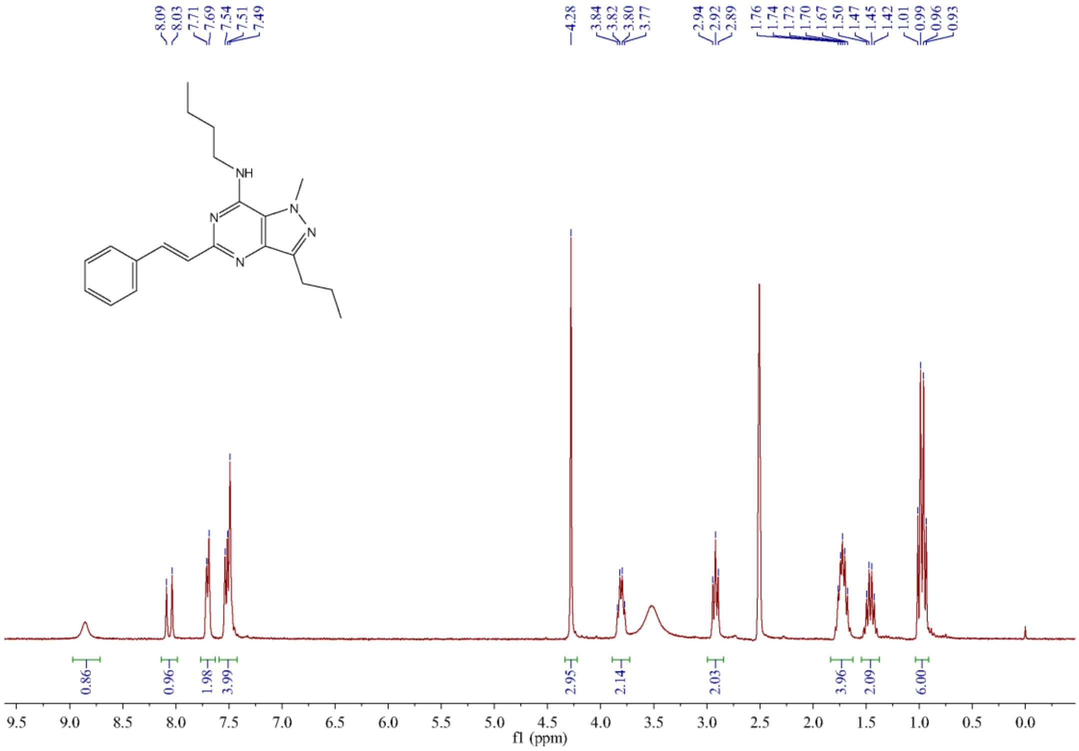

^{1}H NMR for *2f*

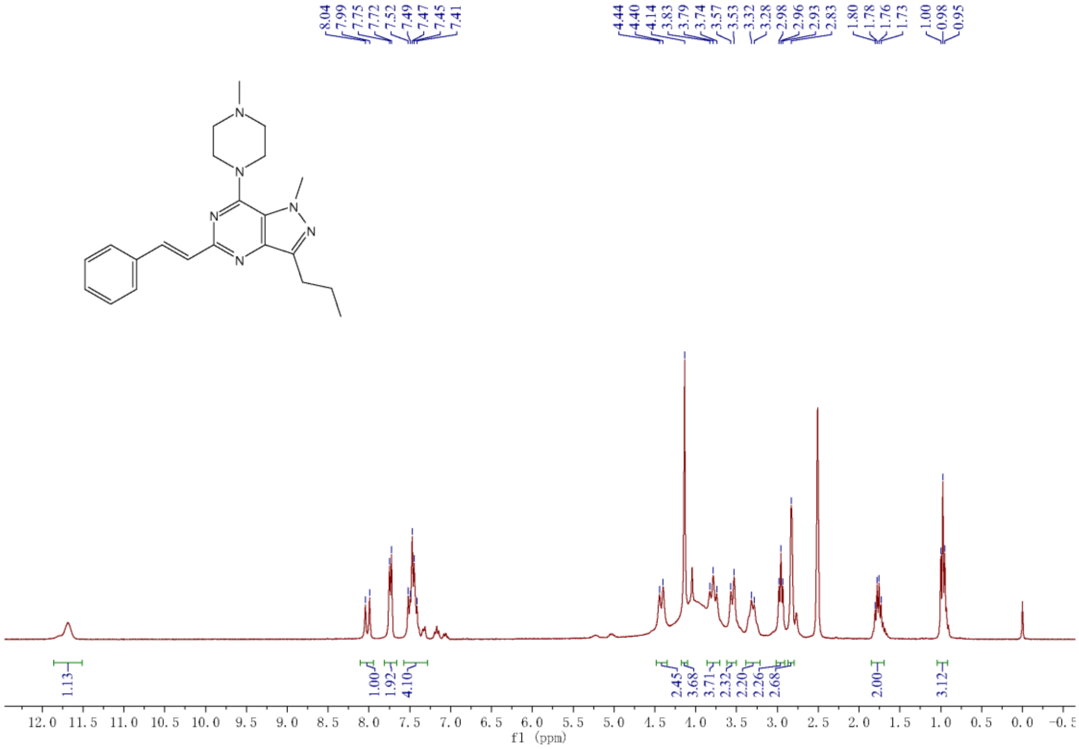

^{1}H NMR and ^{13}C NMR for *2g*

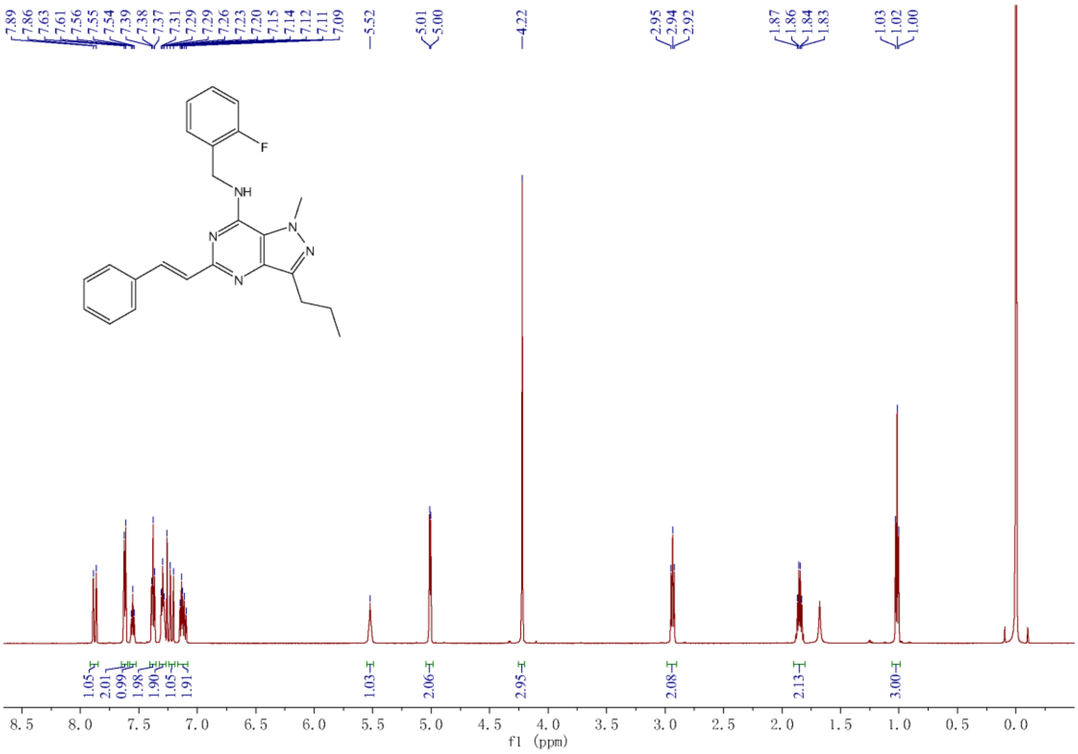

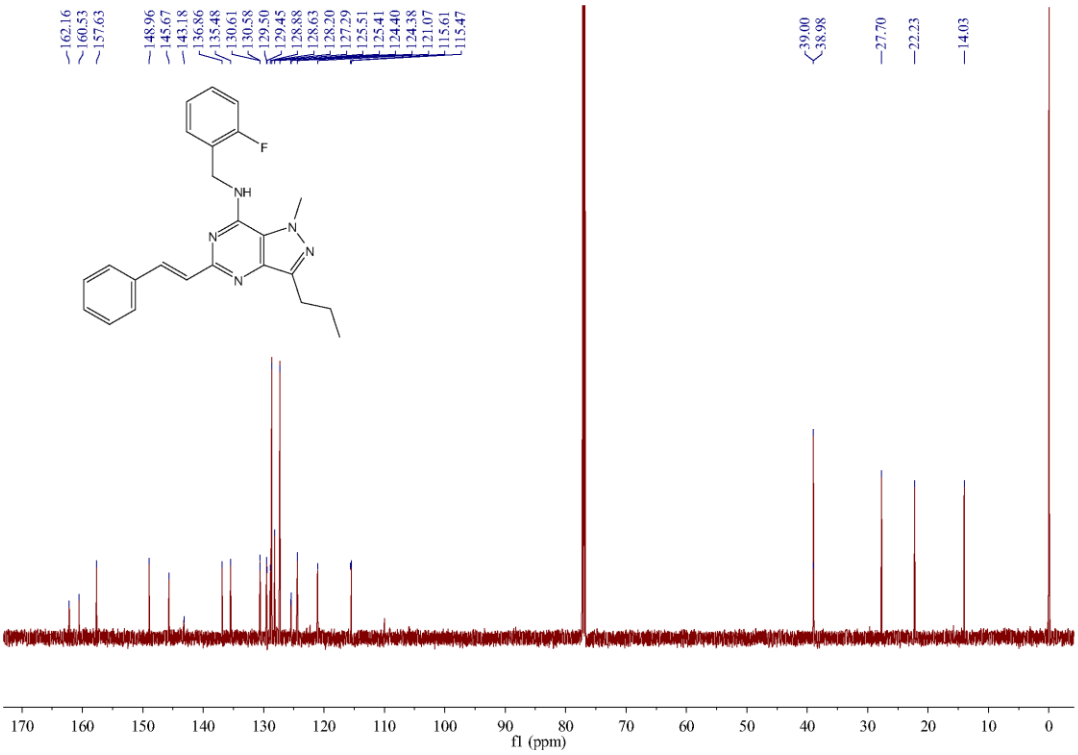

^{1}H NMRand ^{13}C NMR for *3a*

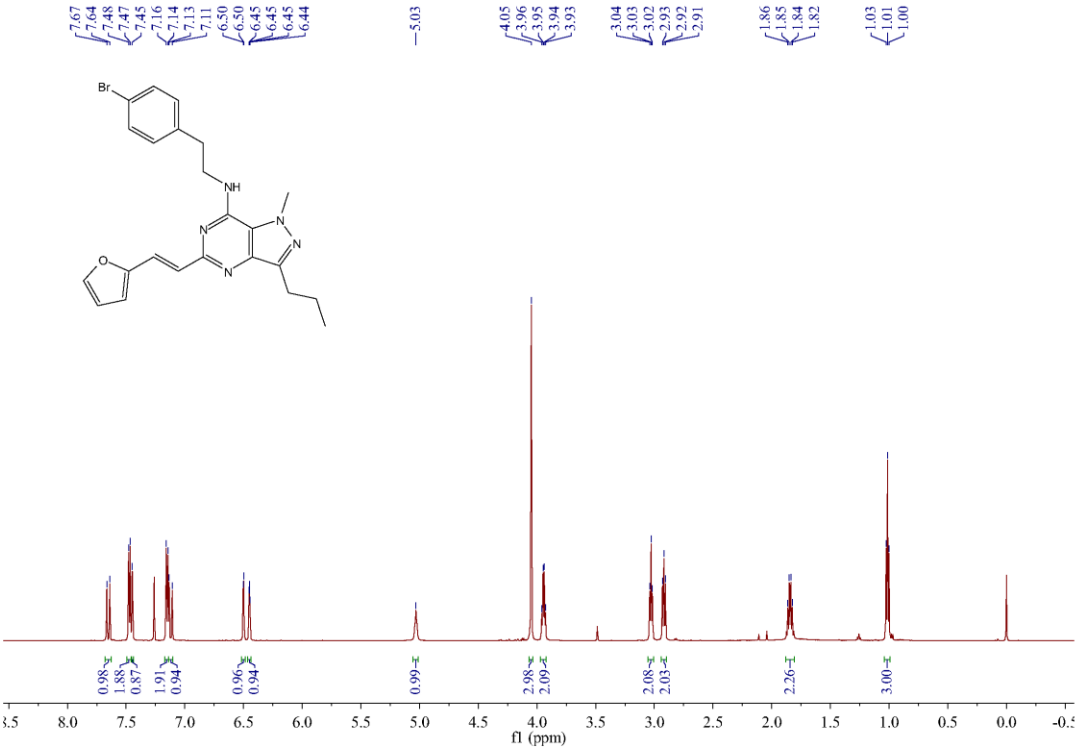

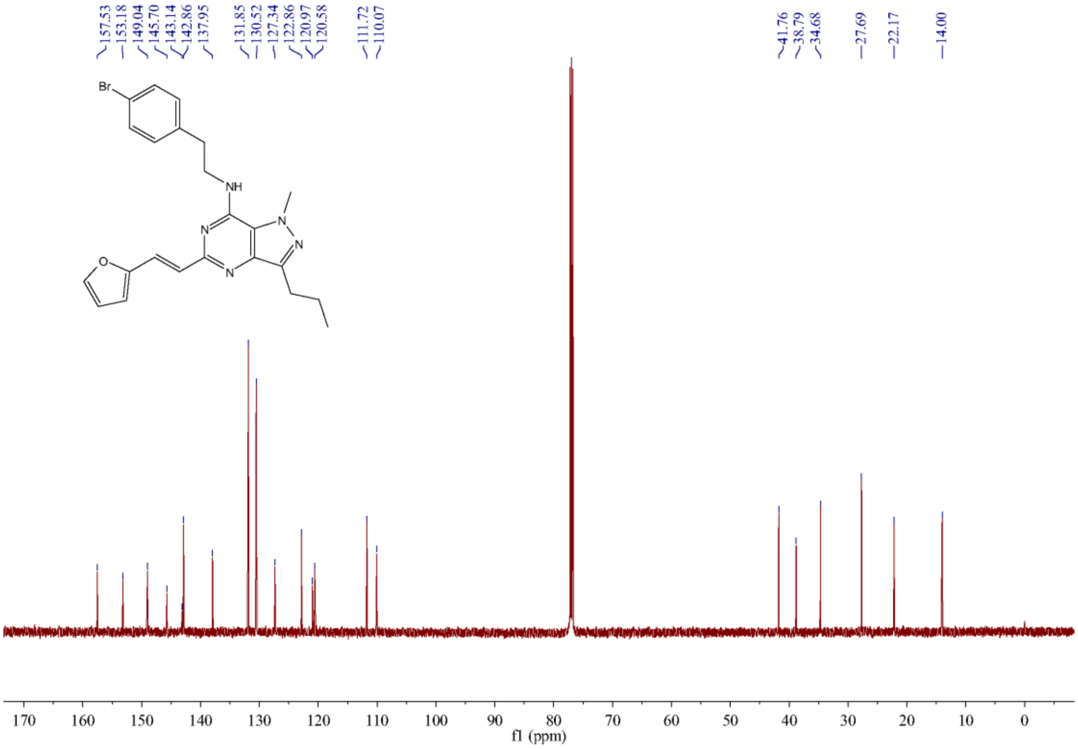

^{1}H NMR and ^{13}C NMR for *3b*

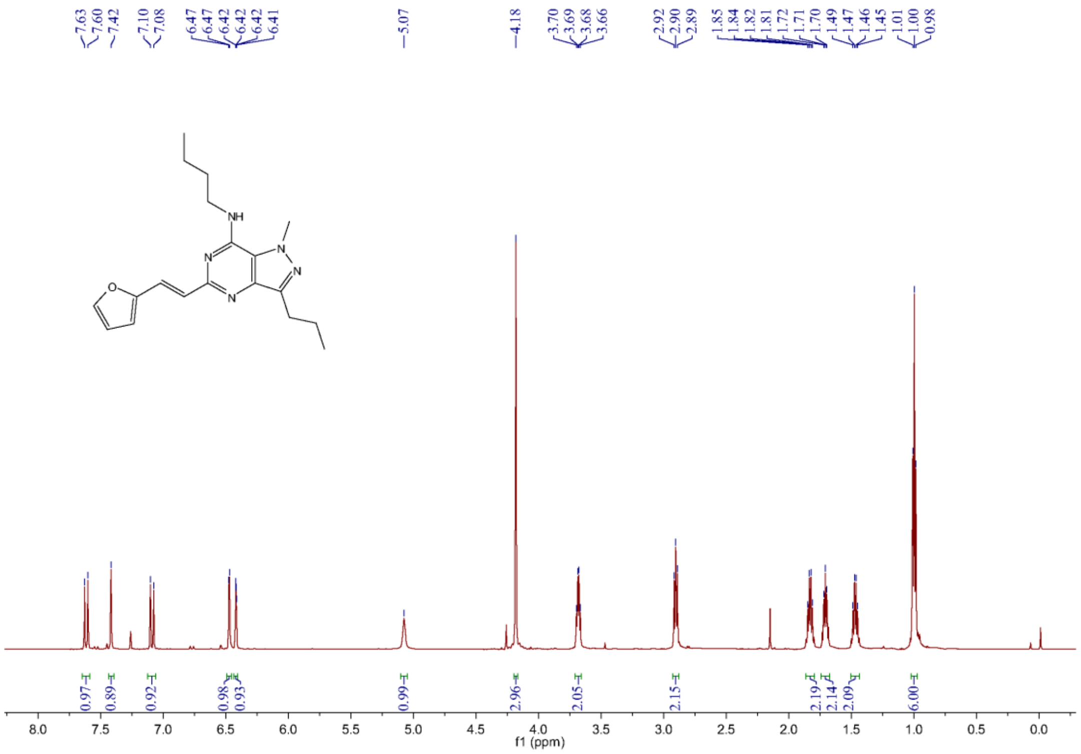

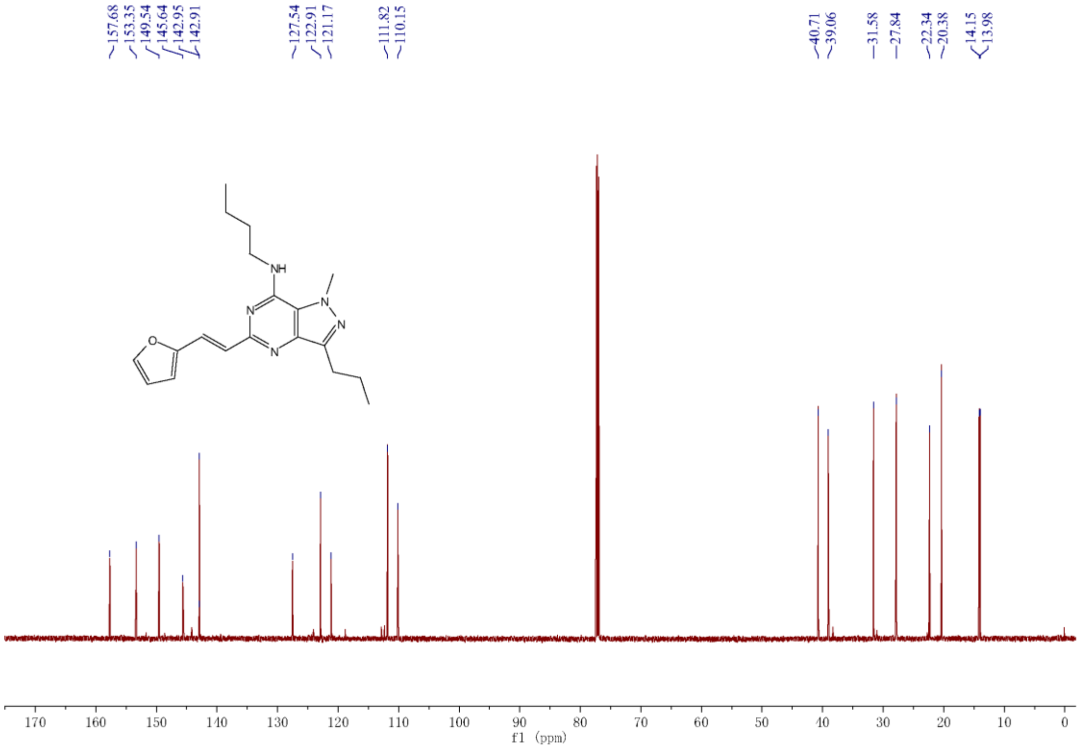

^{1}H NMR and ^{13}C NMR for *3c*

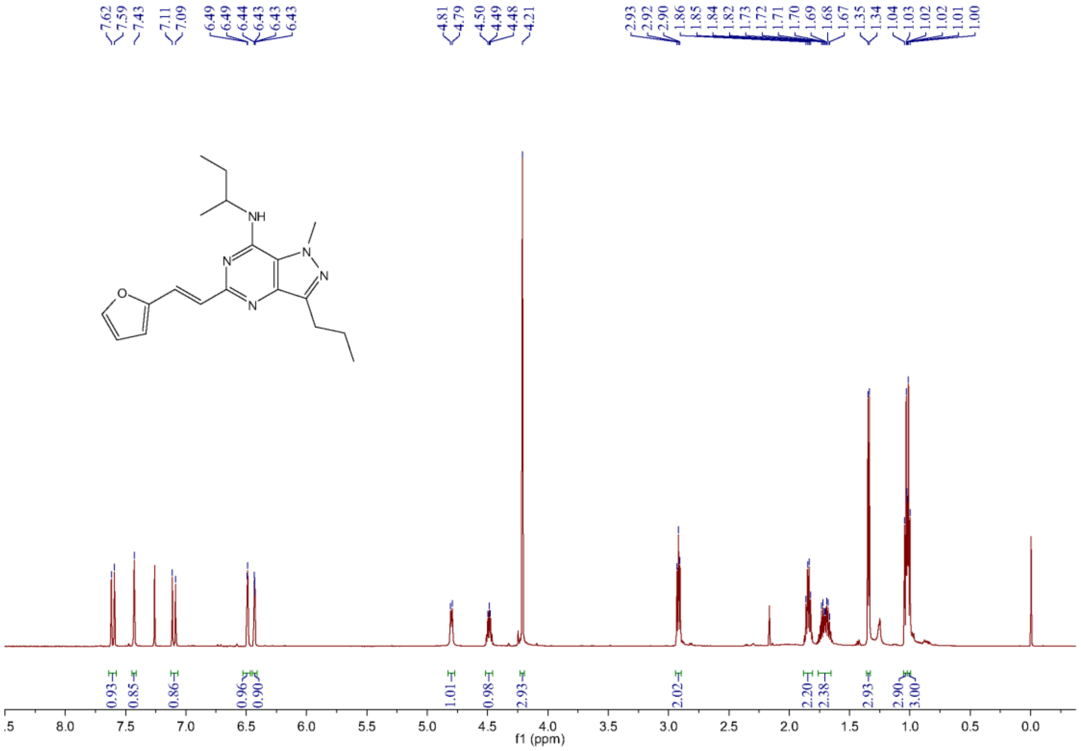

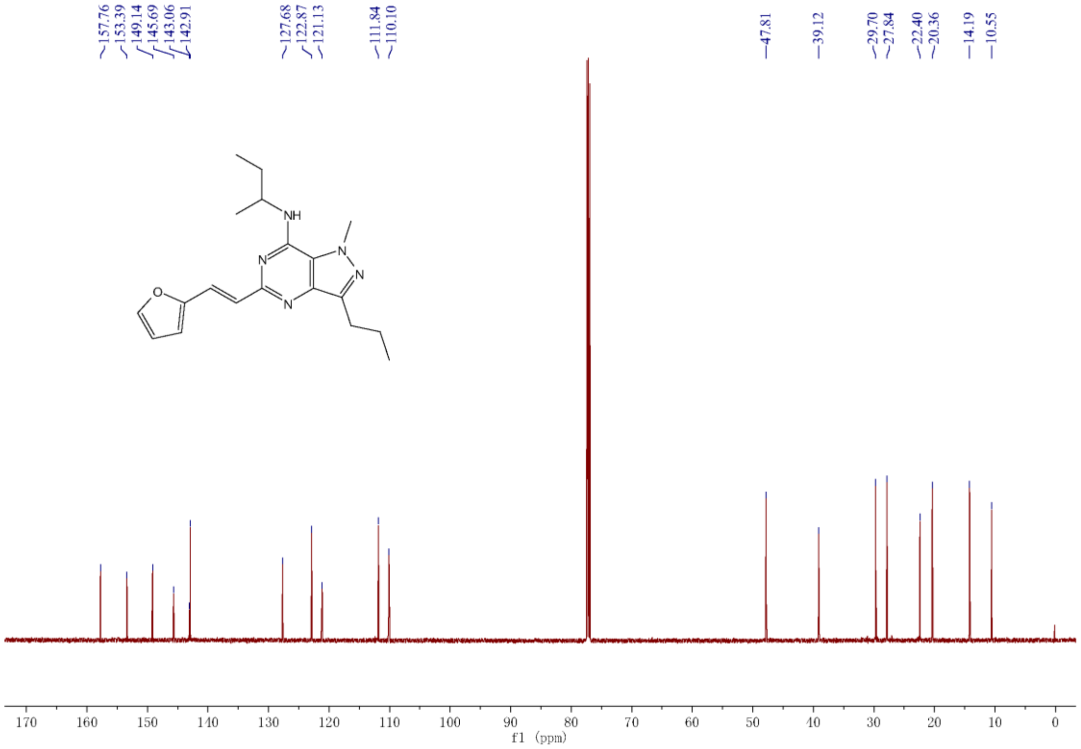

^{1}H NMR and ^{13}C NMR for *3d*

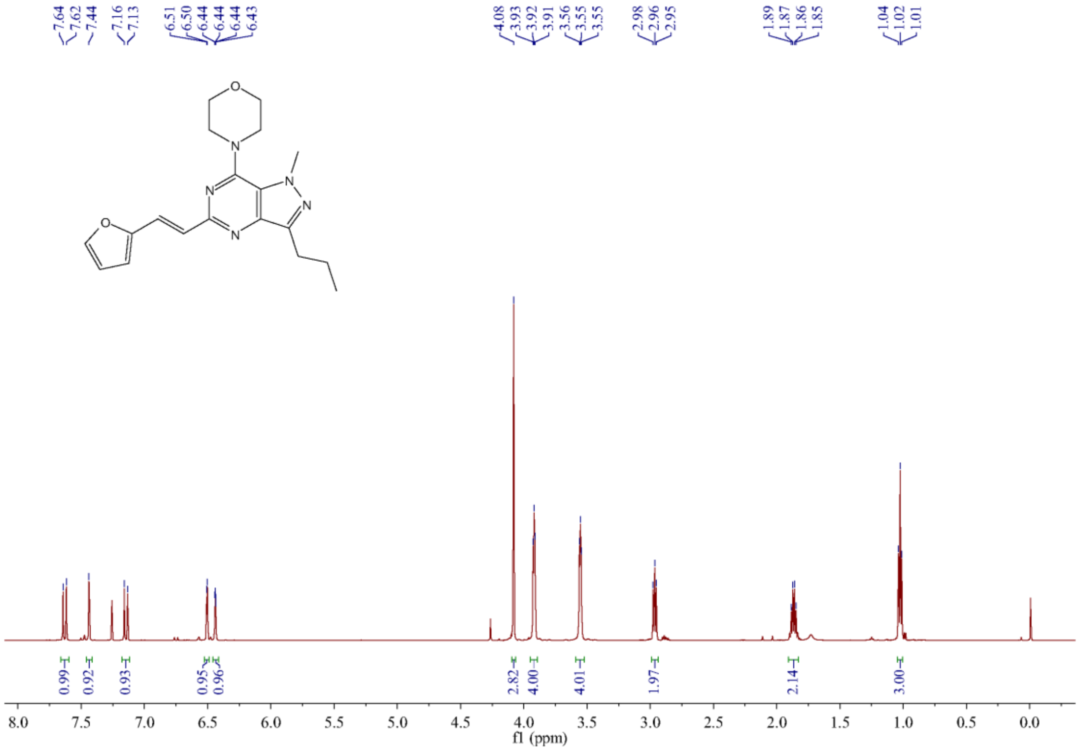

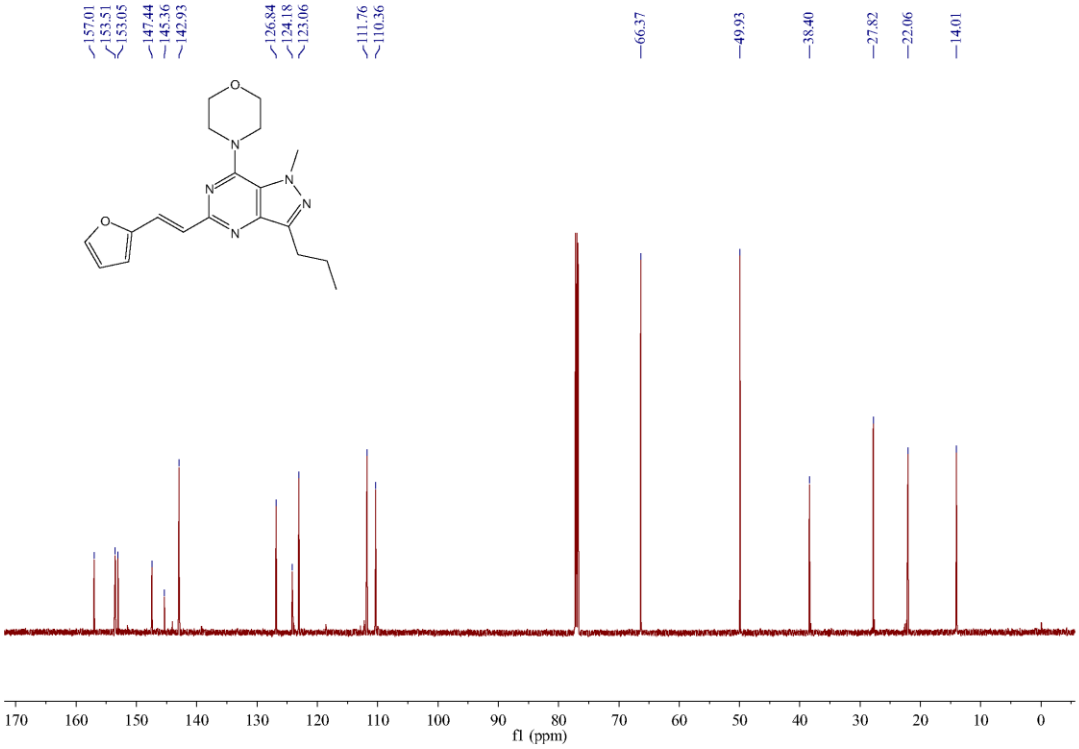

^{1}H NMR and ^{13}C NMR for *3e*

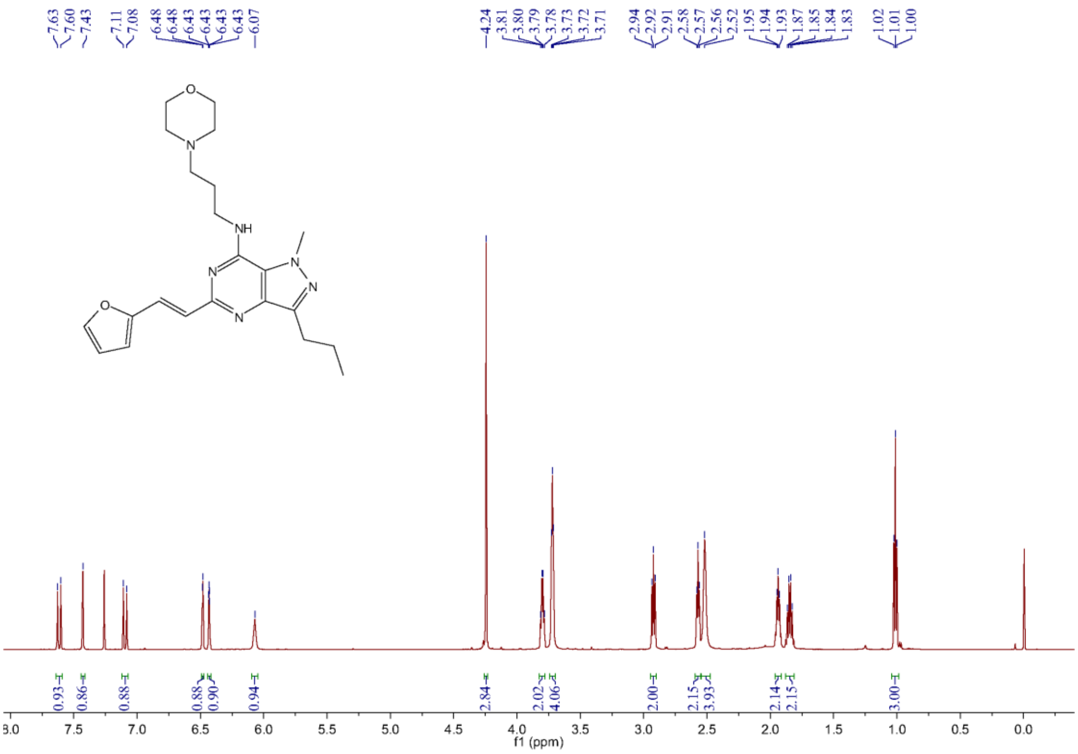

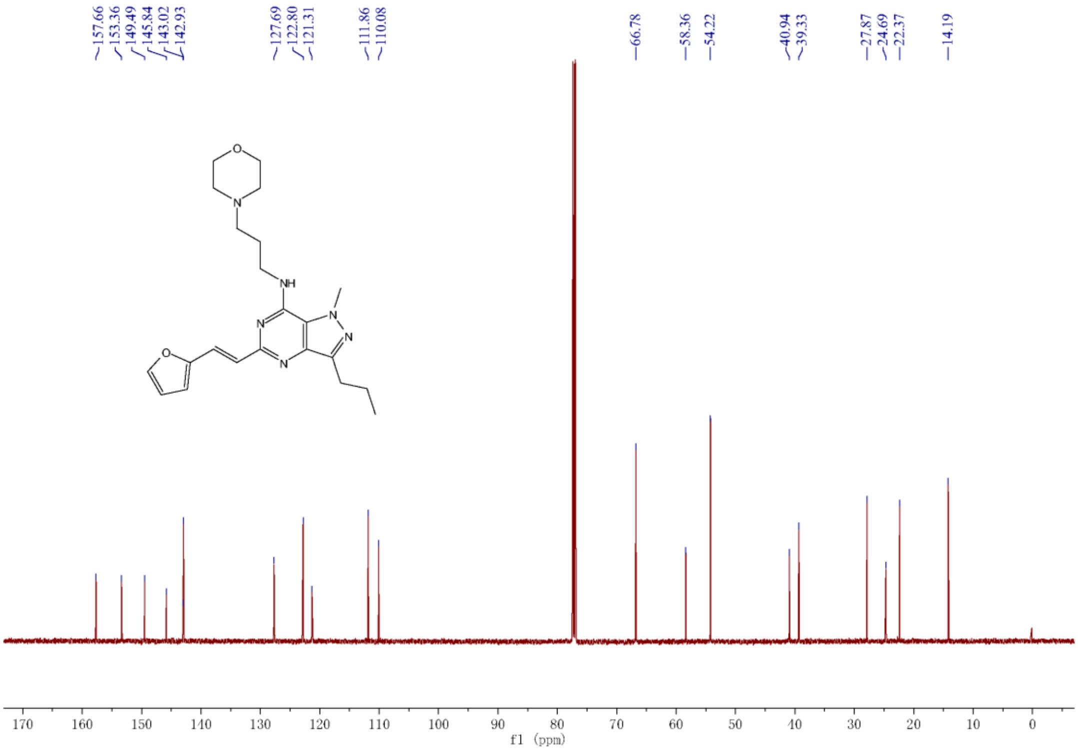

^{1}H NMR and ^{13}C NMR for *3f*

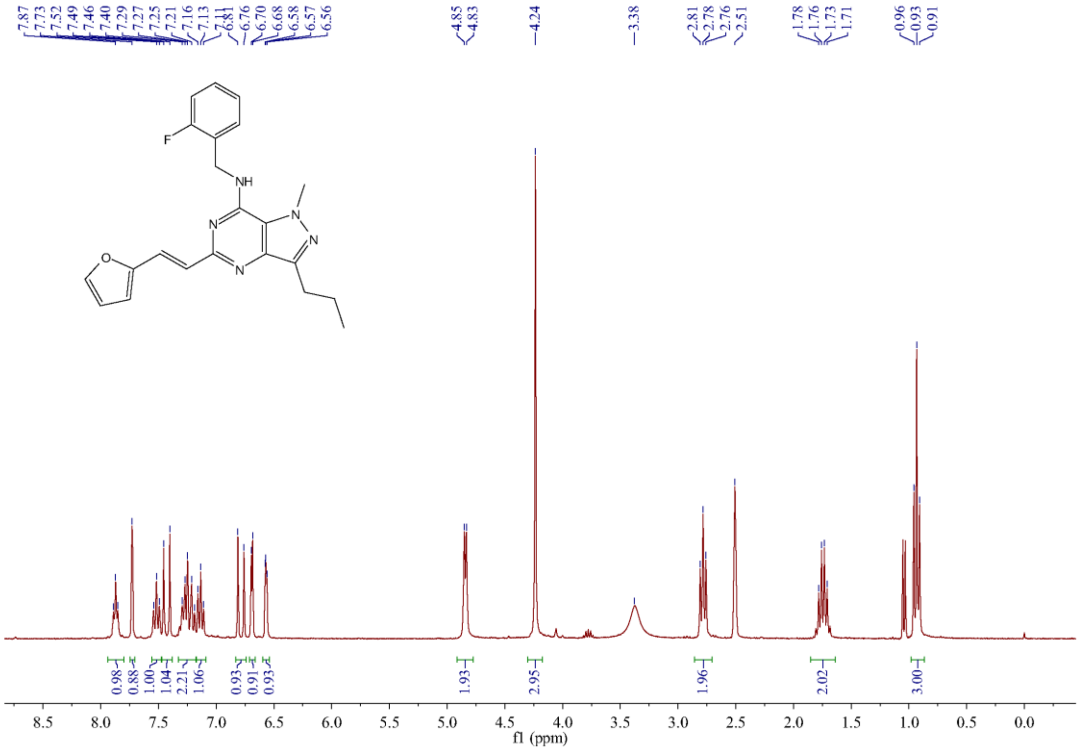

^{1}H NMR and ^{13}C NMR for *3g*

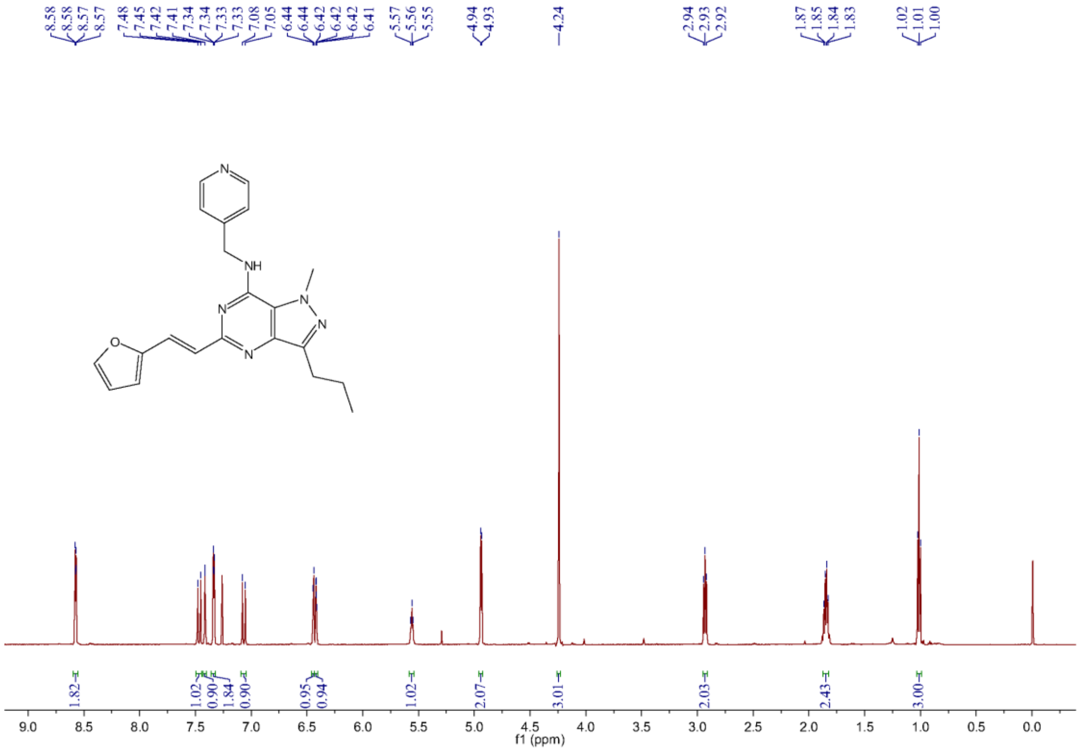

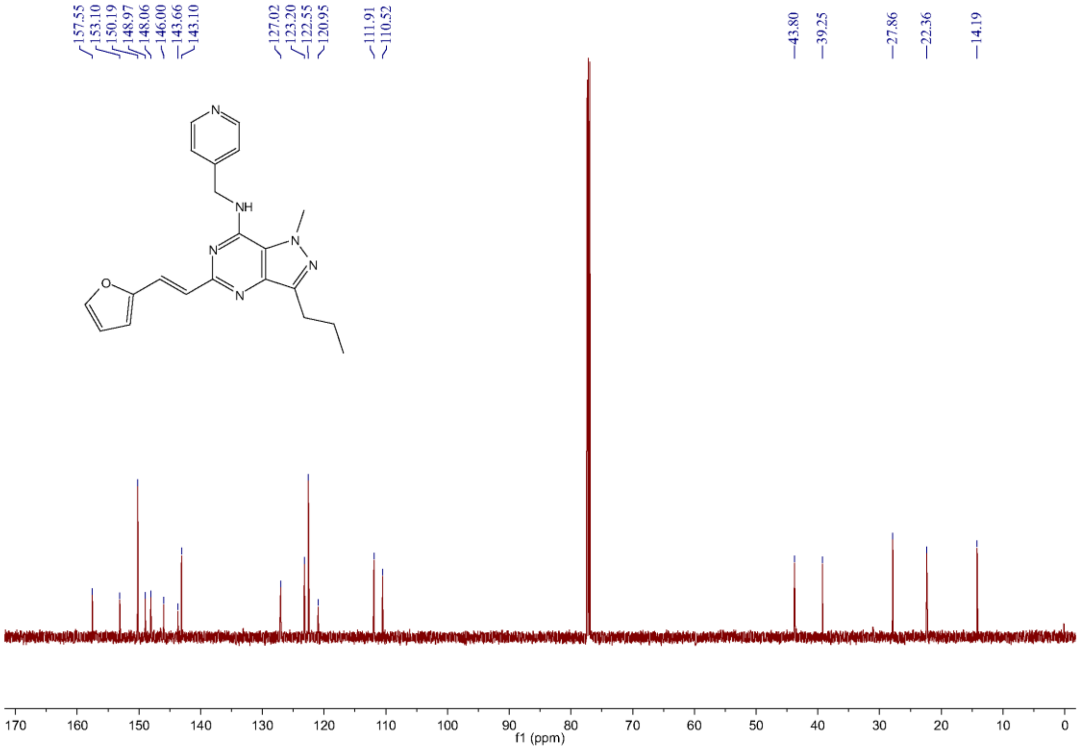

^{1}H NMR and ^{13}C NMR for *4a*

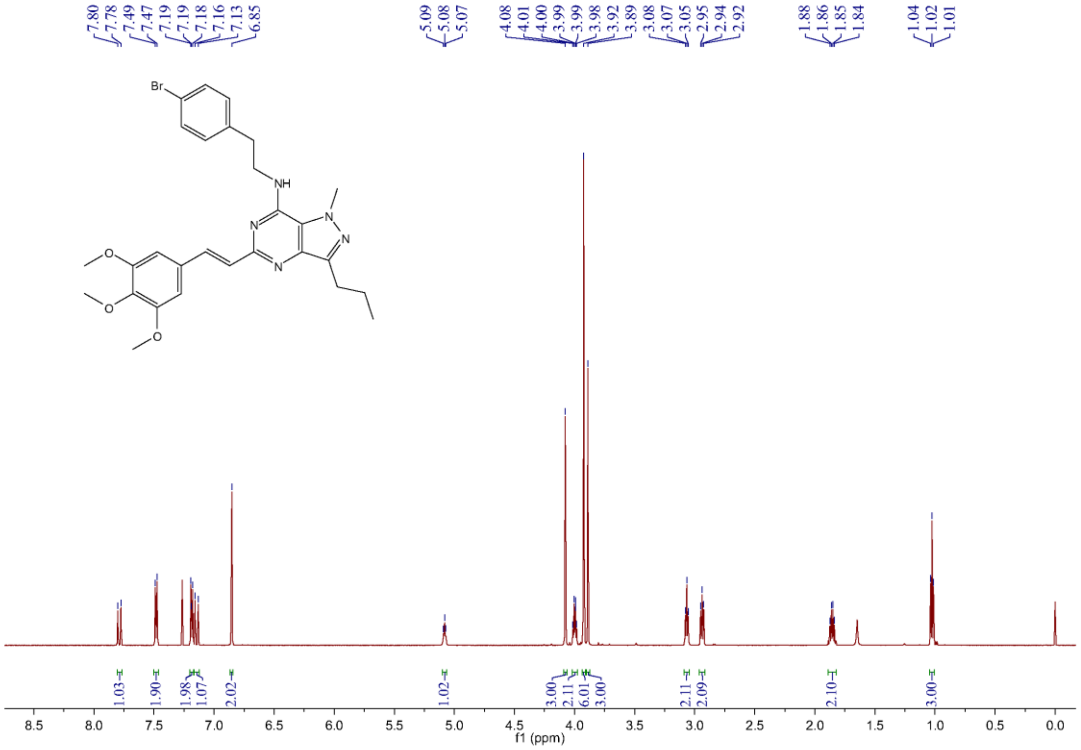

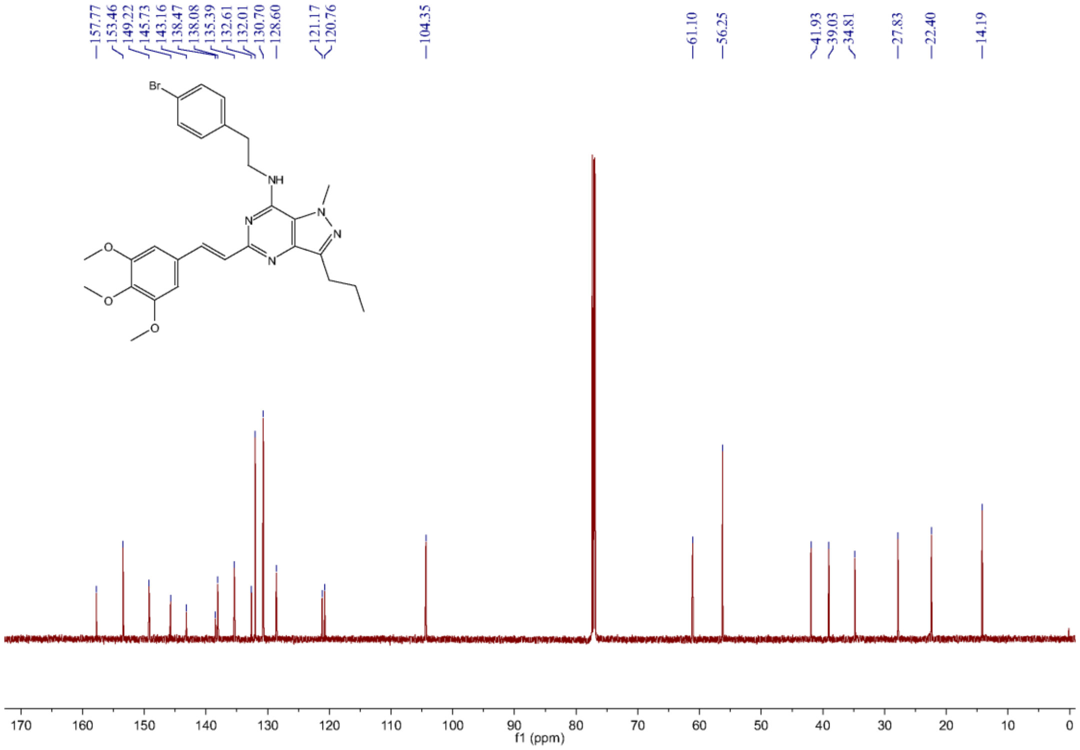

^{1}H NMR and ^{13}C NMR for *4b*

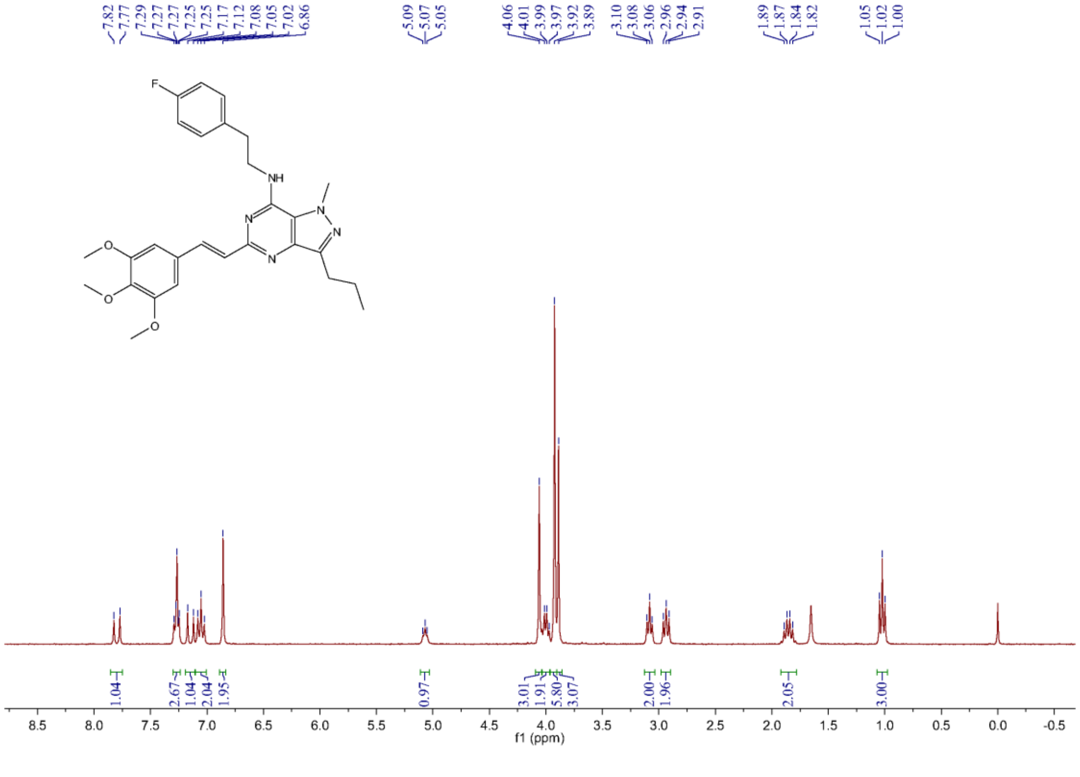

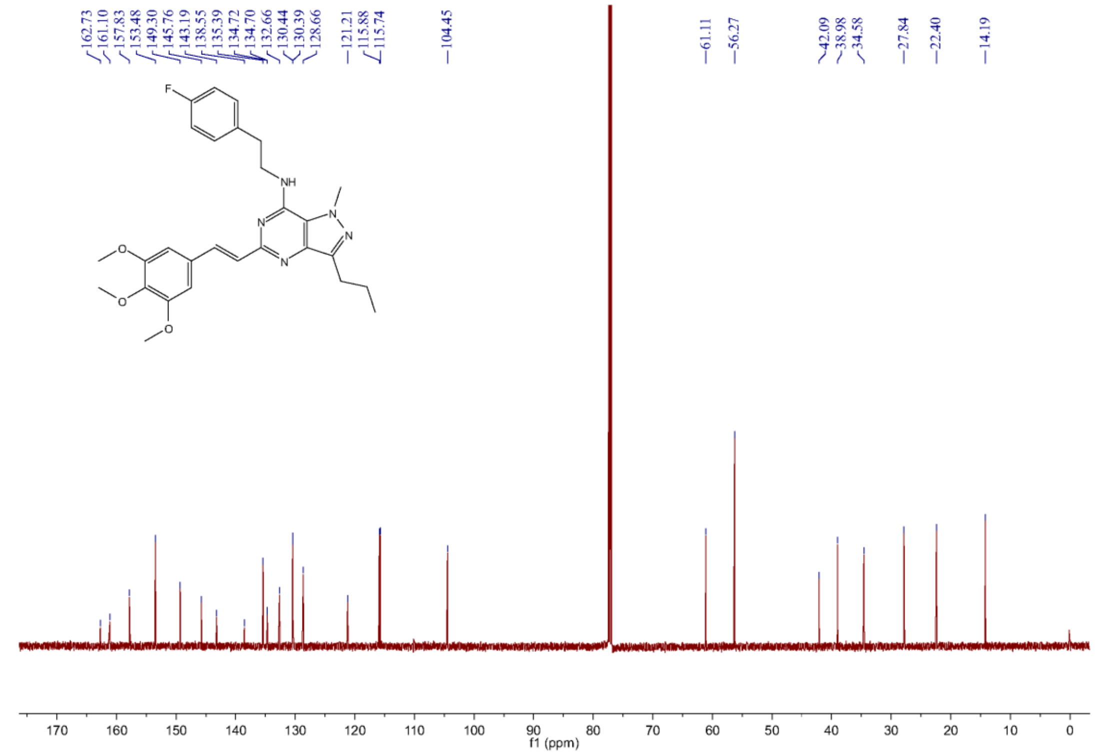

^{1}H NMR and ^{13}C NMR for *4c*

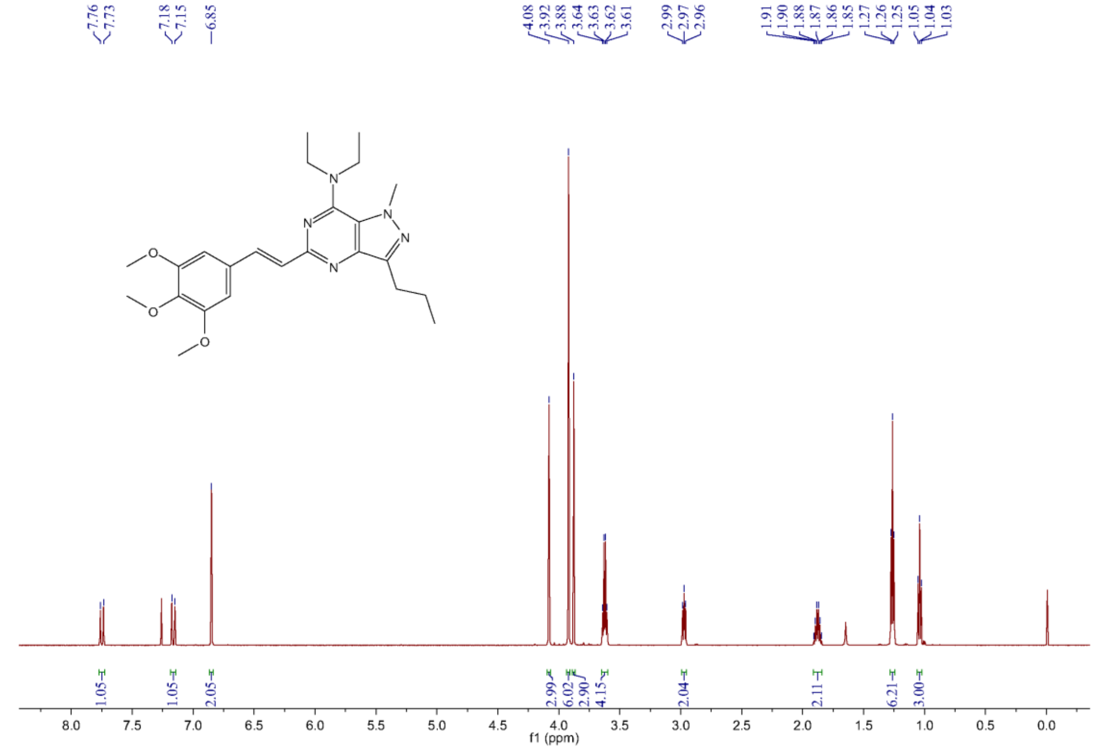

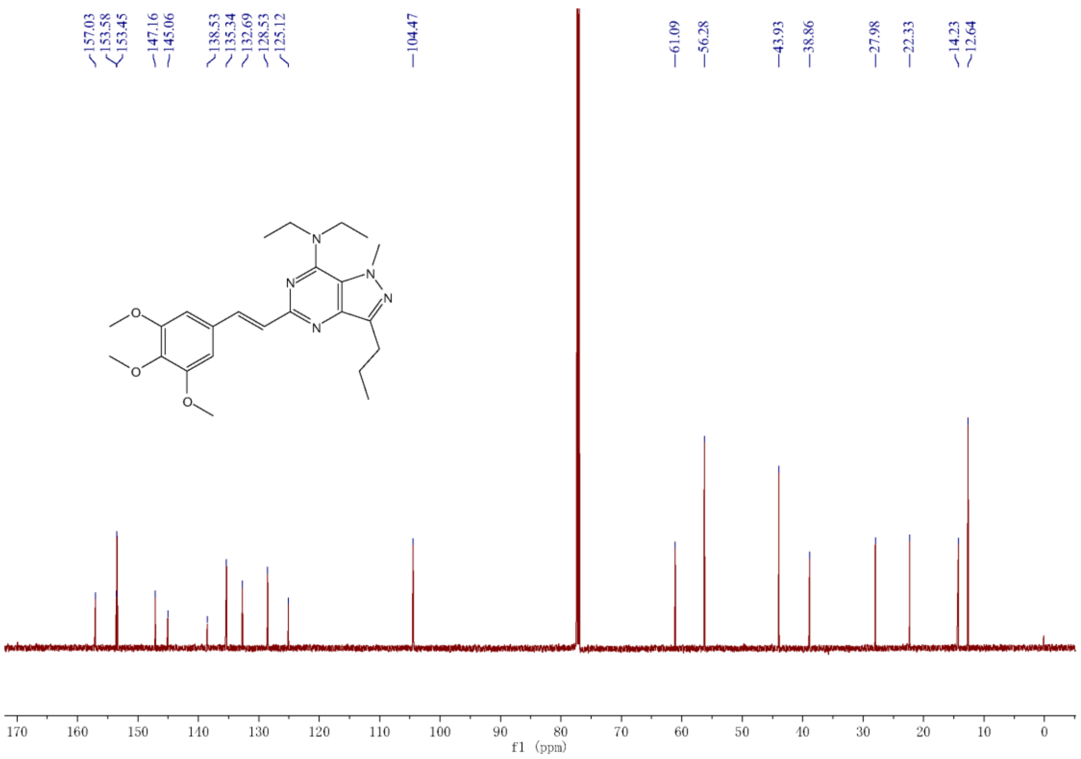

^{1}H NMR and ^{13}C NMR for *4d*

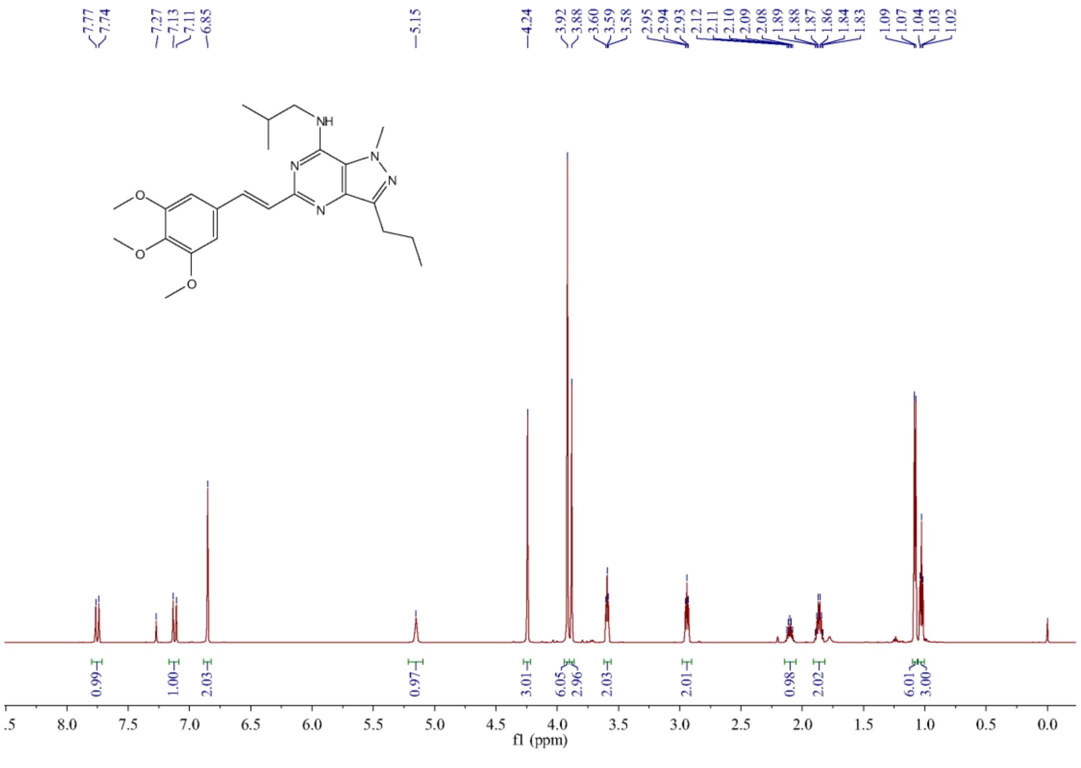

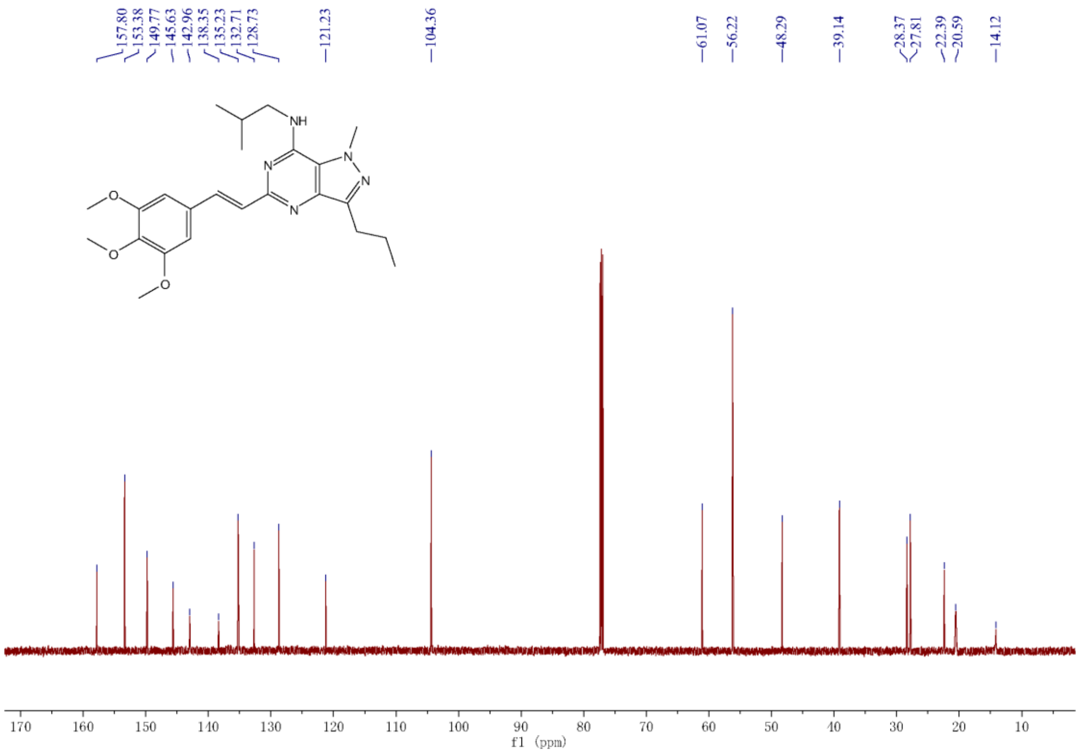

^{1}H NMR and ^{13}C NMR for *4e*

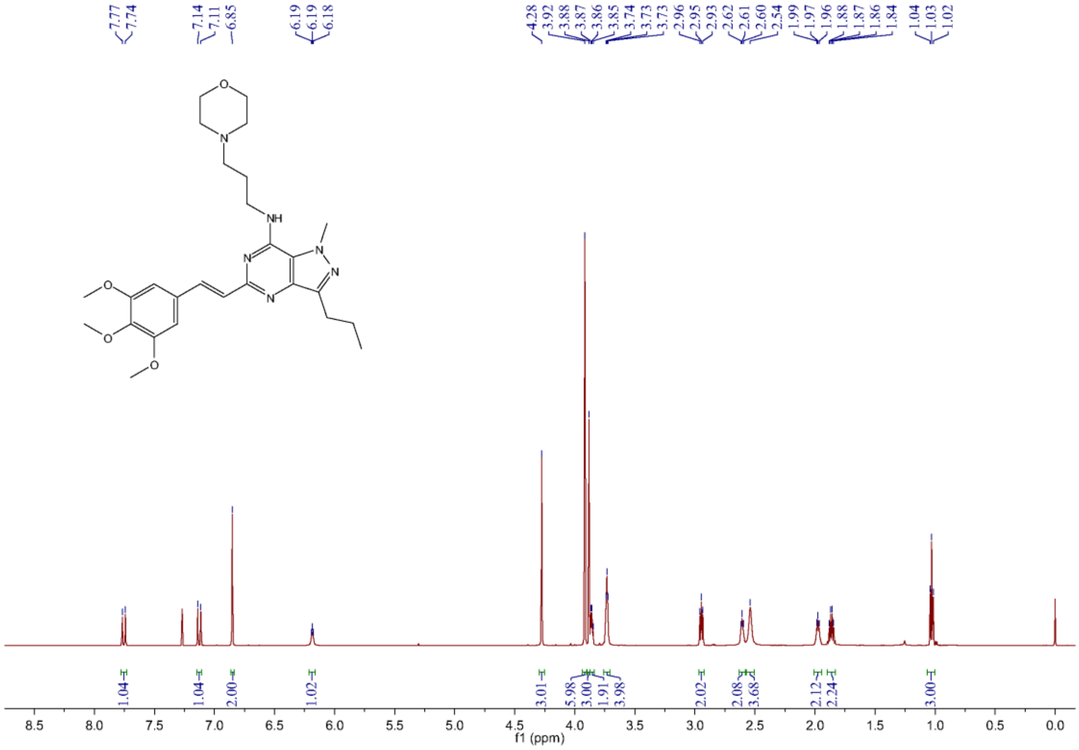

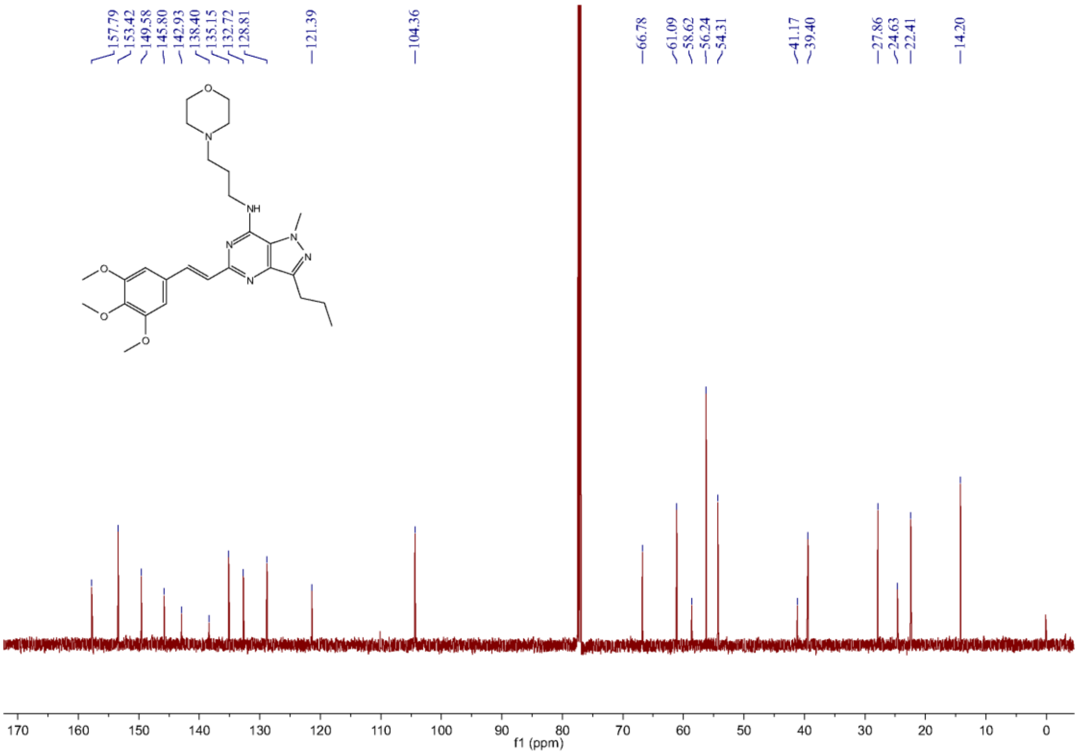

^{1}H NMR and ^{13}C NMR for *4f*

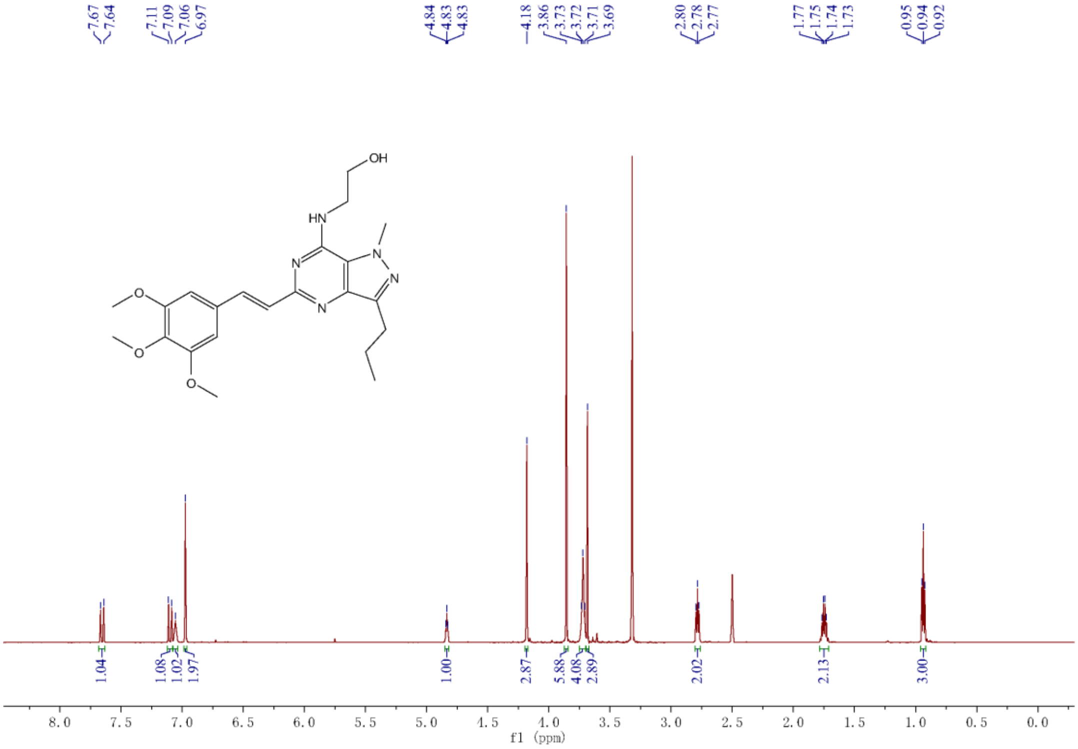

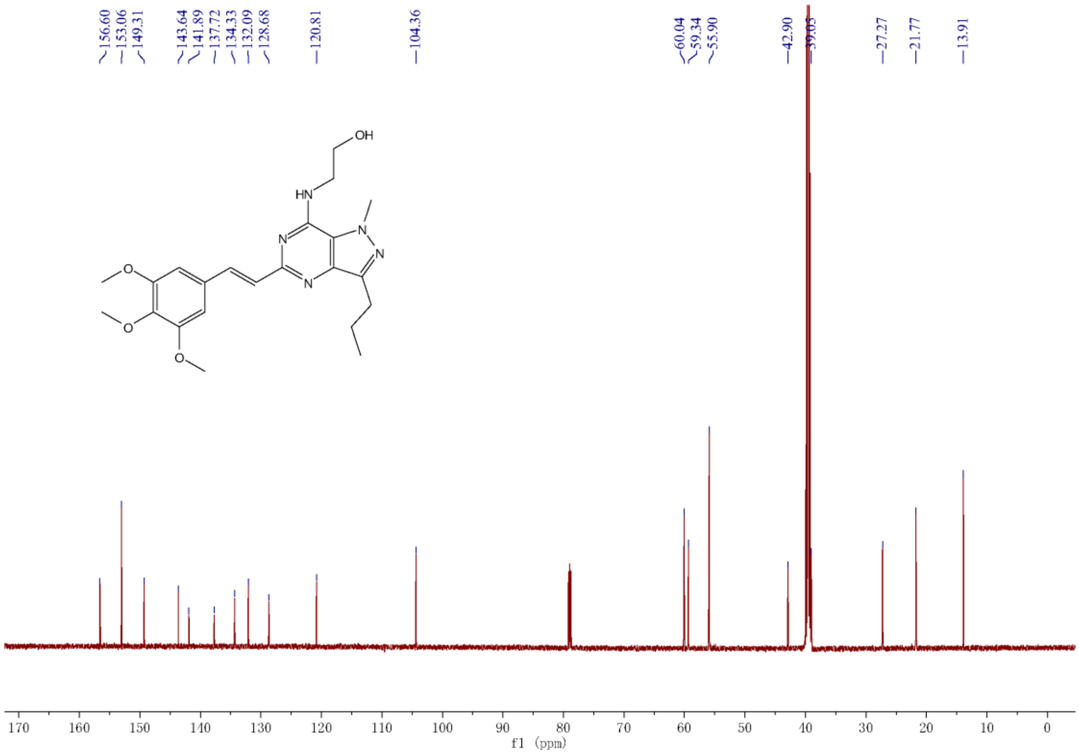

^{1}H NMR and ^{13}C NMR for *4g*

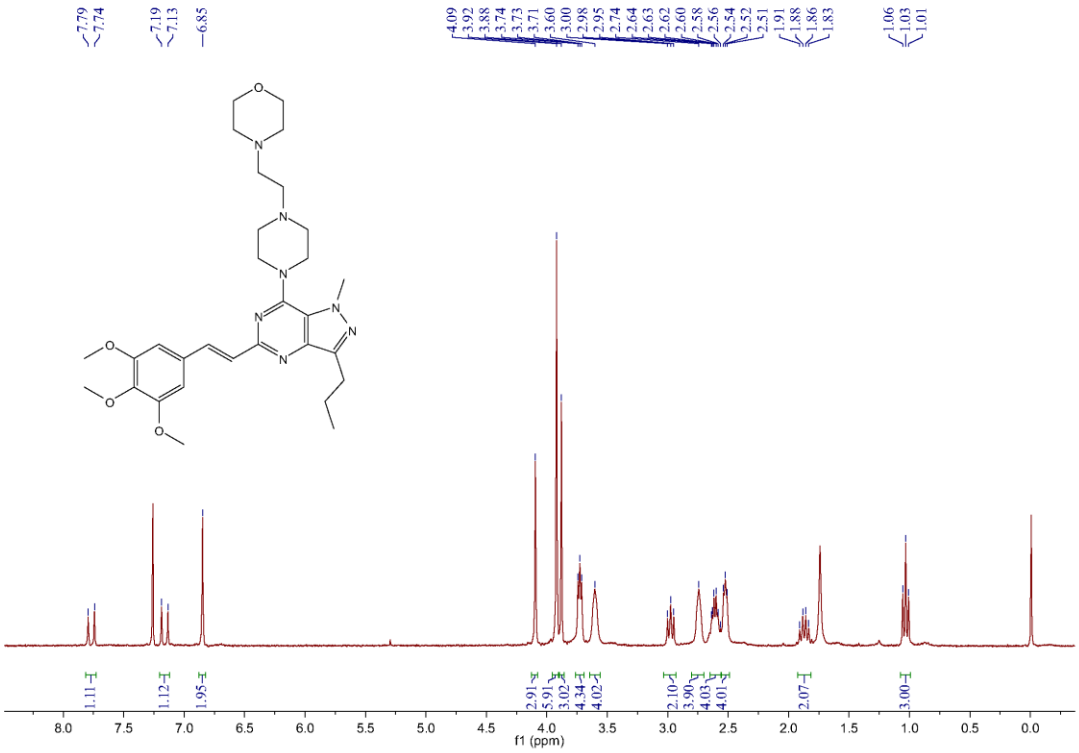

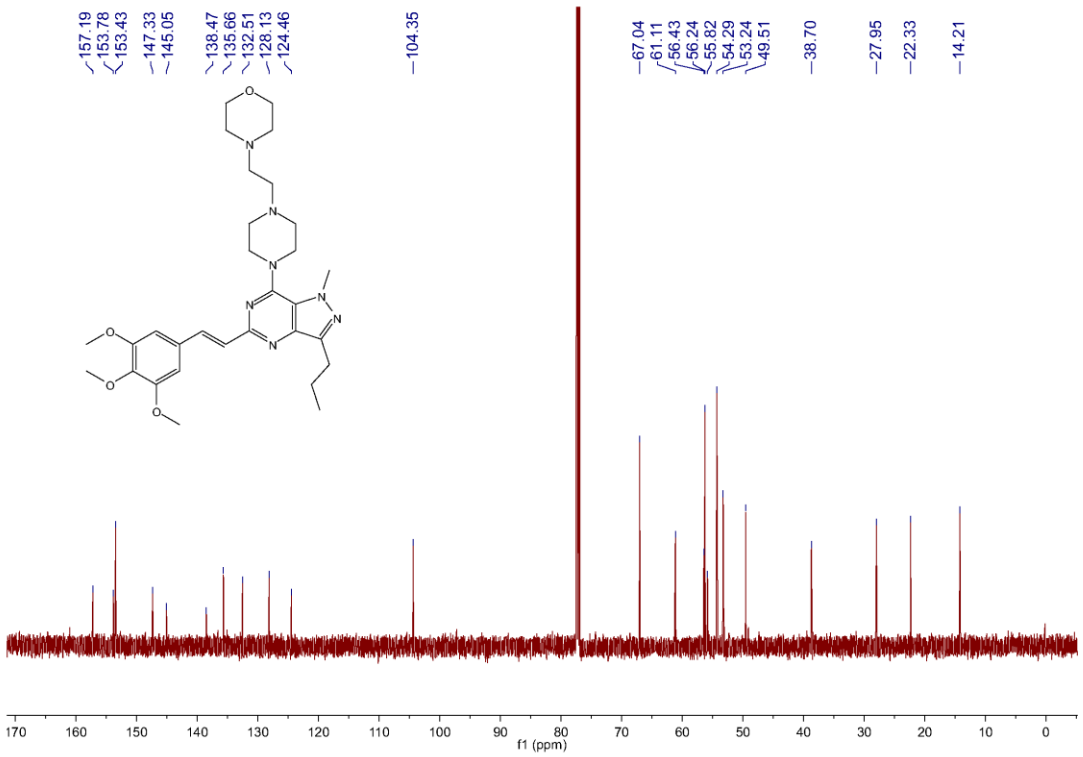

^{1}H NMR and ^{13}C NMR for *4h*

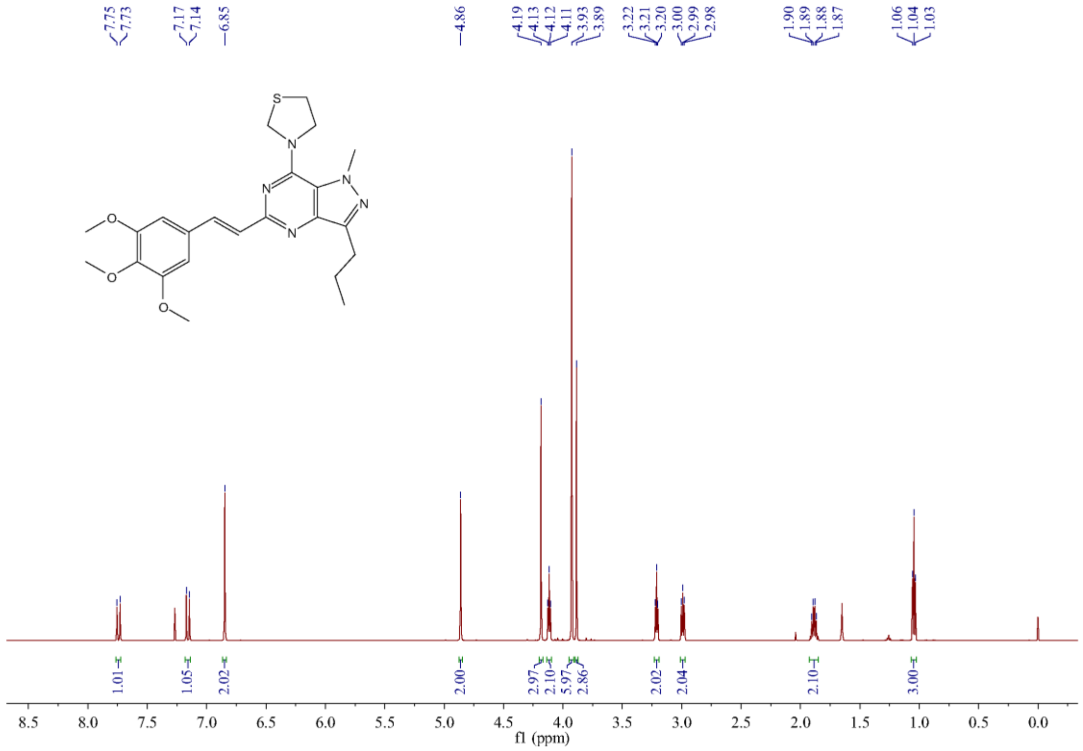

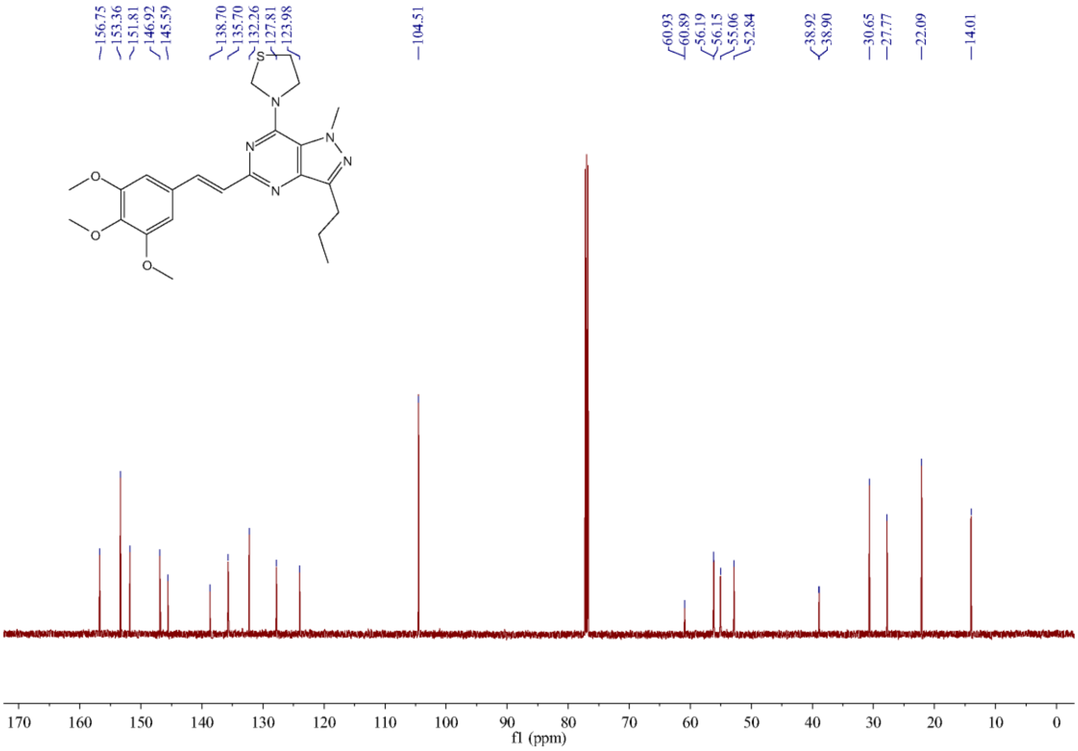

^{1}H NMR and ^{13}C NMR for *4i*

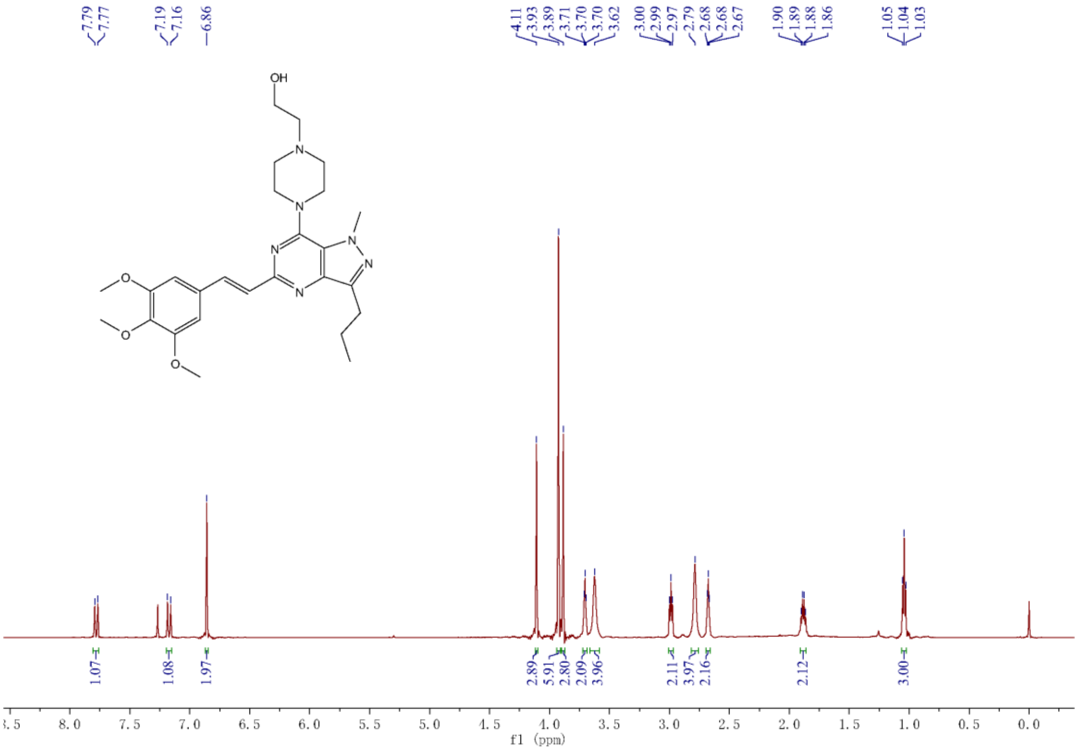

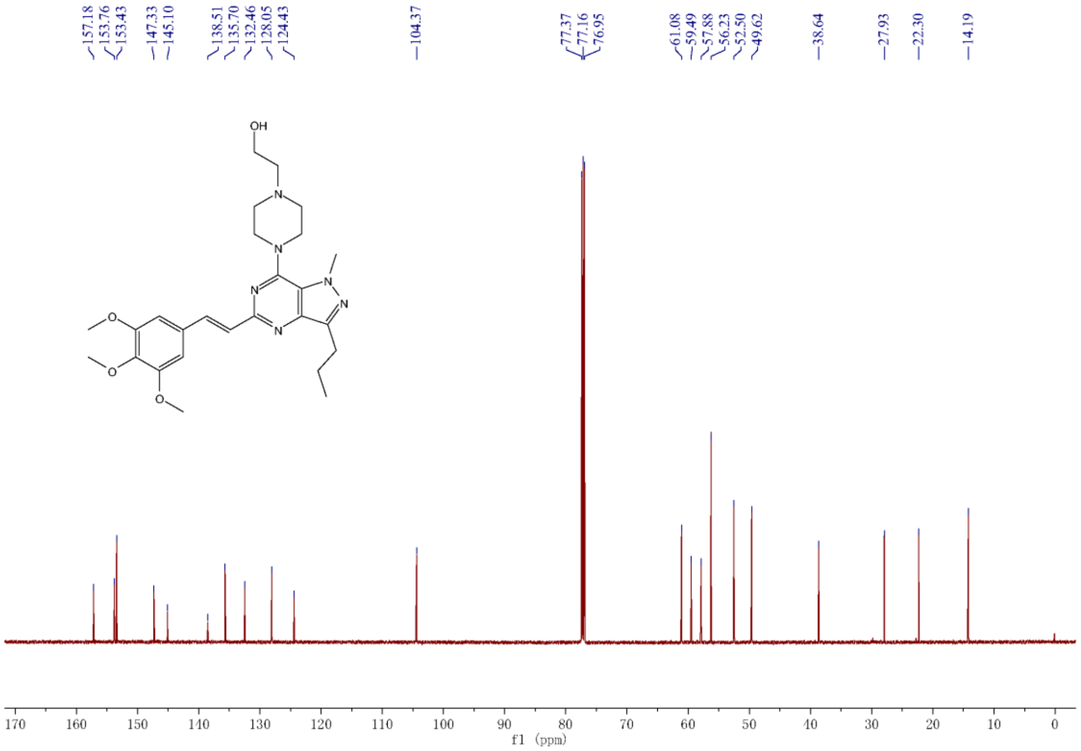

^{1}H NMR and ^{13}C NMR for *4j*

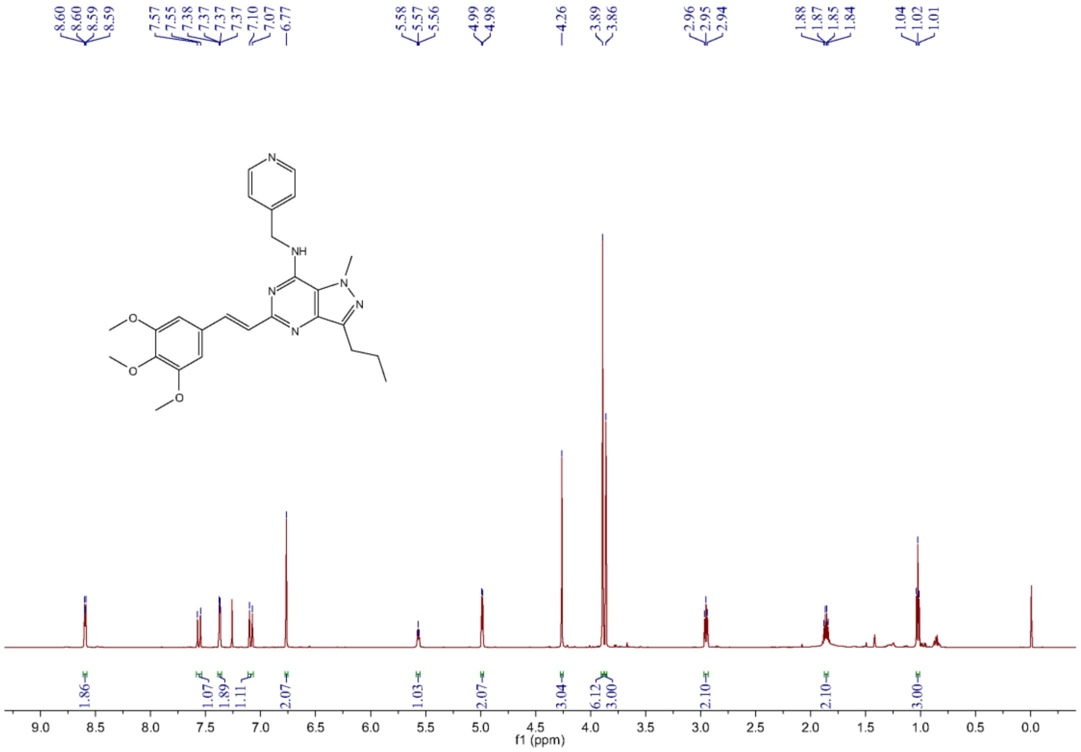

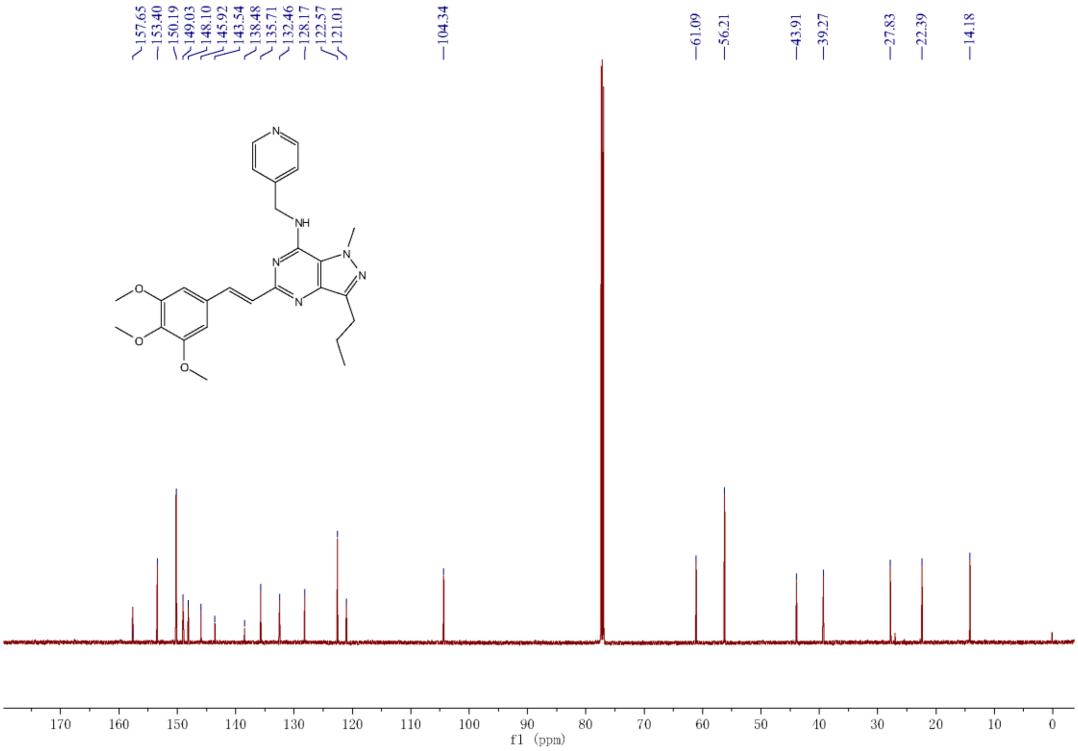

^{1}H NMR and ^{13}C NMR for *4k*

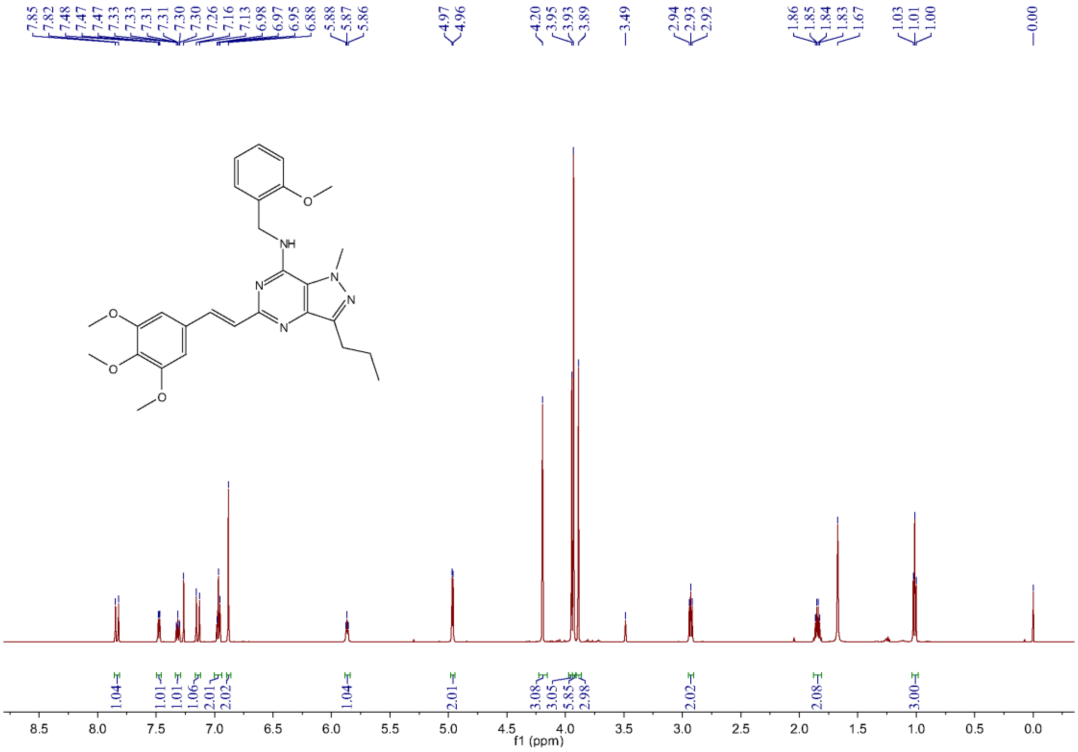

PART 3. HR-MS data of *1a~1j, 2a~2g, 3a~3g and 4a~4k*.

HR-MS data for *1a*

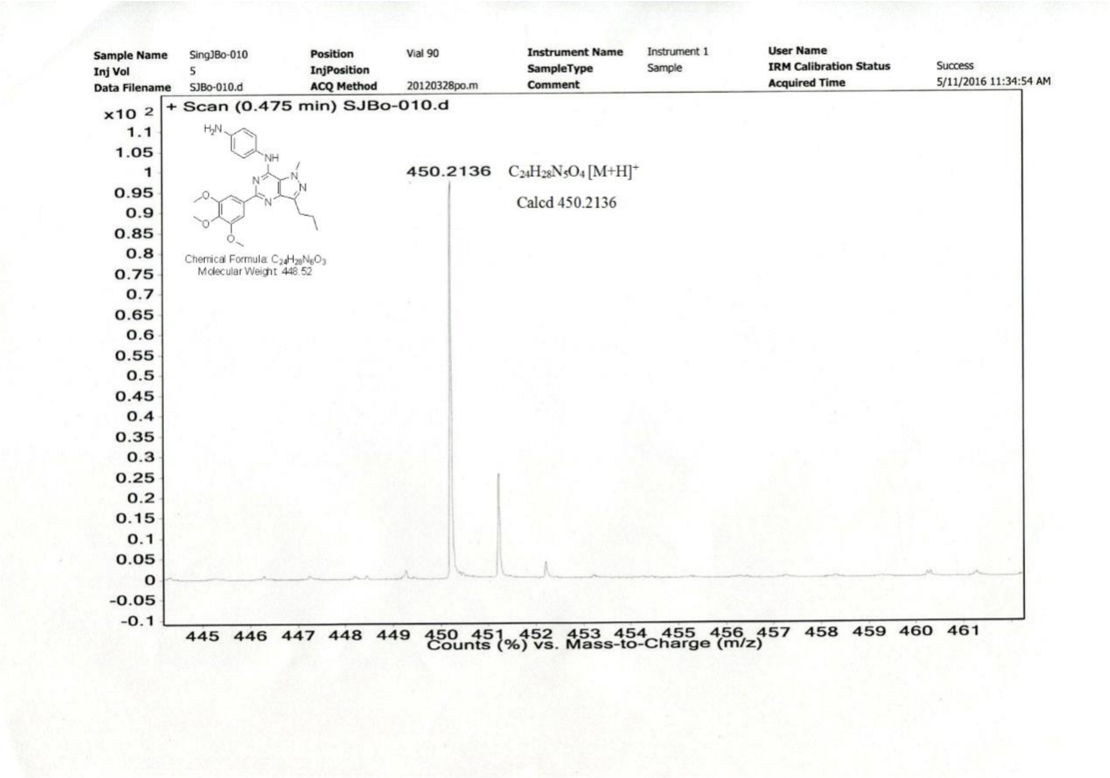

HR-MS data for *1b*

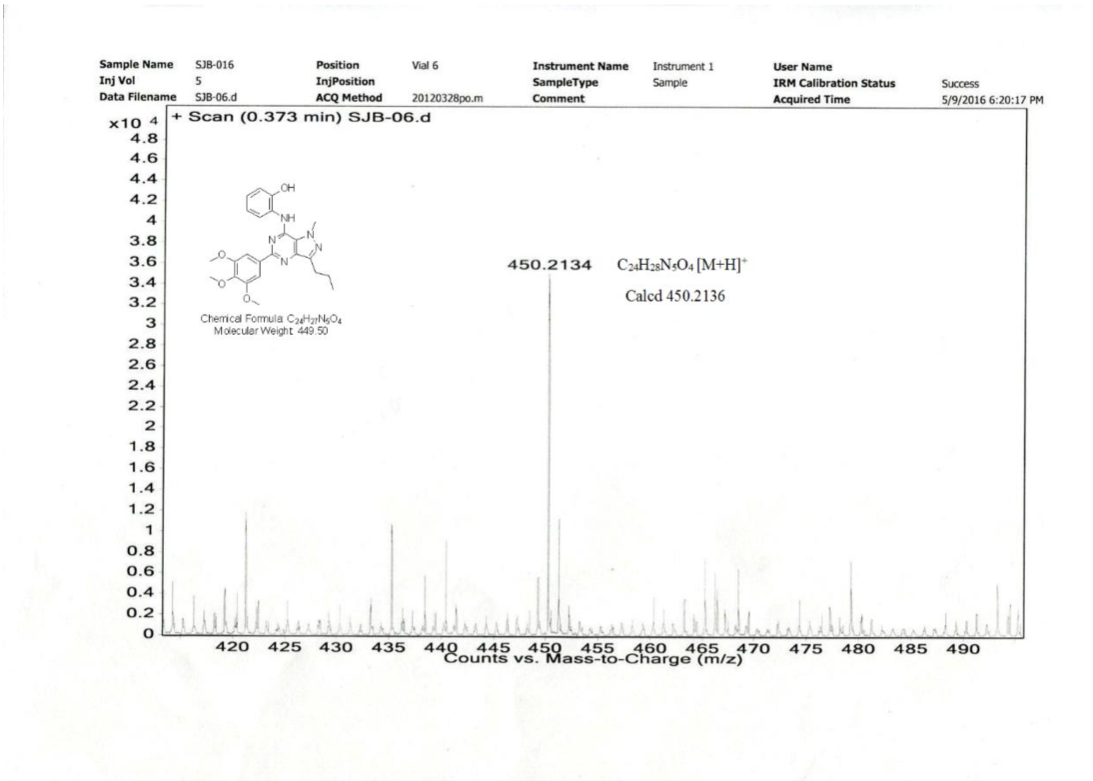

HR-MS data for *1c*

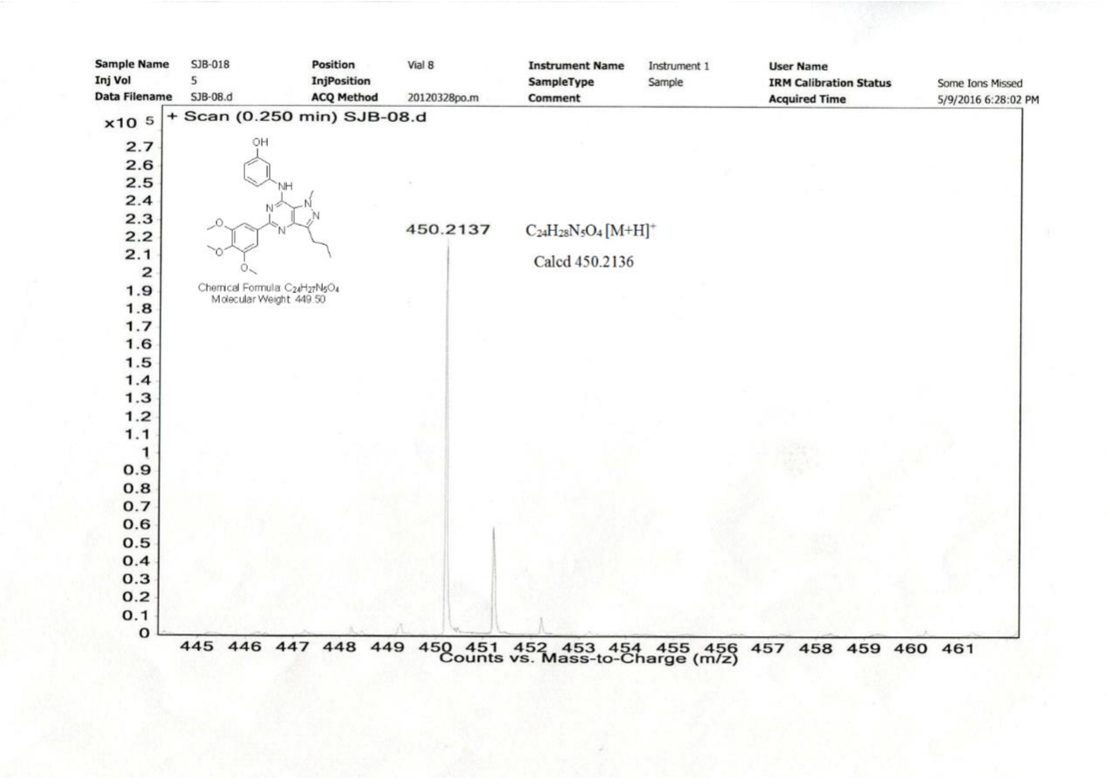

HR-MS data for *1d*

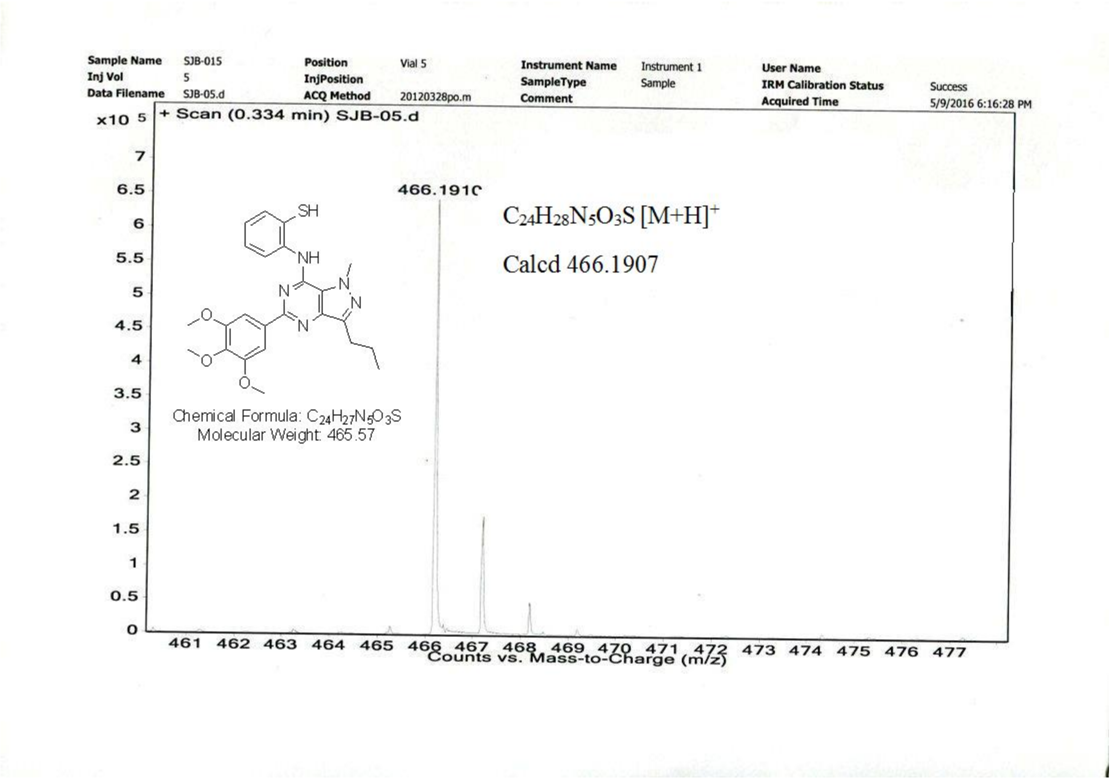

HR-MS data for *1e*

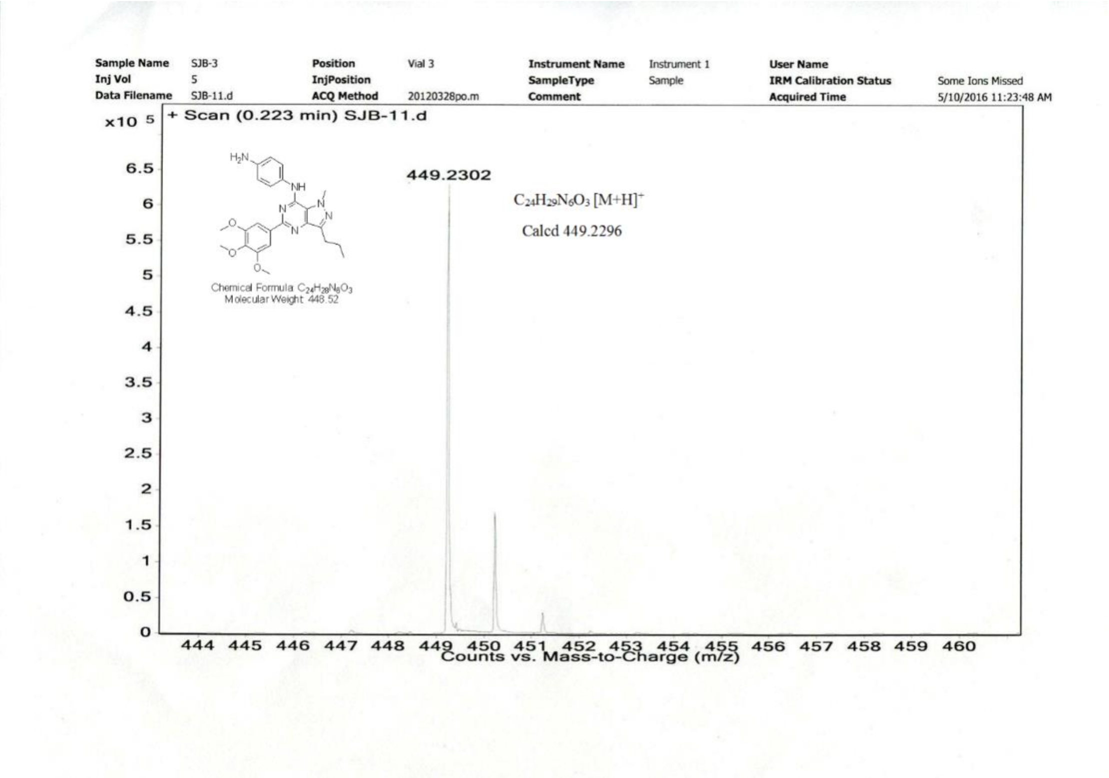

HR-MS data for *1f*

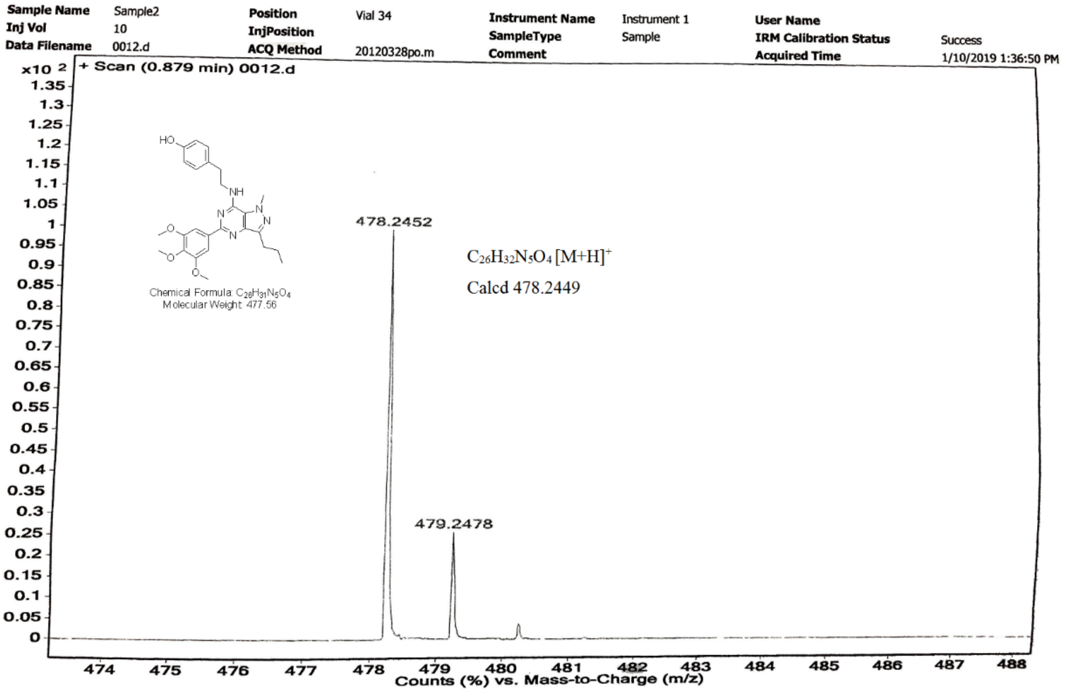

HR-MS data for *1g*

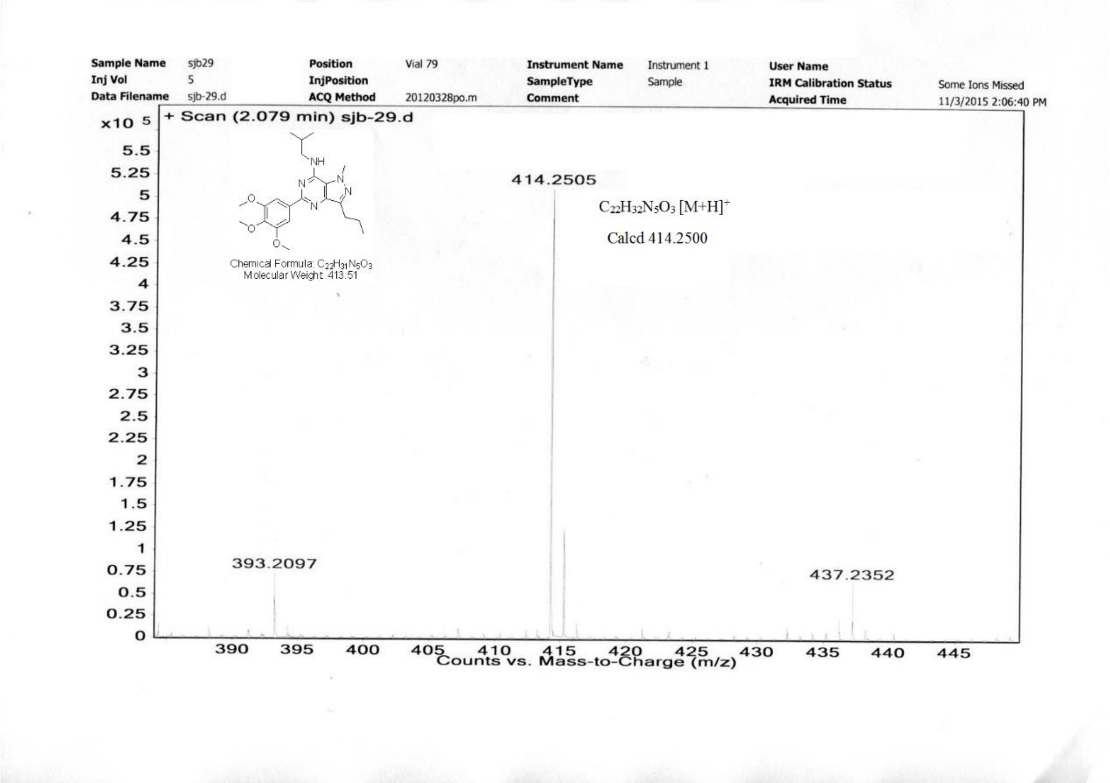

HR-MS data for *1h*

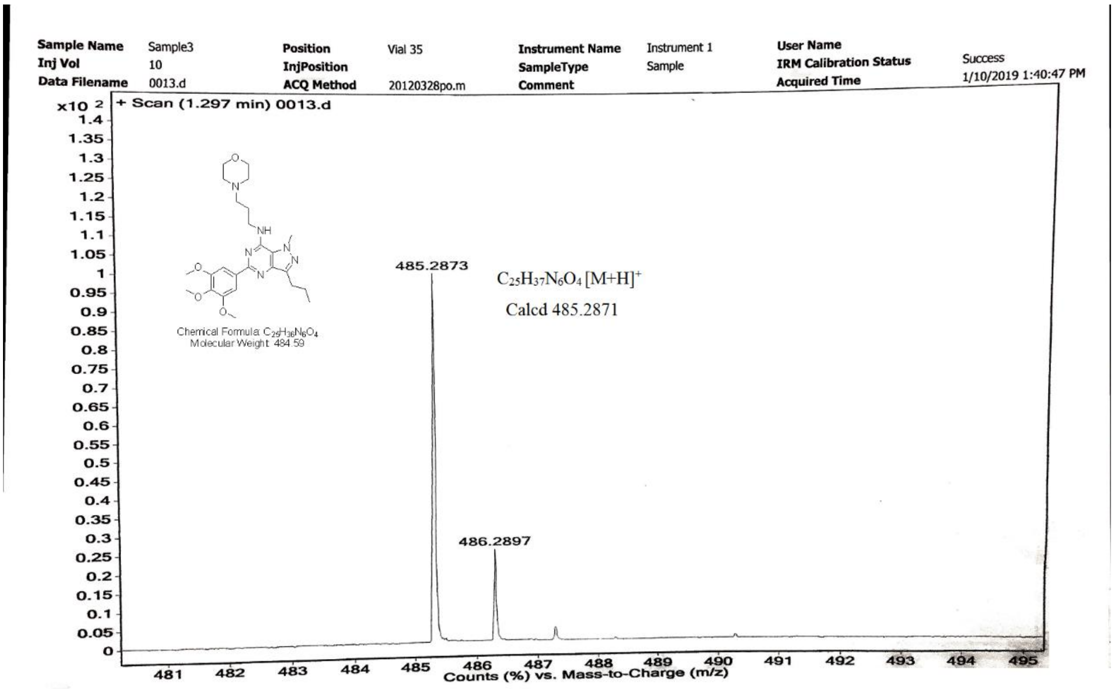

HR-MS data for *1i*

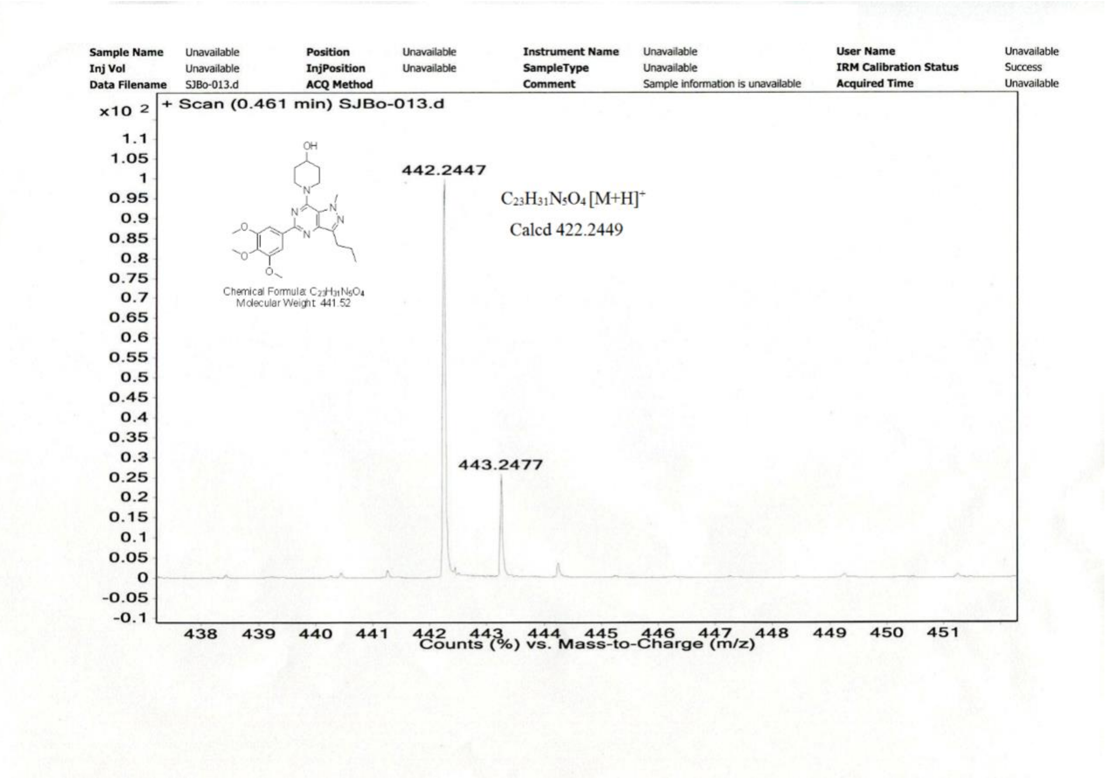

HR-MS data for *1j*

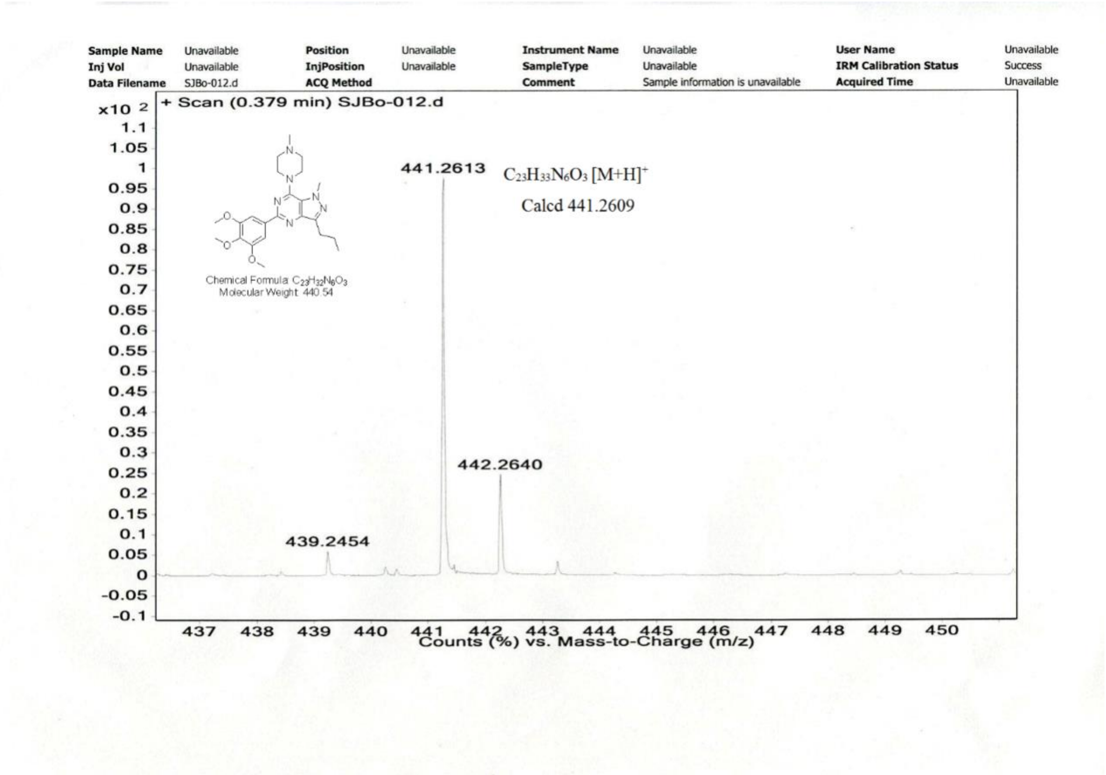

HR-MS data for *2a*

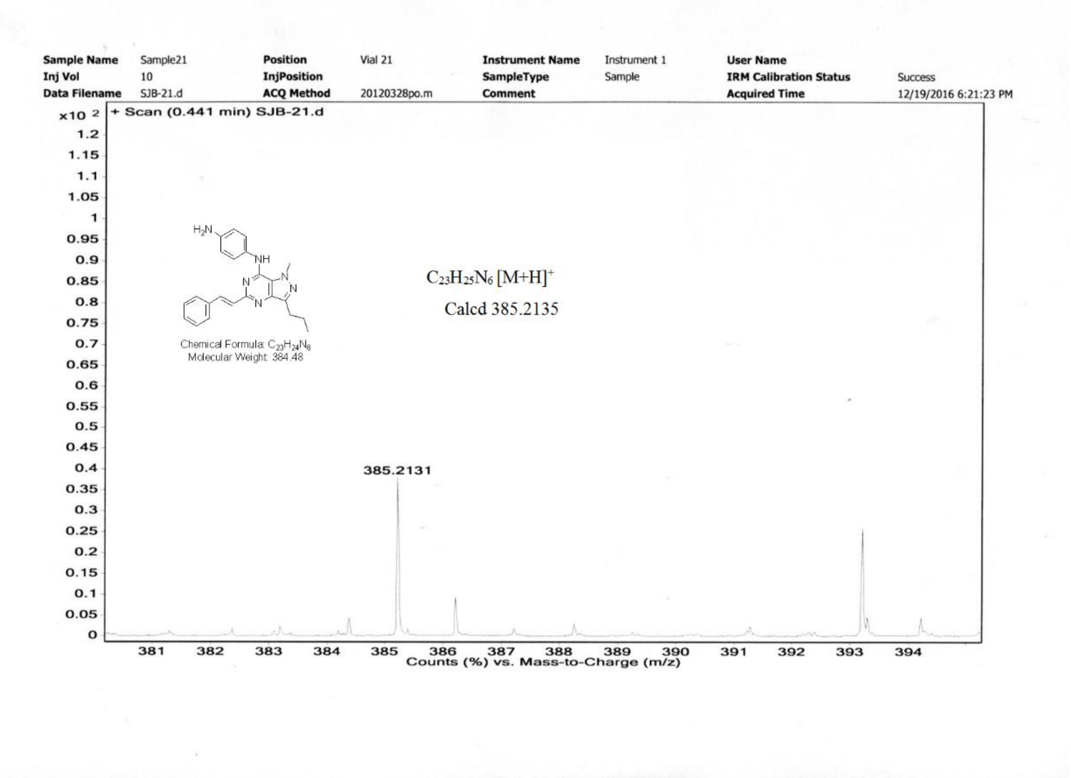

HR-MS data for *2b*

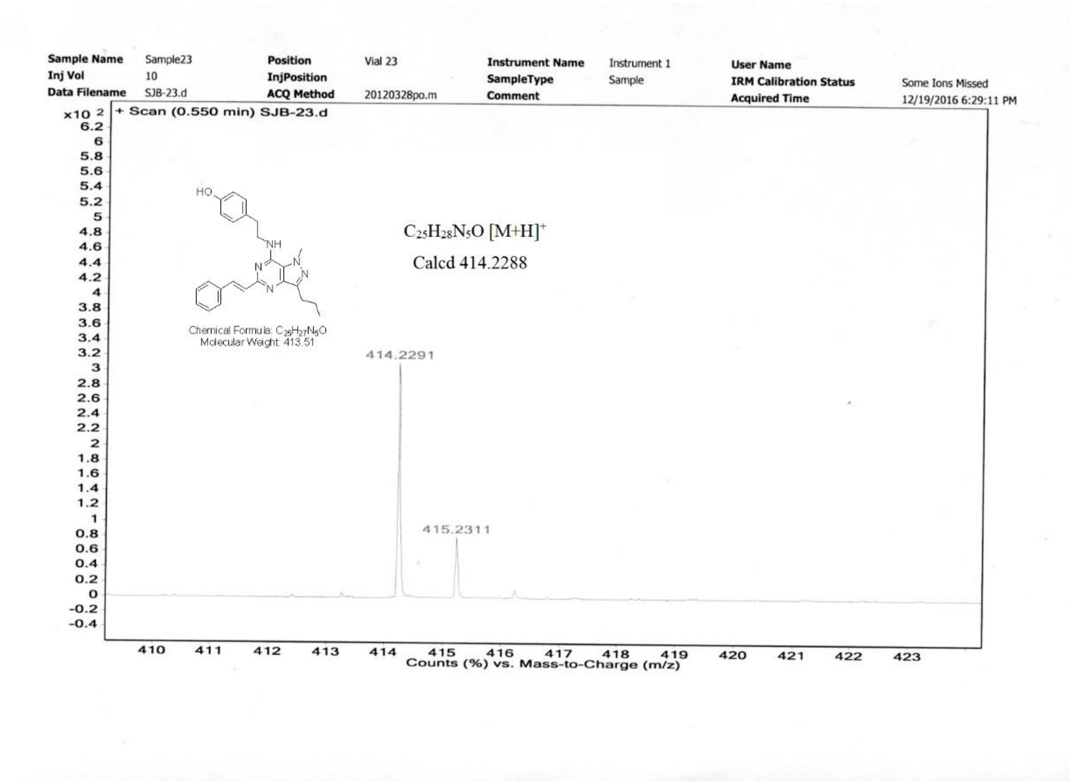

HR-MS data for *2c*

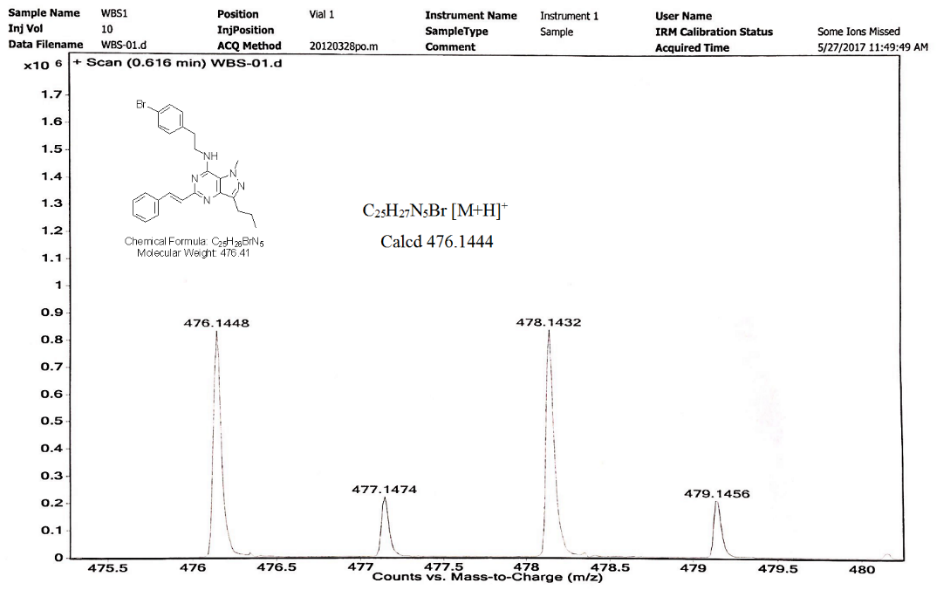

HR-MS data for *2d*

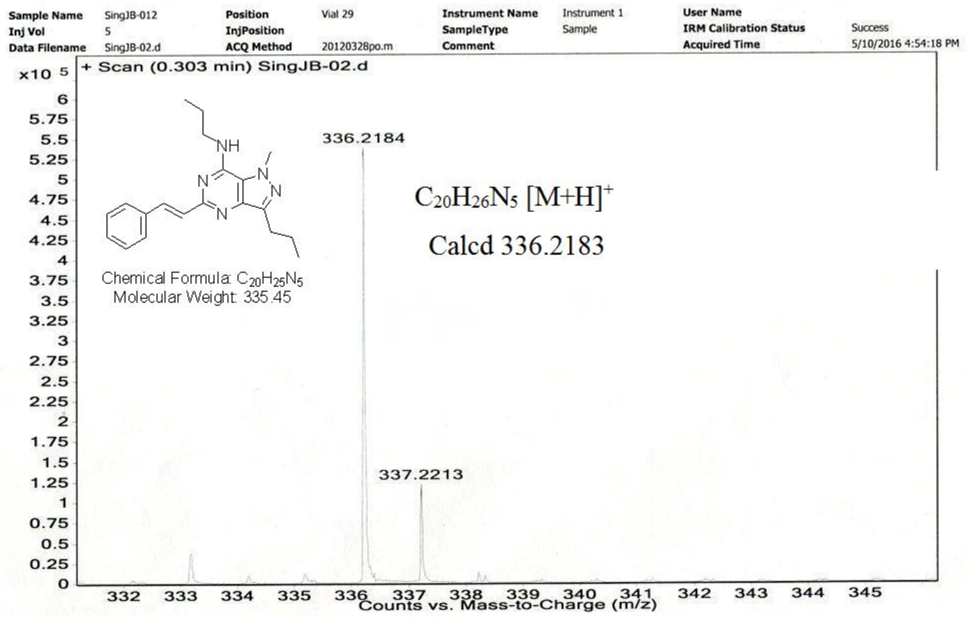

HR-MS data for *2e*

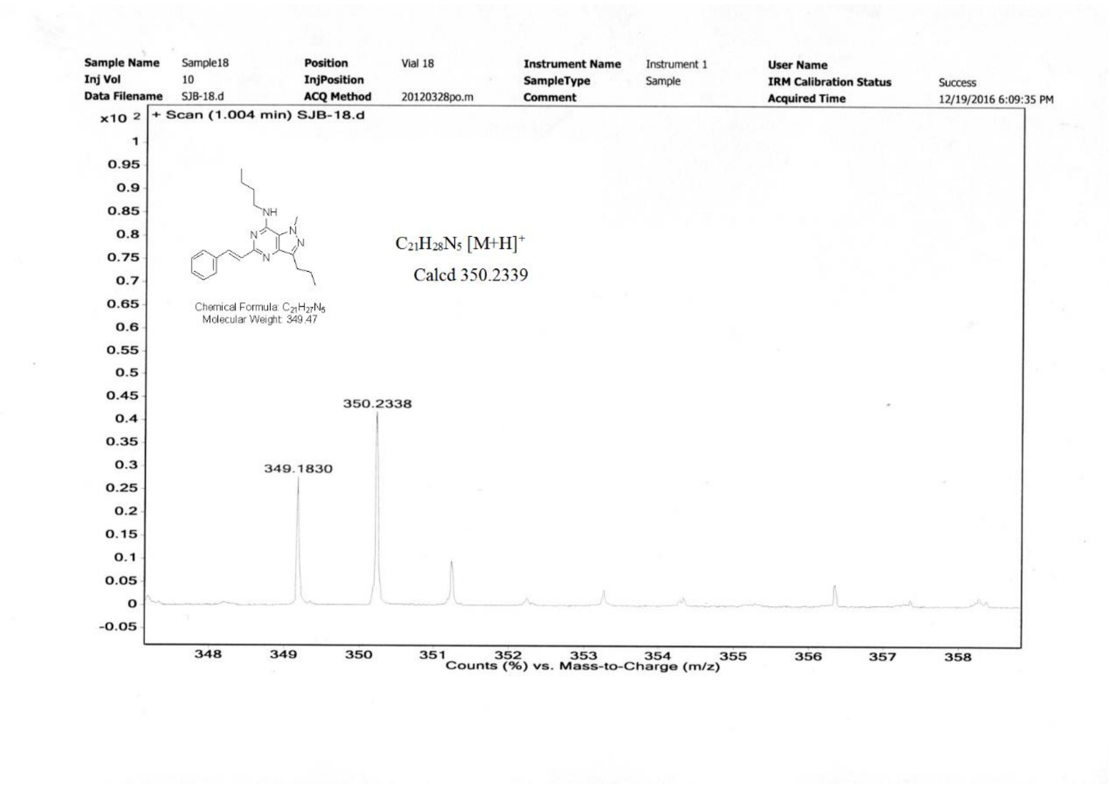

HR-MS data for *2f*

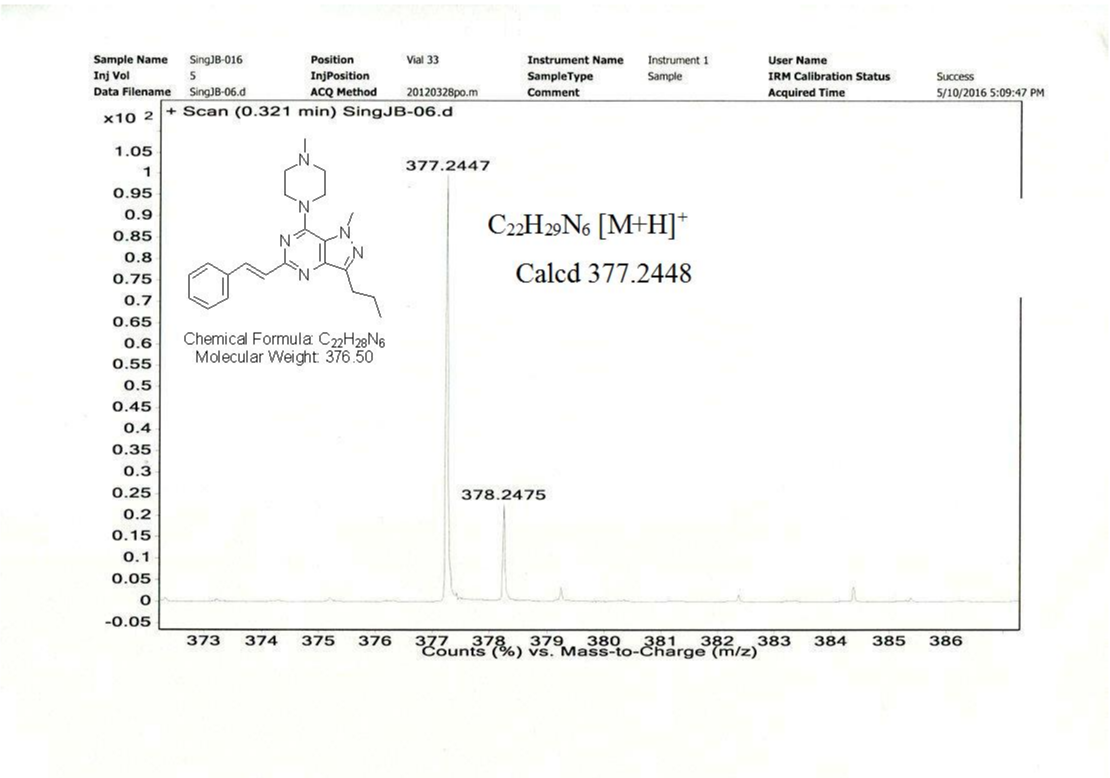

HR-MS data for *2g*

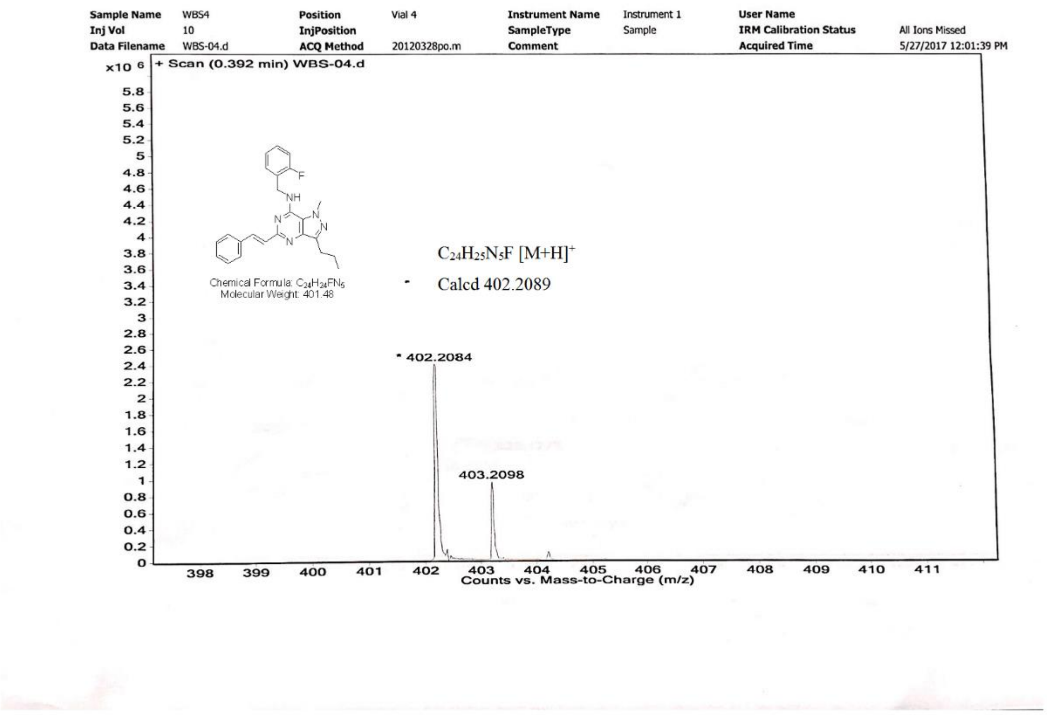

HR-MS data for *3a*

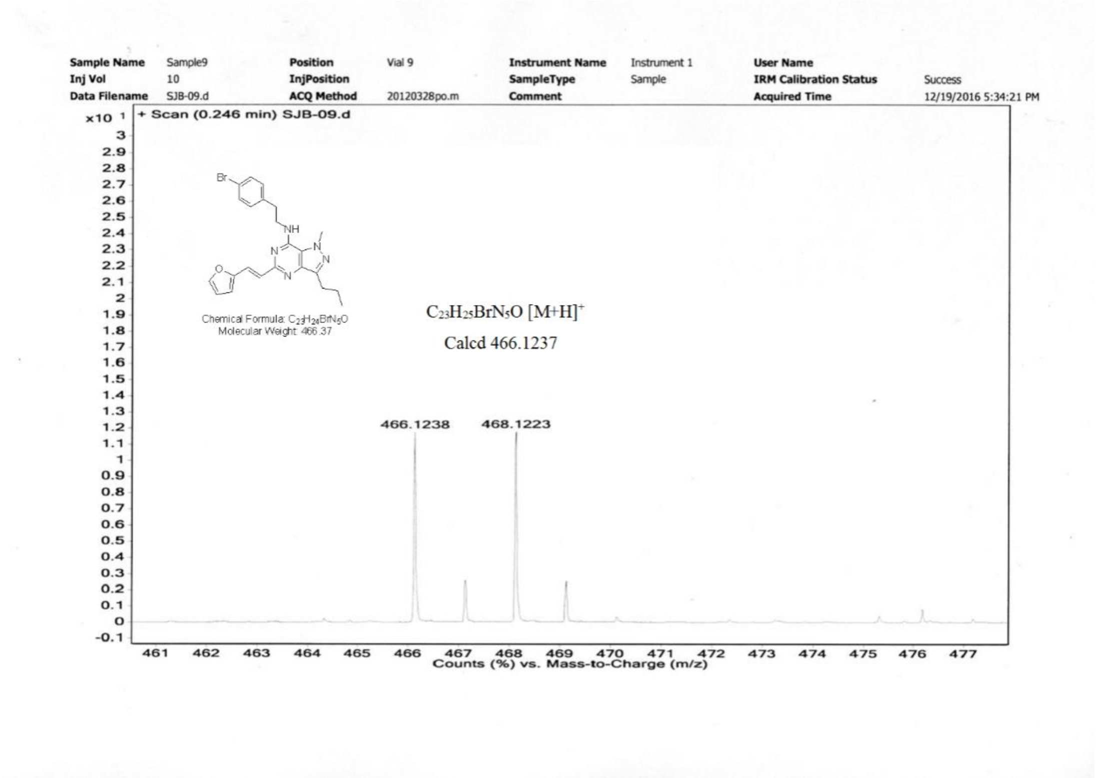

HR-MS data for *3b*

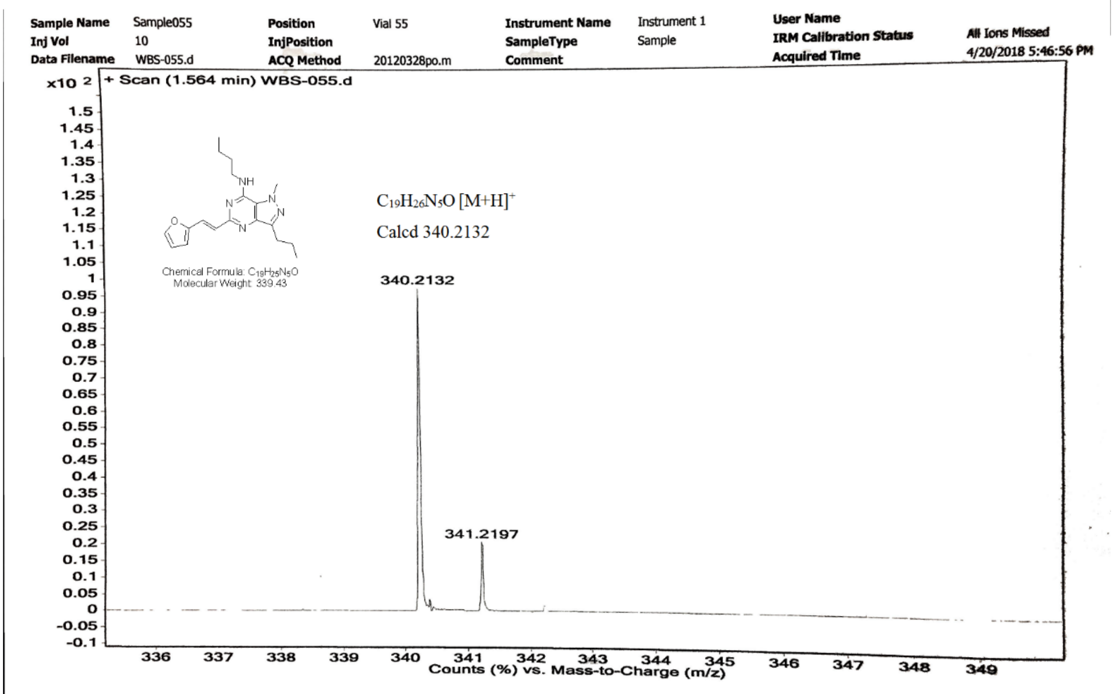

HR-MS data for *3c*

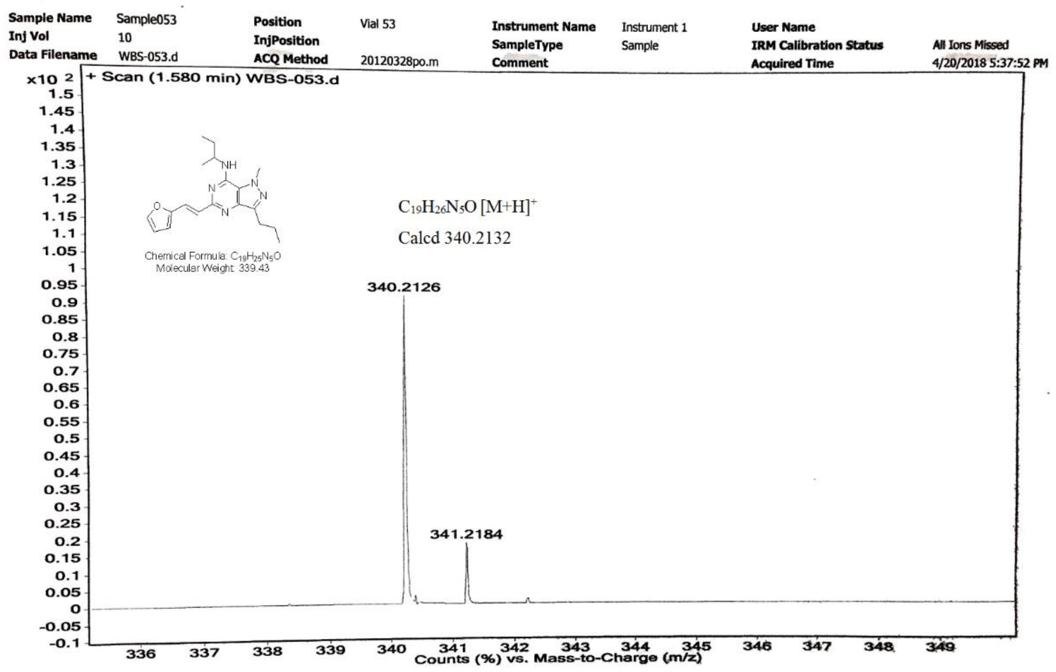

HR-MS data for *3d*

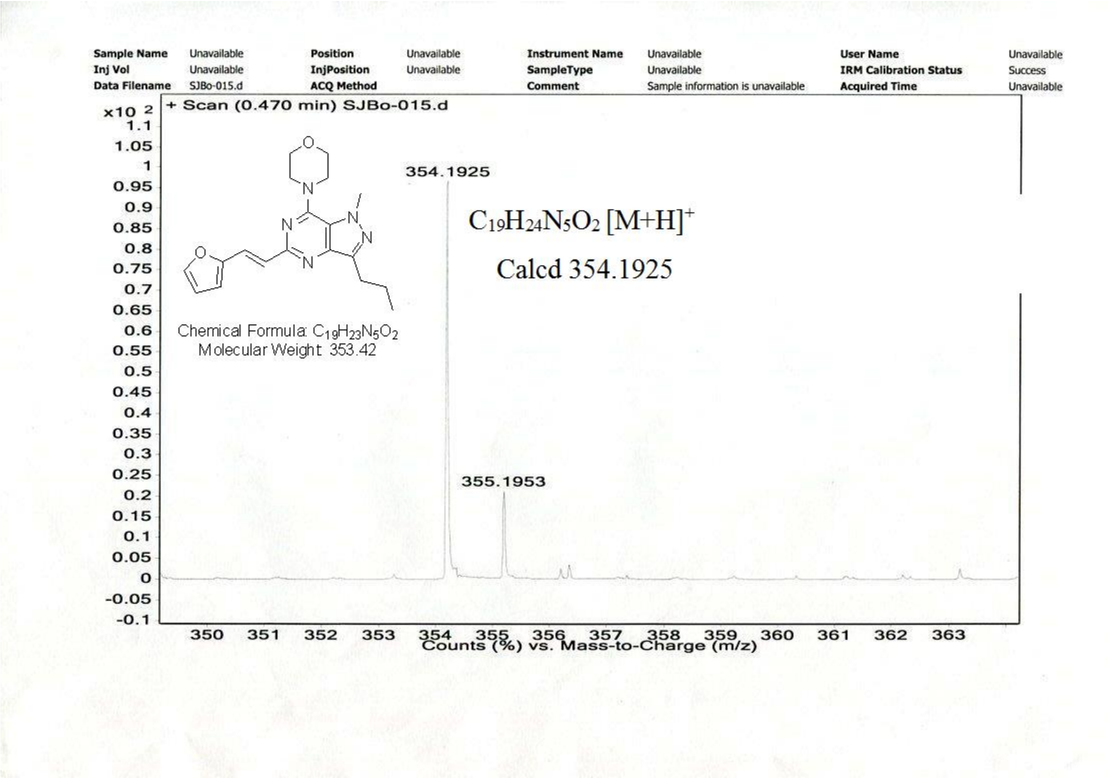

HR-MS data for *3e*

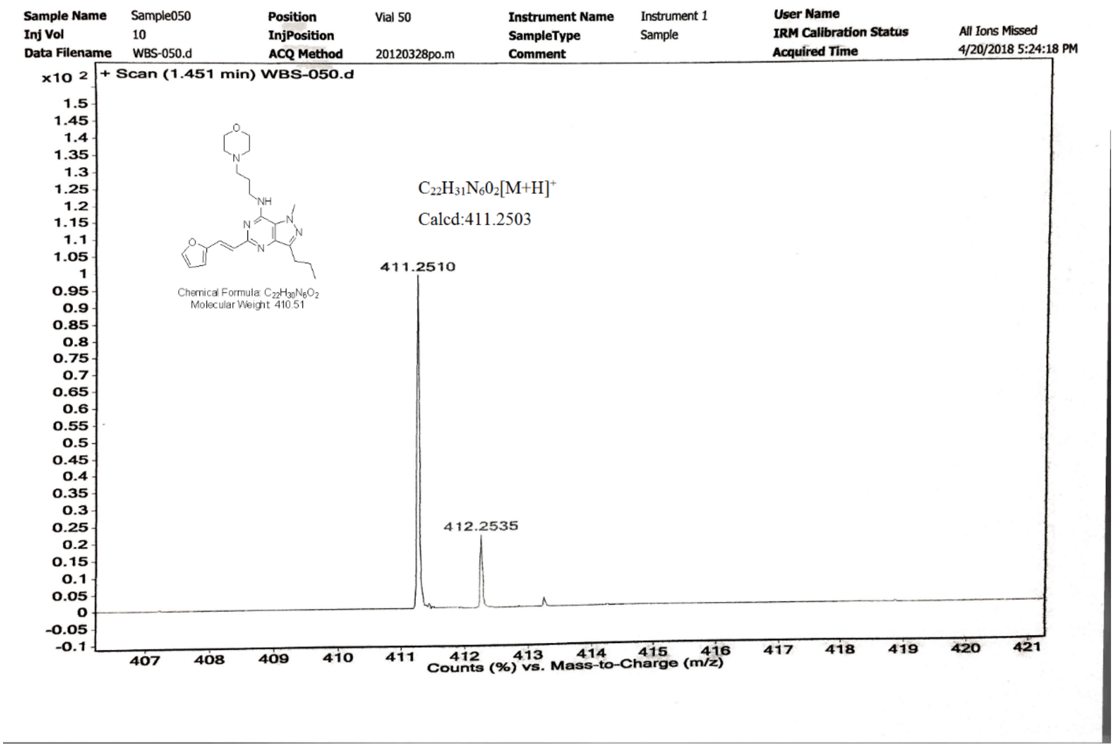

HR-MS data for *3f*

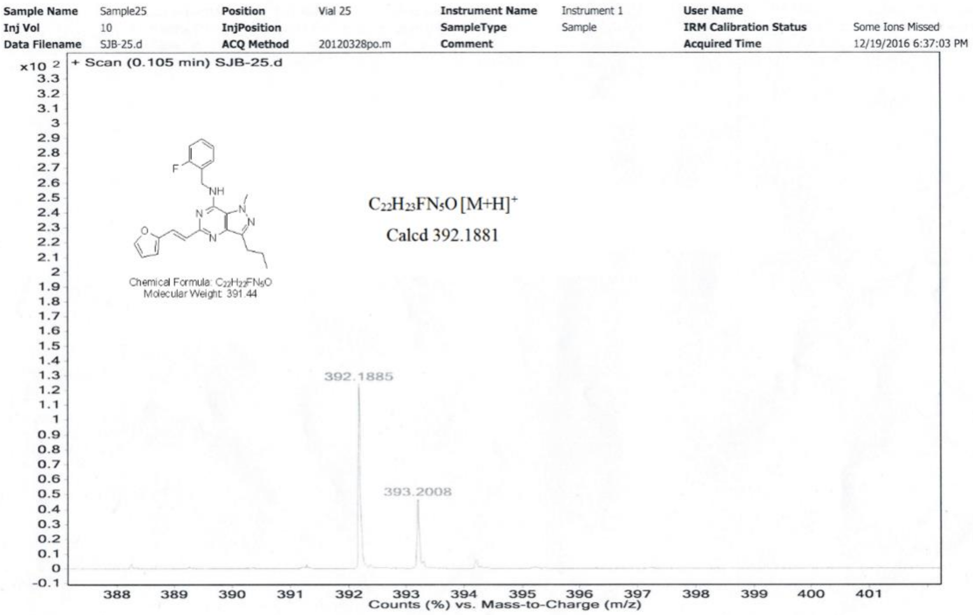

HR-MS data for *3g*

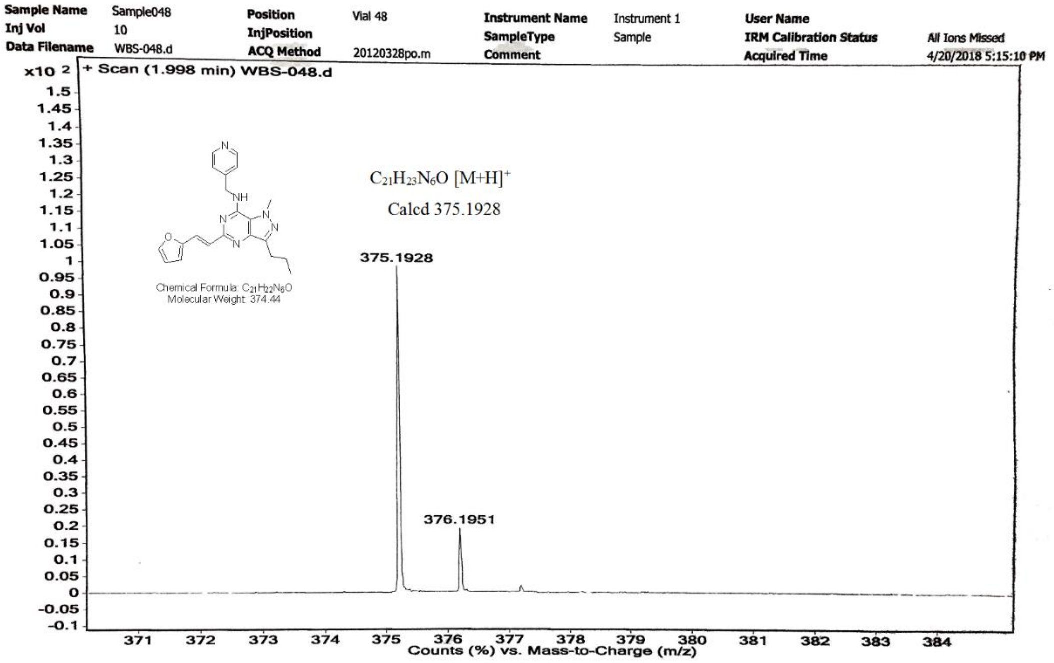

HR-MS data for *4a*

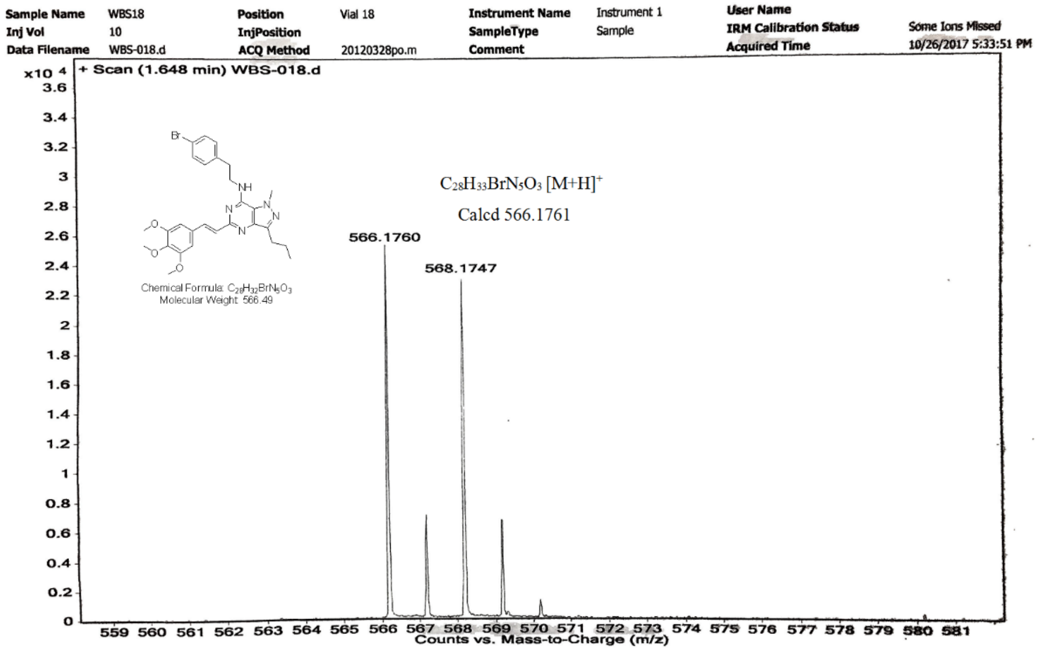

HR-MS data for *4b*

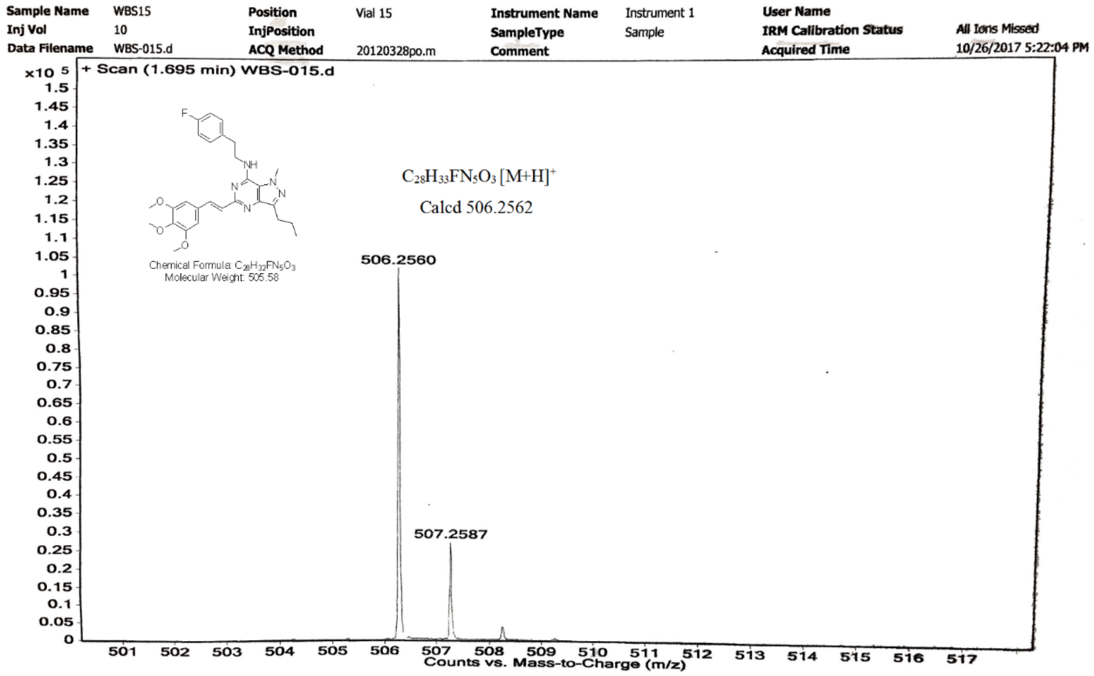

HR-MS data for *4c*

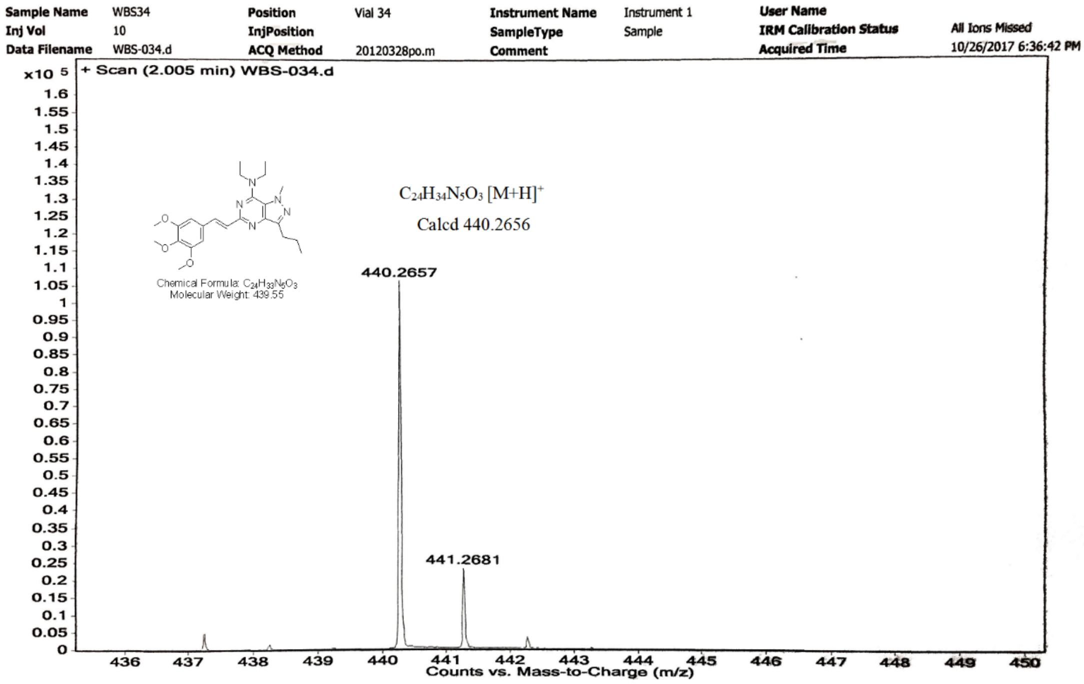

HR-MS data for *4d*

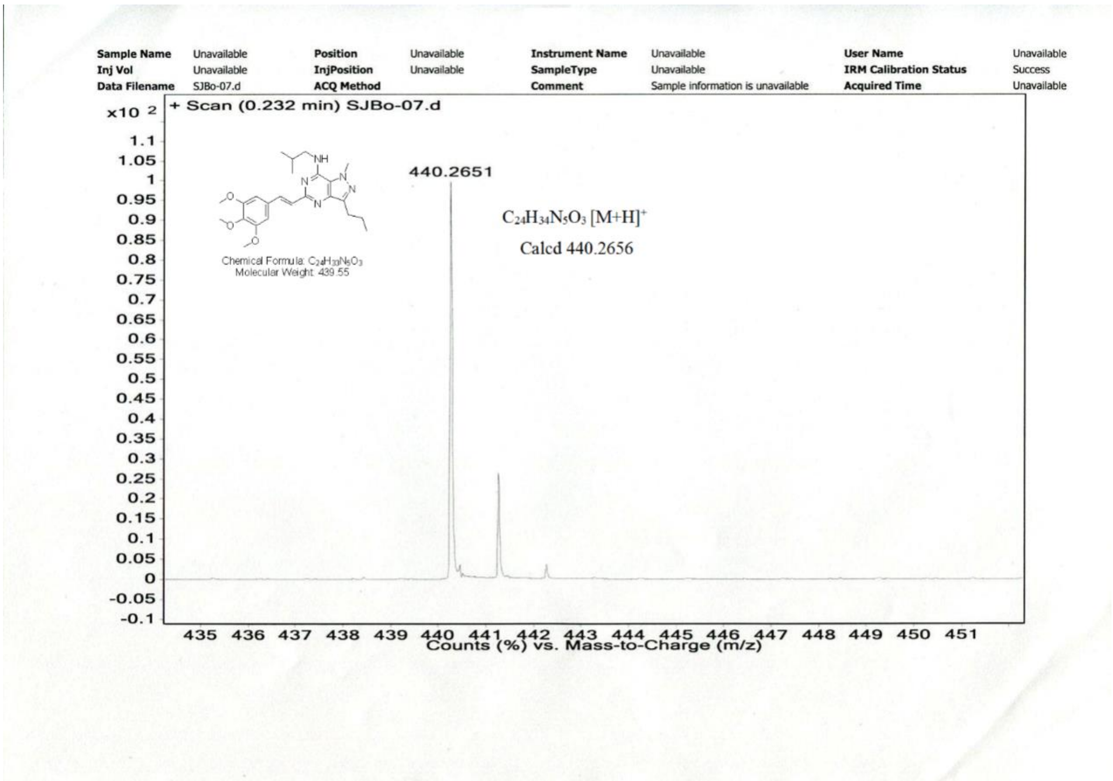

HR-MS data for *4e*

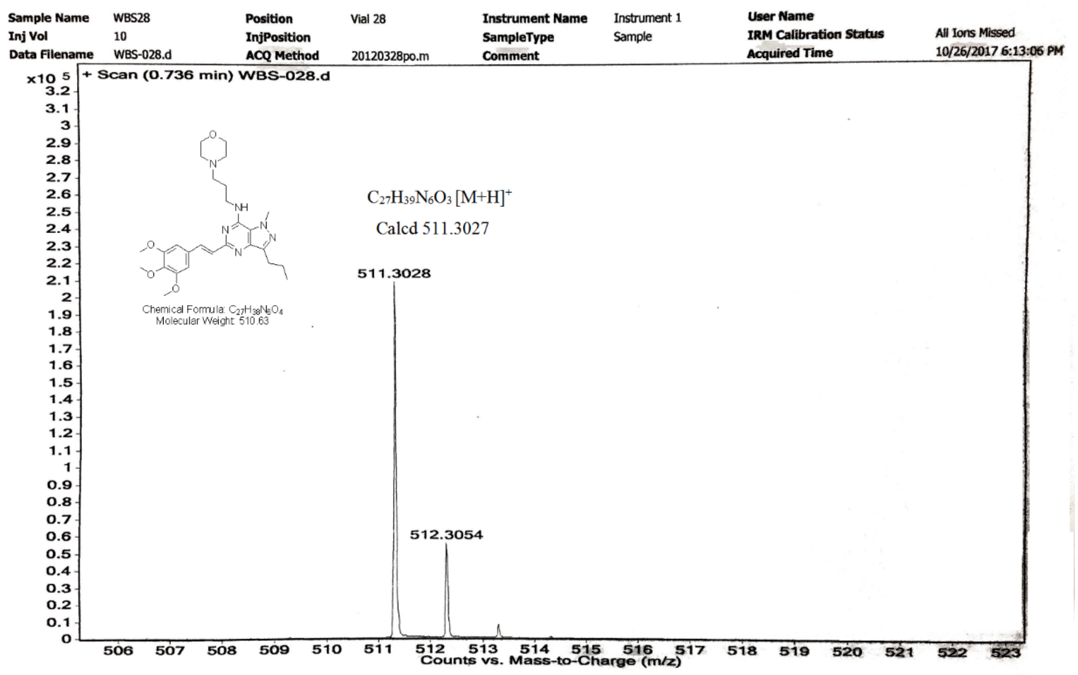

HR-MS data for *4f*

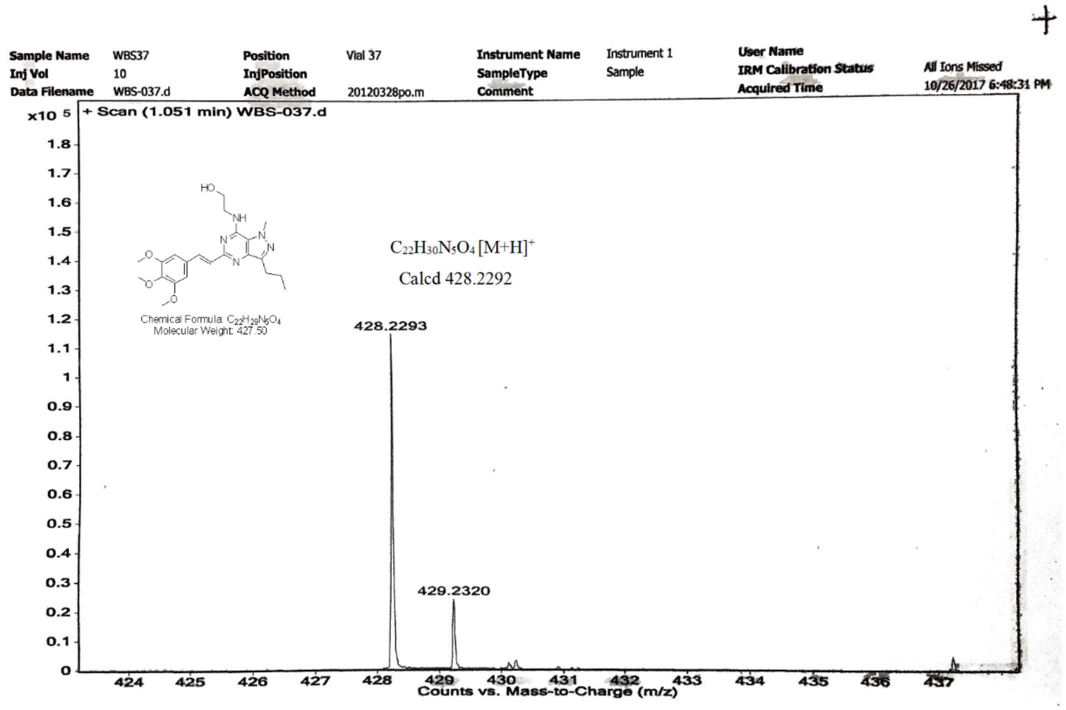

HR-MS data for *4g*

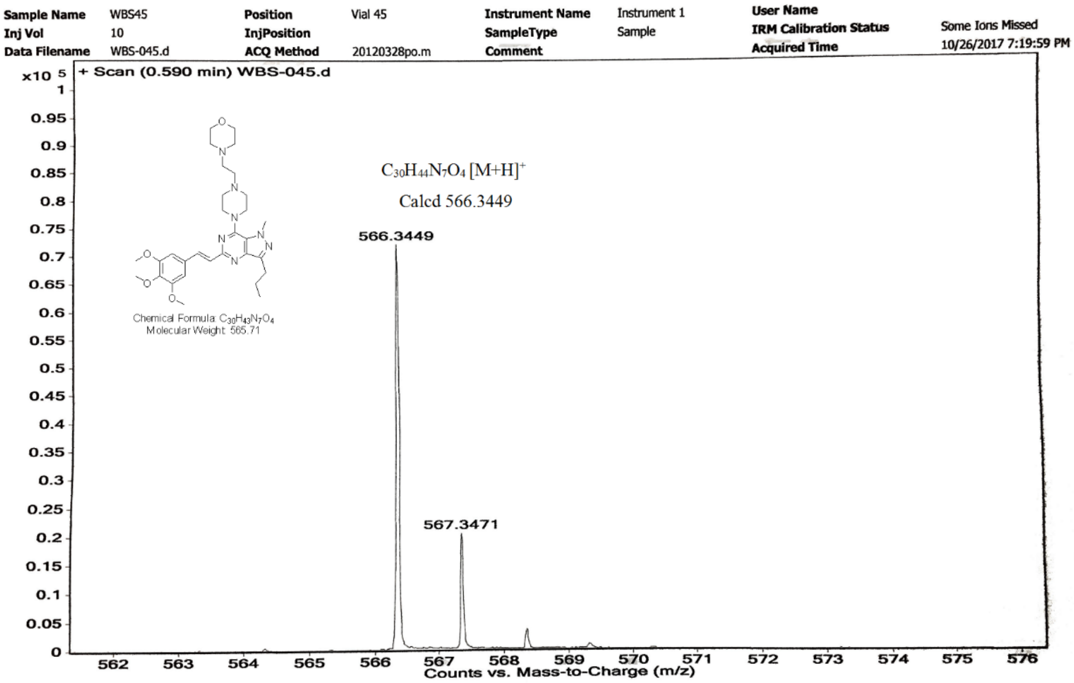

HR-MS data for *4h*

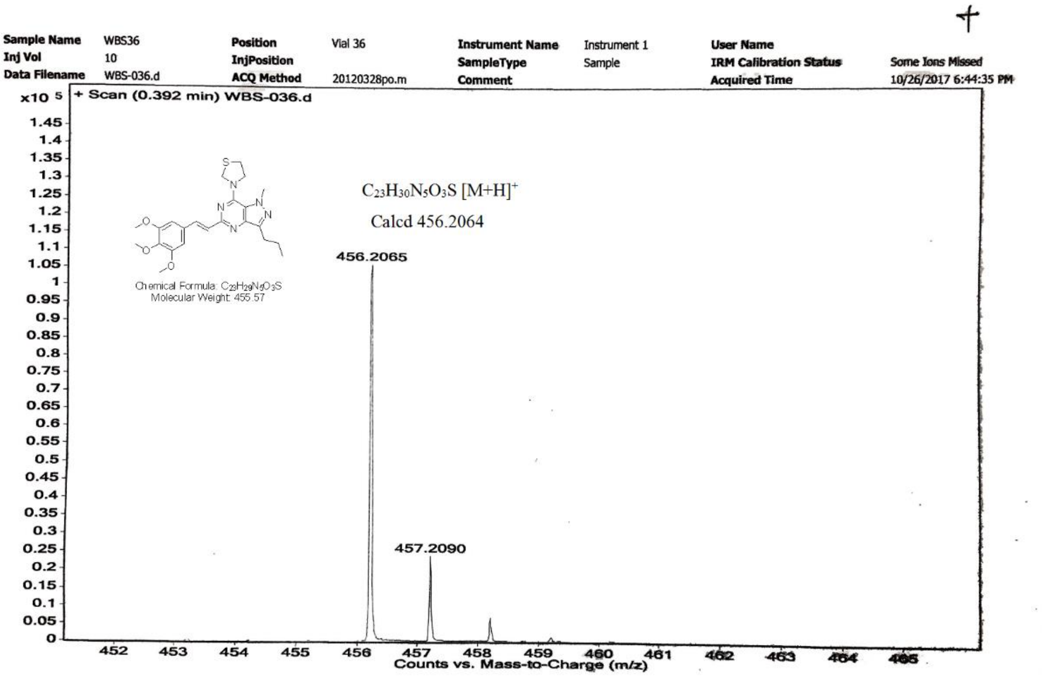

HR-MS data for *4i*

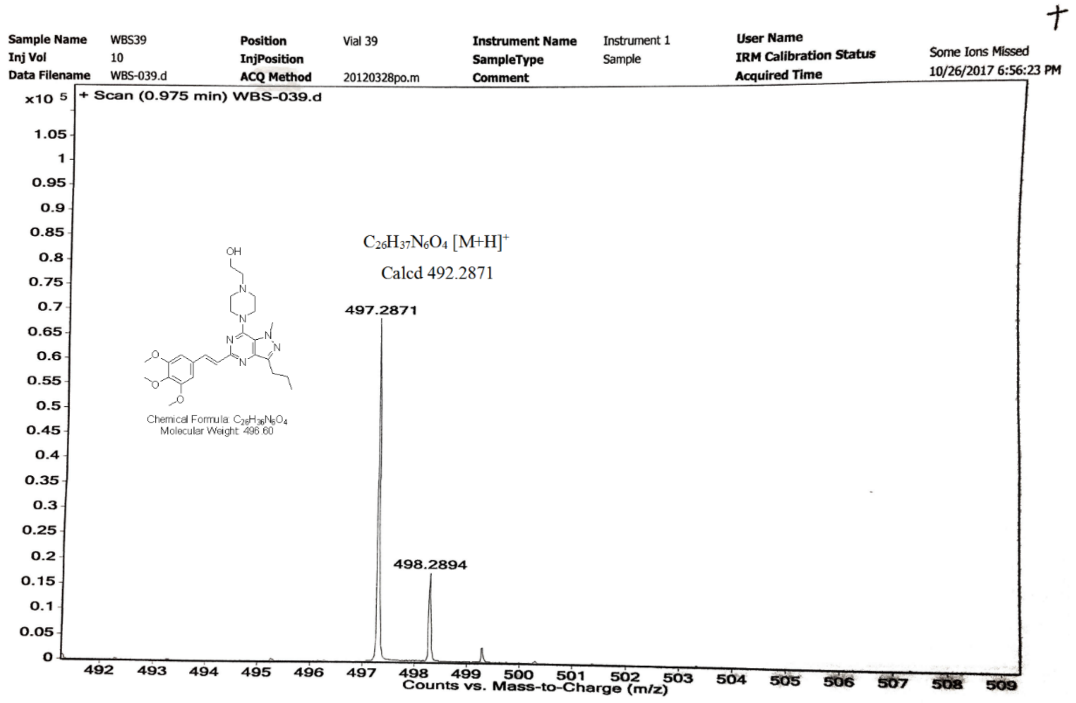

HR-MS data for *4j*

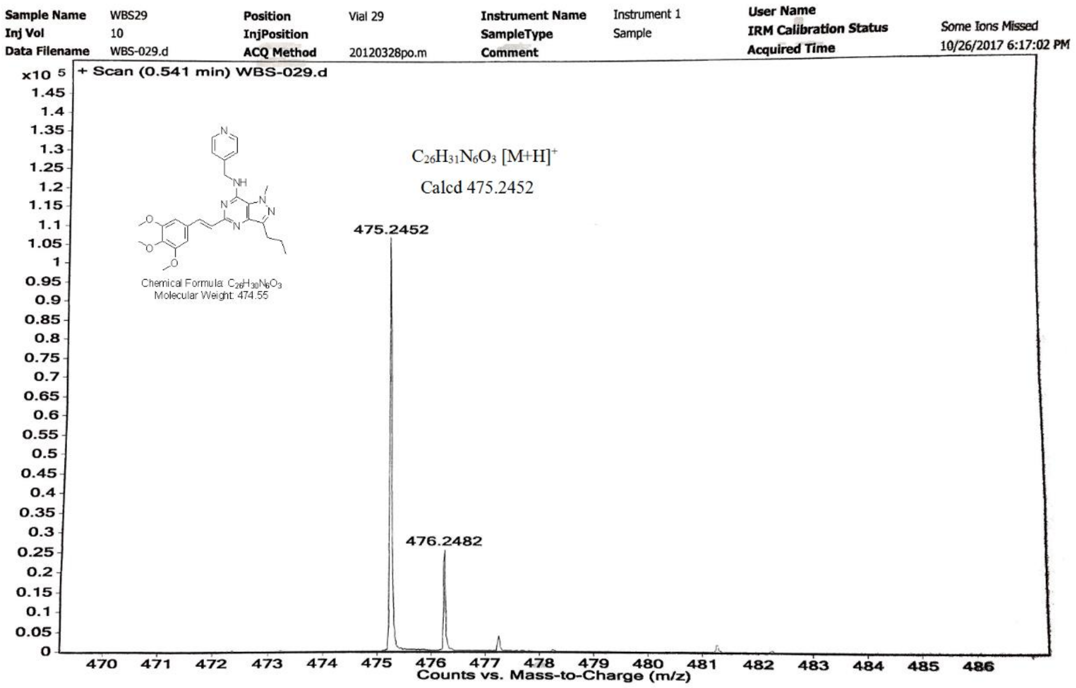

HR-MS data for *4k*

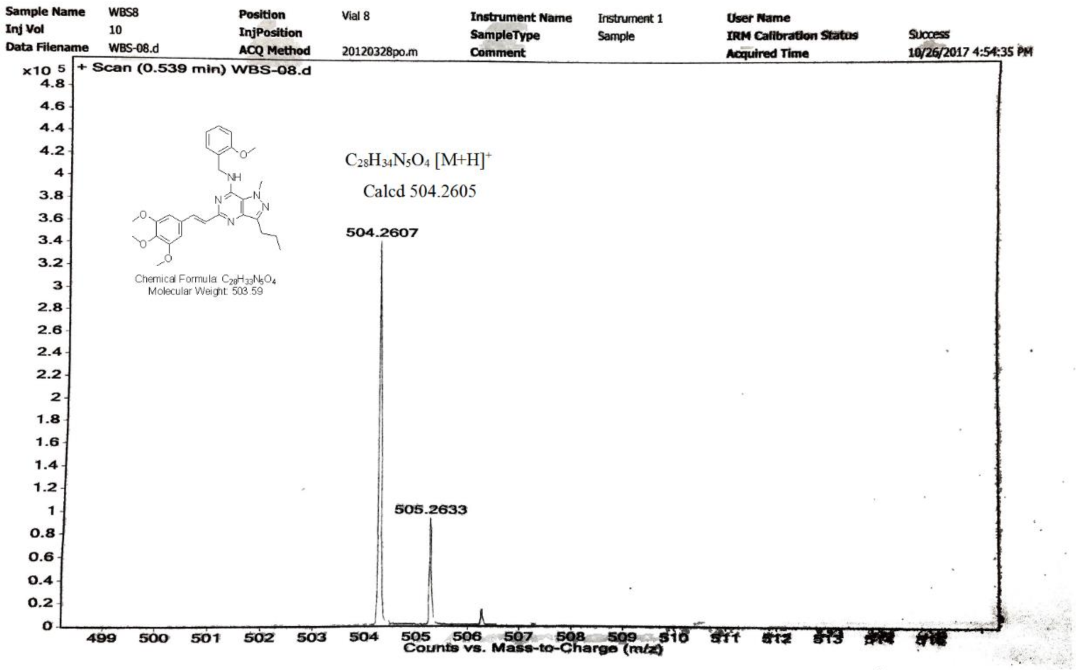

Supplement: Supplemental Material [file IENZ_A_1618291_SM1400.pdf]
